# An efficient debromination technique using PMHS with a number of ligands containing different functional groups

**Published:** 2022-04-07

**Authors:** Md Yeunus Mian, Prithu Mondal, Dishary Sharmin, Kamal P. Pandey, Farjana Rashid, Sepideh Rezvanian, Lalit K. Golani, V.V.N. Phani Babu Tiruveedhula, Vilashini Rajaratnam, Shama P. Mirza, John D. Chan, Jeffrey M. Witkin, James M. Cook

**Affiliations:** 1 Department of Chemistry and Biochemistry, Milwaukee Institute of Drug Discovery, University of Wisconsin-Milwaukee, Milwaukee, WI-53211; 2 Department of Chemistry, University of Wisconsin-Oshkosh, Oshkosh, WI-54901

**Keywords:** debromination, polymethylhydrosiloxane, benzodiazepines, palladium acetate

## Abstract

Herein is described the strategy to debrominate different aryl bromides selectively, using polymethylhydrosiloxane (PMHS) which tolerates a variety of functional groups. Key elements of this approach include the use of catalytic Pd(OAc)_2_ and the correct equivalents of polymethylhydrosiloxane (PMHS), in conjunction with aqueous KF. The present reaction process provides a strategic tool for the synthesis of a number of medicinally important molecules.

## Graphical Abstract

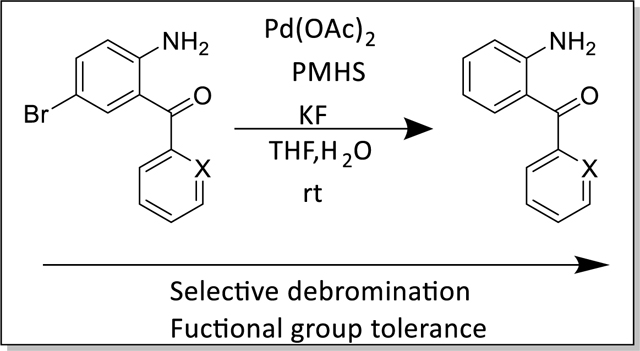

## Introduction

Bromine-substituted aryl halides are important targets for further functionalization catalyzed by palladium catalysts or other chemical strategies.^1^Often times a cheap starting aryl bromide can be the lynch pin in the synthesis of new heterocyclic compounds. Replacement of bromine by hydrogen can be carried out using Polymethylhydrosiloxane (PMHS) reported earlier by Ronald *et al*.^2^ It is a mild reducing agent, cost-effective, non-toxic and an easily handled chemical. It can be stored for long periods of time since it is more air and moisture stable than any other silanes.^3^ PMHS is relatively inert but can transfer the hydride to participate in different metal catalyzed reductions.^4^Hydrosilanes are mild reducing agents with a variety of applications.^25,26^In addition, some hydrosilanes are compatible with some oxidizing agents and act as selective reducing agents in a one-pot redox cocktail.^27^

PMHS hydrodehalogenates aryl halides when using a palladium catalyst.^2^ A method to debrominate aryl bromides in the presence of a number of functional groups such as ketones, amides, imines, and esters was developed. We debrominated bromo-benzodiazepines using this method and then nitrated the resulting product to synthesize pharmaceutically active potential drug candidates.^15^

## Results and Discussion

**Scheme 1.**
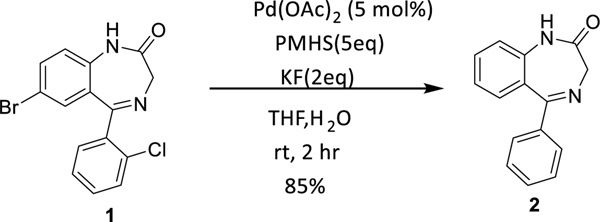
Dehalogenation of benzodiazepine.

Recently, phosphine free palladium catalysts have enjoyed considerable success in different reductive dehalogenations.^6–13^ We required a suitable system to debrominate different benzodiazepine compounds. The investigation initially revealed Pd(OAc)_2_ with PMHS in the presence of potassium fluoride reduced chlorobenzene to benzene,^2^ and this transformation took place at room temperature. We followed the conditions to debrominate a benzodiazepine which bears a bromo and a chloro functionality. The benzodiazepine **1** was synthesized according to the literature procedure.^14^ As expected both halogens were reduced by PMHS (Scheme 1).

**Scheme 2:**
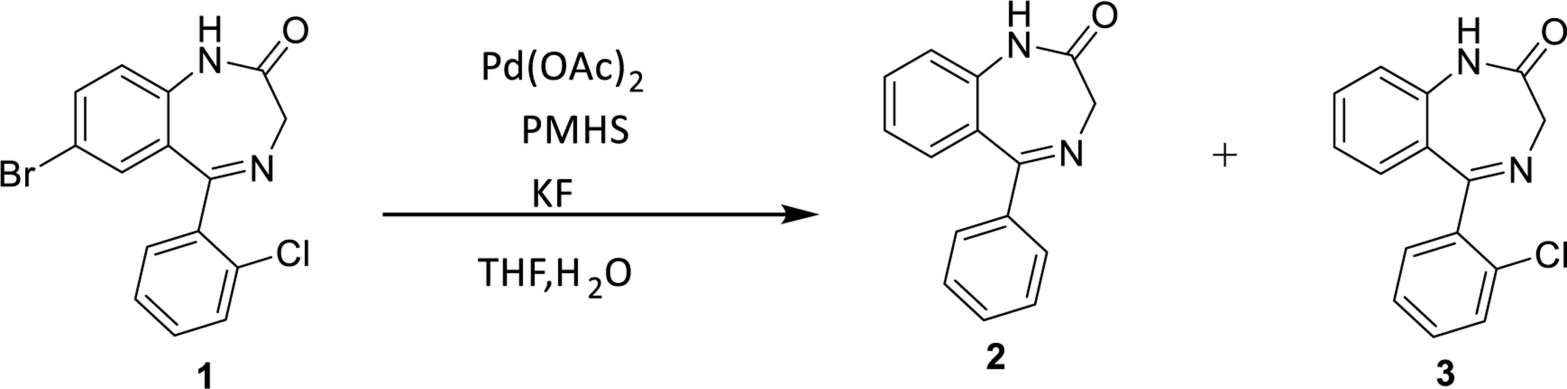


Based on this result, we started to explore this system to find a better method to selectively debrominate the aryl halide without affecting other functional groups. Initially we investigated conditions to determine the correct equivalents of PMHS and reaction time for selective debromination. We used the substrate **1** for our initial investigation (Scheme 2). Different equivalents of PMHS and different mole% of Pd catalyst were used to identify the best conditions. It was found that bromide **1** could be debrominated chemoselectively by reducing the amount of PMHS in the presence of 5 mole% Pd(OAc)_2_; although the reaction time was longer, as expected, and the reaction also gave the byproduct **2** in minor amounts. (Table 1, entry 10; 5:95).

**Scheme 3:**
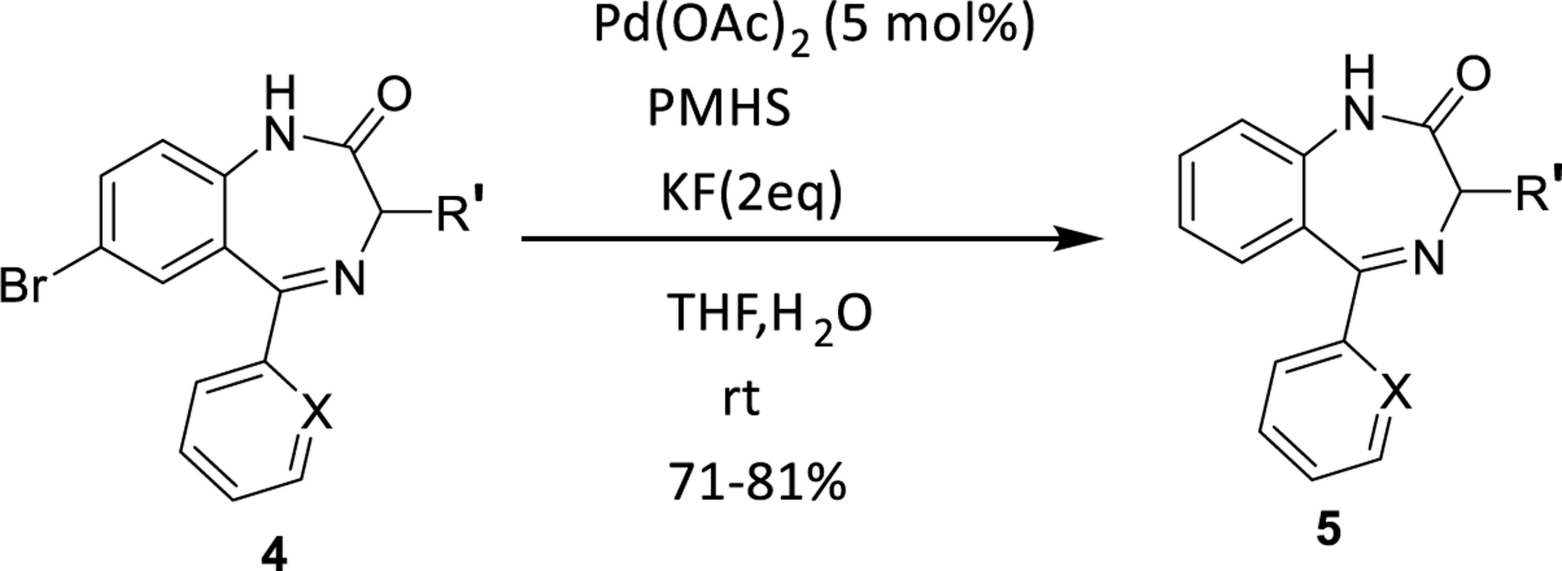


The debromination reaction was also investigated with different benzodiazepines, as shown in Table 2. Benzodiazepine derivatives (**4a-h**) were subjected to debromination under the same conditions (Scheme 3, Table 2) to furnish the products **5a-h** (71%−81%), respectively. We also increased the amount of PMHS to debrominate the substrate **4e** to produce **5e** (Table 2, entry 5). The debromination occurred smoothly in less reaction time without affecting the amide and imine functionality of the benzodiazepine substrates. A similar trend was also observed in the case of substrate **4h**. Here an interesting observation was found. Although the acetate is a labile protecting group to reduction, it survived the debromination reaction process by PMHS. The hydride source did not interfere with the acetate moiety.

**Scheme 4:**
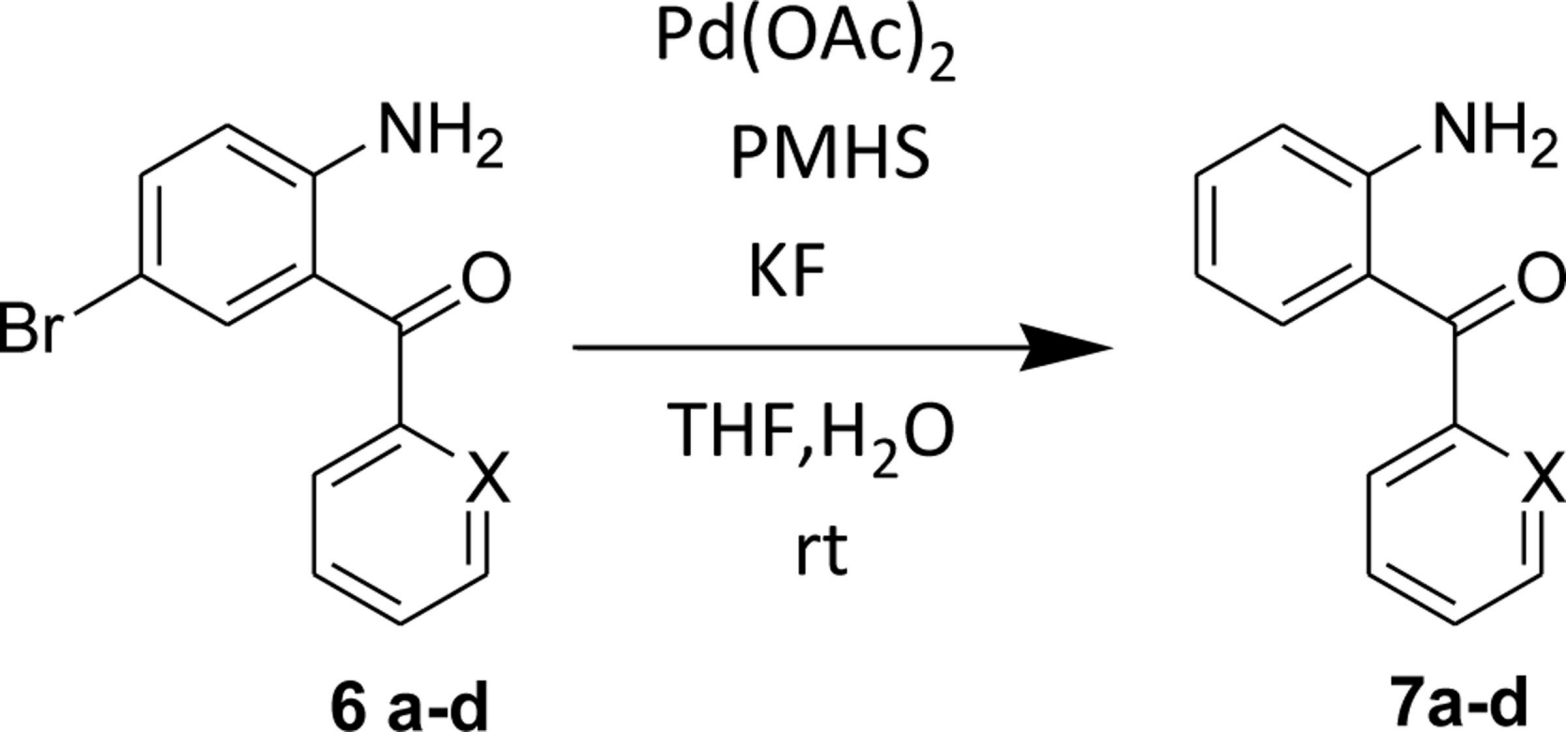


We, then identified conditions in different benzophenone substrates, as shown in Table 3 (Scheme 4). The treatment of other benzophenone substrates afforded debrominated product **7a-d** in good to high yields (Table 3). We also observed that increasing the amounts of PMHS decreased the reaction time in some benzophenone substrates (**6a-b**), as expected. PMHS did not affect the ketone functionality in any benzophenone substrate (Table 3, entry 1–8), as we hypothesized. Selective debromination over chlorination was also observed in this case (Table 3, entry 3), which is also as expected and, gratifyingly, took place.

**Scheme 5.**
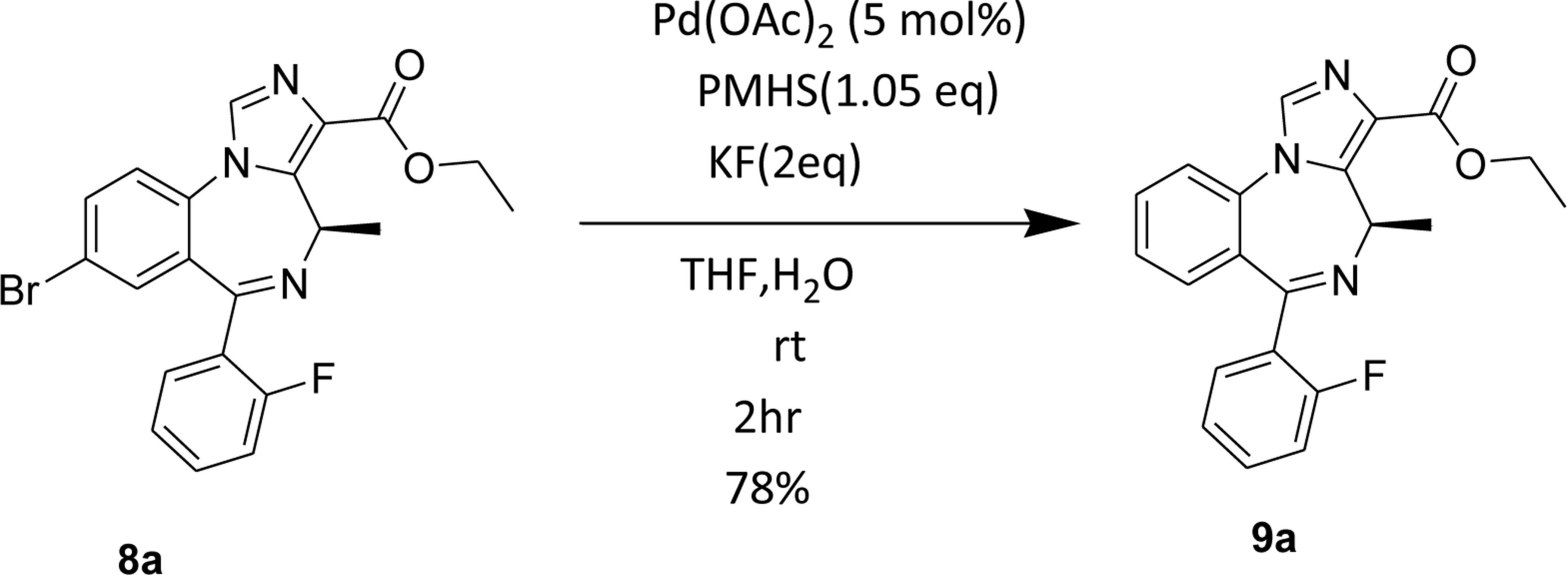
Effect of the debromination reaction on imidazodiazepine substrate **8a.**

**Scheme 6.**
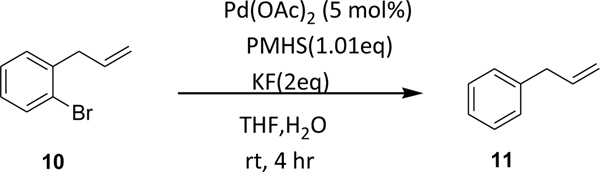
Selective debromination by PMHS on a double bond containing substrate.

Furthermore, we attempted the debromination on an imidazodiazepine substrate possessing an ester function (Scheme 5). The imidazodiazepine **8a** gave the debrominated product **9a** in good yield. The imidazodazepine ester was well tolerated under these reaction conditions confirming the mildness of the PMHS process. Also, upon investigation on a double bond containing aryl substrate, PHMS debrominated the bromine group selectively without interfering with the double bond (Scheme 6, preliminary data, SI)

### Anti-schistosomal activity of 8-*H* benzodiazepines

Schistosomiasis, a neglected tropical disease is one of the most dominant infectious diseases worldwide that has serious public health consequences ^16–19^and is characterized by chronic helminthic infection with residual morbidity. Over 240 million people are infected, with 800 million at risk of infection and more than 90% of infections occurring in sub-Saharan Africa where the death rate is approximately 280,000 persons per year.^15^ Schistosomiasis is caused by parasitic blood flukes (trematode worms) of the genus *Schistosoma*, of which *Schistosoma mansoni*, *Schistosoma japonicum and Schistosoma haematobium* cause infections in humans.^20^

The treatment of schistosomiasis relies only on one broad spectrum drug, praziquantel which is associated with several drawbacks including PZQ’s lack of efficacy against immature parasites. Meclonazepam, a benzodiazepine was found to be effective in treatment of schistosomiasis^15^and was able to cure both mature and immature infections. But the curative dose of meclonazepam causes dose-limiting sedation in human trials and the development of this lead was dropped.^21–24^

**Figure 1.**
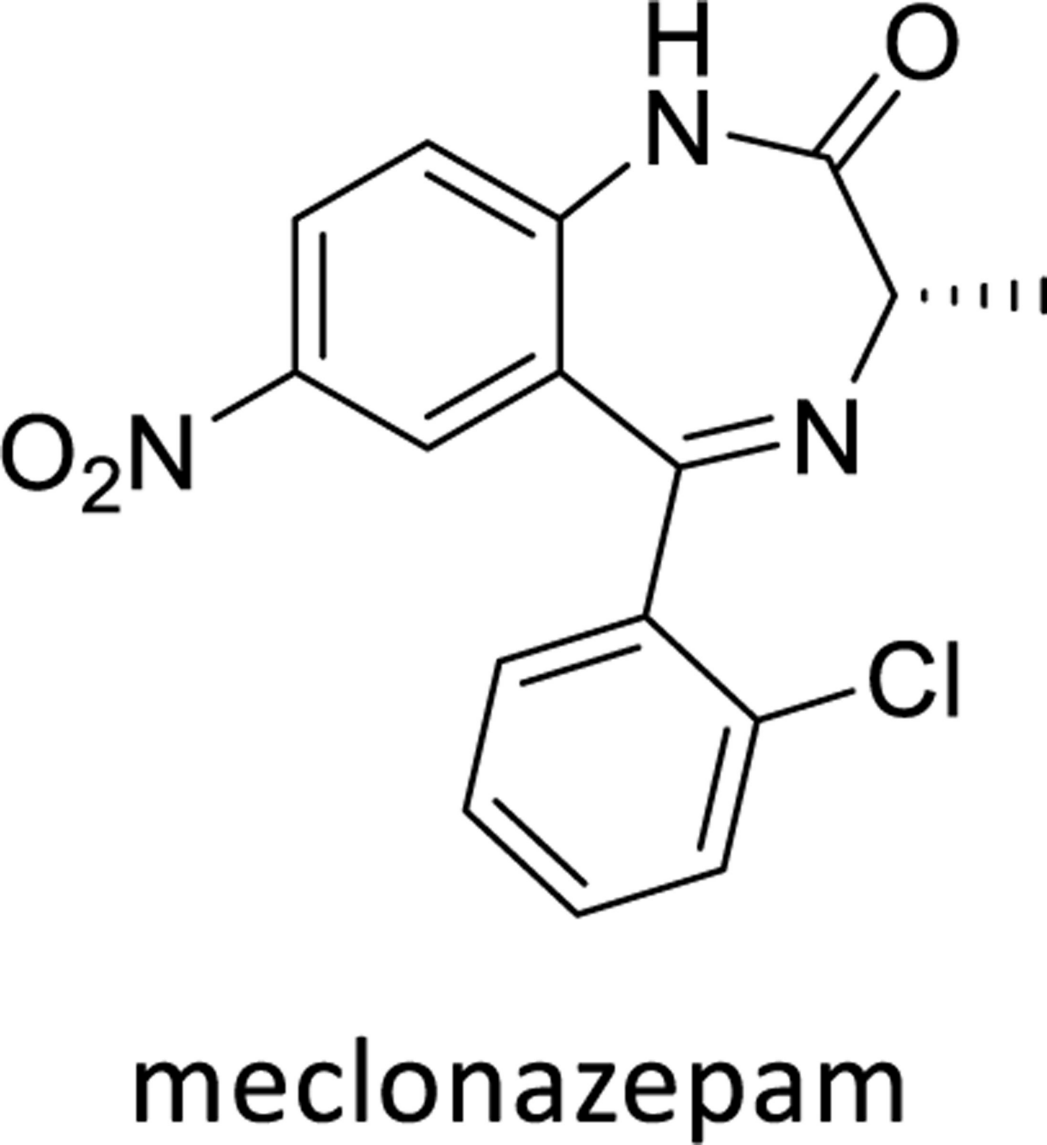
Structure of meclonazepam.

The chemical name of meclonazepam is (S)-5-(2-chlorophenyl)-3-methyl-7-nitro-1,3-dihydro-2*H-*benzo[*e*][1,4]diazepin-2-one and the structure bears a nitro group in one of the benzene rings (Figure 1). We synthesized different benzodiazepines (most of them in this manuscript) which contained 8-H instead of 8-nitro groups and assessed the synthesized compounds in schistosome mobility assays. This assay was done in order to identify the importance of the nitro group in the structure of the benzodiazepines in regard to activity. The results are summarized in Table 4.

The IC_50_ value of meclonazepam is 160 nM and it causes contractile paralysis of the parasites. The parasites were coiled, dead upon the exposure to the meclonazepam (Table 4). But changing the nitro group to hydrogen resulted in total loss of activity (entry 2, Table 4). Changing the nitro group to a hydrogen atom along with other modifications demonstrated no activity at all (entry 3–7, Table 4). Consequently, a hydrogen atom at the 8-position in place of a nitro group is not suitable for anti-schistosomal activity. In order to identify whether the electronic effects of the nitro group or the structure of nitro group itself was responsible for schistocidal activity, we replaced the nitro group with a cyano group and assessed the compound’s activity in the schistosome mobility assay. This resulted in total loss of activity. This would argue, although not prove, against a pi stacking interaction with the ring-A aromatic ring in meclonazepam. Modification of different positions without changing the nitro group show various degrees of anti-schistosomal activity and some of these results are described in McCusker P. *et al*. 2019.^15^ The nitro group in the structure of meclonazepam is crucial for the damage to the parasite.

## Conclusions

It was demonstrated that ketones, amides, esters and imines were all well tolerated under the reduction conditions of PMHS employed here with catalytic Pd(OAc)_2_ in THF. This is an efficient system for the debromination of benzodiazepines, benzophenones and imidazodiazepines at room temperature. The debromination can be done with a small amount of Pd catalyst but 5 mol % still represents a general catalyst loading. It was also shown that debromination occurred selectively in the presence of a chloro functional group, which is key for the synthesis of some novel potential drug candidates.^15^

## Experimental Section

### General.

All reactions were performed in round-bottom flasks with magnetic stir bars under an argon atmosphere. Organic solvents were purified when necessary by standard methods or purchased from Sigma-Aldrich Chemicals. The reagents and other chemicals were purchased from either Sigma-Aldrich, Oakwood Chemical, Alfa Aesar, Matrix Scientific, Admiral Chemical Company, or Acros Organic. The progress of reactions was visualized with TLC plates from Dynamic Adsorbents, Inc. under a UV light. The flash column chromatography was done for purification of some analogs on silica gel (230–400 mesh, Dynamic Adsorbents). An Agilent HPLC was used to determine the ratio of some debrominated products. The ^1^H NMR and ^13^C NMR spectra were obtained on Bruker Spectrospin 500 MHz instrument in CDCl_3_ and chemical shifts were reported in δ (ppm). Multiplicities are represented as follows: singlet (s), broad signal (br), doublet (d), triplet (t), quartet (q), dd (doublet of doublets), and multiplet (m). The technique employed for HRMS was carried out on a LCMS-IT-TOF at the Milwaukee Institute for Drug Discovery in the Shimadzu Laboratory for Advanced and Applied Analytical Chemistry.

### General procedure for the debromination:

A three neck round bottom flask was charged with the aromatic halide. Then THF was added and the flask was purged with argon. Palladium acetate was then added under an argon atmosphere, followed by the addition of aq potassium fluoride solution. Then PMHS was added dropwise to the reaction mixture, which resulted in a deep greenish solution. The reaction mixture was then stirred for the required time by monitoring by TLC (see Tables for data). The consumption of starting material was confirmed on a LCMS 2020. The round bottom flask was open to the air at the end of the reaction. 2 Grams of alumina was added to the reaction flask and this was allowed to stir for 5 min. The reaction mixture was filtered through a pad of celite to removethe Pd salts; water and ethyl acetate was added to the filtrate. The layers were separated and the aq layer was extracted with ethyl acetate and the combined organic layer was washed with aq 10% NaCl solution (2x), dried over sodium sulfate, concentrated under reduced pressure and purified via silica gel column chromatography. The gradient elution was effected by ethyl acetate and hexane. The purified compounds were characterized by NMR spectroscopy, LCMS and HRMS (individually).

### 5-Phenyl-1,3-dihydro-2*H*-benzo[*e*][1,4]diazepin-2-one (2)


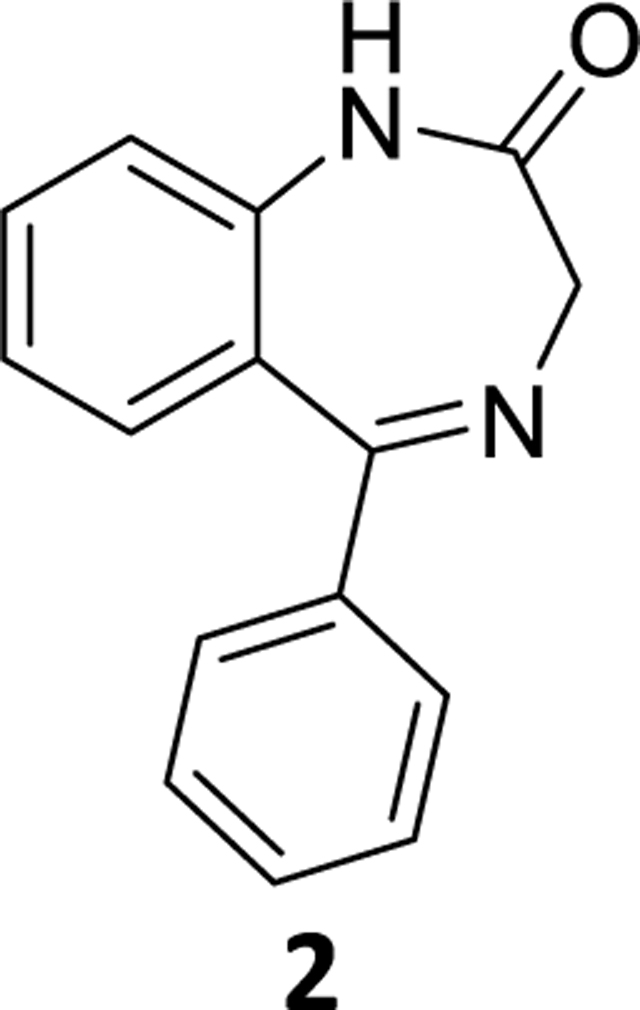


The Pd(OAc)_2_ (0.04 g, 0.12 mmol) was added to a stirred solution of 7-bromo-5-phenyl-1,3-dihydro-2H-benzo[e][1,4]diazepin-2-one (**4**) (0.8 g, 2.53 mmol) in THF (10 mL) under argon. This was followed by addition of aq potassium fluoride ( 0.293 g, 5 mmol in 2 mL water) solution and PMHS (0.16 mL, 2.65 mmol). The flask was opened to air after the required reaction time (table 2), stirred with alumina and then filtered through celite. The aq layer was extracted with ethyl acetate (2×10mL) and the combined organic layer was washed with aq 10% sodium chloride solution (2×10mL), dried over Na_2_SO_4_ and, concentrated under reduced pressure. The residue was purified by silica gel column chromatography using EtOAc: hexane (35:65). The appropriate fractions were pooled, the solvents were removed under reduced pressure and the residue was dried under vacuum to obtain a white powder **5** (0.49 g, 81% yield). ^1^H NMR (500 MHz, CDCl3) δ 9.66 (s, 1H), 7.56 (d, J 7.8 Hz, 2H), 7.52 (dd, J 11.3, 4.1 Hz, 1H), 7.48 – 7.44 (m, 1H), 7.39 (dd, J 11.0, 3.9 Hz, 2H), 7.34 (d, J 7.9 Hz, 1H), 7.22 (dd, J 7.9, 4.2 Hz, 1H), 7.19 – 7.14 (m, 1H), 4.36 (s, 2H). ^13^C NMR (126 MHz, CDCl3) δ 172.33 (s), 171.13 (s), 139.47 (s), 138.82 (s), 131.71 (s), 131.36 (s), 130.30 (s), 129.70 (s), 128.18 (s), 127.26 (s), 123.33 (s), 121.21 (s), 56.71 (s). HRMS (ESI/ITTOF) *m/z*: [M + H]^+^Calcd for C_15_H_12_N_2_O 237.1022; found 237.1007.

### 5-(2-Chlorophenyl)-1,3-dihydro-2*H*-benzo[*e*][1,4]diazepin-2-one (3)


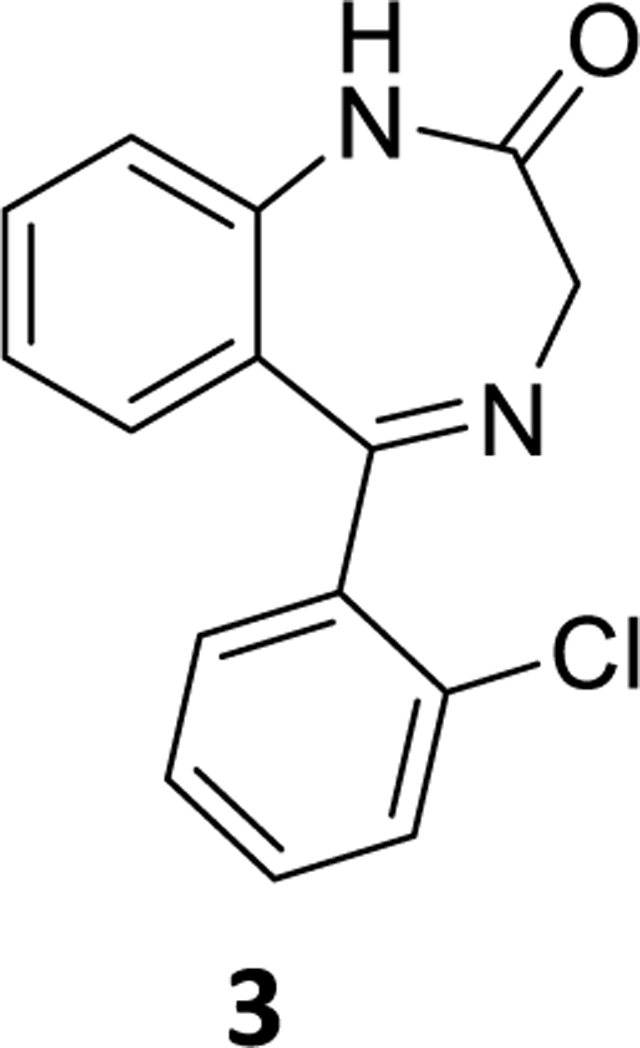


7-Bromo-5-(2-chlorophenyl)-1,3-dihydro-2H-benzo[e][1,4]diazepin-2-one (**1**) (0.8 g, 2.28 mmol) was dissolved in THF (10 mL) under argon and then Pd(OAc)_2_ (0.051 g, 0.114 mmol), and an aq potassium fluoride ( 0.264 g, 4.56 mmol in 2 mL water) solution was added under a positive flow of argon and this was followed by dropwise addition of PMHS (0.14 mL, 2.39 mmol). The reaction mixture, which resulted, was stirred the appropriate time (see Table 2) at rt and the flask was opened to the air to form more black palladium oxide and stirred with alumina, and then filtered through celite. The aq layer was extracted with ethyl acetate (3×10 mL); and the combined organic layer was washed with aq 10% sodium chloride solution (2×10 mL). It was then dried (Na_2_SO_4_) and concentrated under reduced pressure. The residue was purified by silica gelchromatography using EtOAc: hexane (40:60). The desired fractions were collected, evaporated and dried under vacuum to obtain a white powder **3** (0.51 g, 75% yield). ^1^H NMR (500 MHz, CDCl3) δ 9.39 (s, 1H), 7.55 (ddd, J 7.6, 4.7, 2.3 Hz, 1H), 7.49 (ddd, J 8.7, 6.1, 2.8 Hz, 1H), 7.41 – 7.37 (m, 3H), 7.19 (d, J 8.3 Hz, 1H), 7.11 (ddd, 2H), 4.42 (s, 2H).^13^C NMR (126 MHz, CDCl3) δ 171.57 (s), 170.57 (s), 139.10 (s), 137.96 (s), 133.27 (s), 131.85 (s), 131.03 (s), 130.63 (s), 130.03 (s), 129.90 (s), 127.91 (s), 126.84 (s), 123.85 (s), 121.04 (s), 56.59 (s). HRMS (ESI/ITTOF) *m/z*: [M + H]^+^Calcd for C_15_H_11_N_2_OCl 271.0633; found 271.0608.

### (*R*)-7-Bromo-3-methyl-5-phenyl-1,3-dihydro-2*H*-benzo[*e*][1,4]diazepin-2-one (5b)


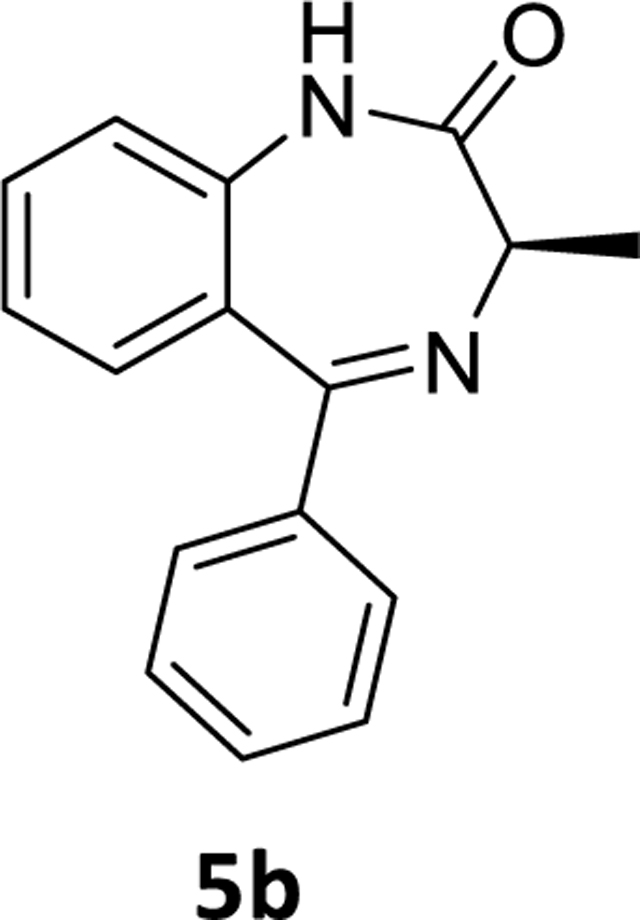


The (*R*)-7-bromo-3-methyl-5-phenyl-1,3-dihydro-2*H*-benzo[*e*][1,4]diazepin-2-one **4b** (1g, 3.03 mmol), tetrahydrofuran (10 mL) and Pd(OAc)_2_ (0.04 g, 0.15 mmol) were charged to a round bottom flask under argon. Then aq potassium fluoride (0.351g, 6 mmol in 2 mL water) solution and PMHS (0.16 mL, 2.65 mmol) were added to the mixture and it was stirred for the time mentioned in Table 2. The flask was then opened to the air, stirred with alumina, and filtered through celite. The aq layer was extracted with ethyl acetate (3×10 mL) and the combined organic layer was washed with aq 10% sodium chloride solution (2×10mL), dried over Na_2_SO_4_ and concentrated under reduced pressure. The residue, which resulted, was purified with silica gel chromatography using EtOAc: hexane (35:65). The appropriate fractions were pooled, and the solvents were removed under reduced pressure. The residue was then dried under vacuum to obtain a white powder **5b** (0.55g, 73%). ^1^H NMR (500 MHz, CDCl3) δ 9.40 (s, 1H), 7.55 – 7.50 (m, 3H), 7.43 (ddd, 1H), 7.39 – 7.32 (m, 3H), 7.22 (d, J 8.2 Hz, 1H), 7.15 (t, J 7.6 Hz, 1H), 3.79 (q, J 6.5 Hz, 1H), 1.79 (d, J 6.5 Hz, 3H). ^13^C NMR (126 MHz, CDCl3) δ 173.04 (s), 169.09 (s), 139.35 (s), 138.50 (s), 131.58 (s), 131.06 (s), 130.13 (s), 129.80 (s, 2C), 128.15 (s, 2C), 127.73 (s), 123.19 (s), 121.15 (s), 58.69 (s), 17.09 (s). HRMS (ESI/ITTOF) *m/z*: [M + H]^+^Calcd for C_16_H_14_N_2_O 251.1179; found 251.1155.

### (*S*)-3-Methyl-5-phenyl-1,3-dihydro-2*H*-benzo[*e*][1,4]diazepin-2-one(5c)


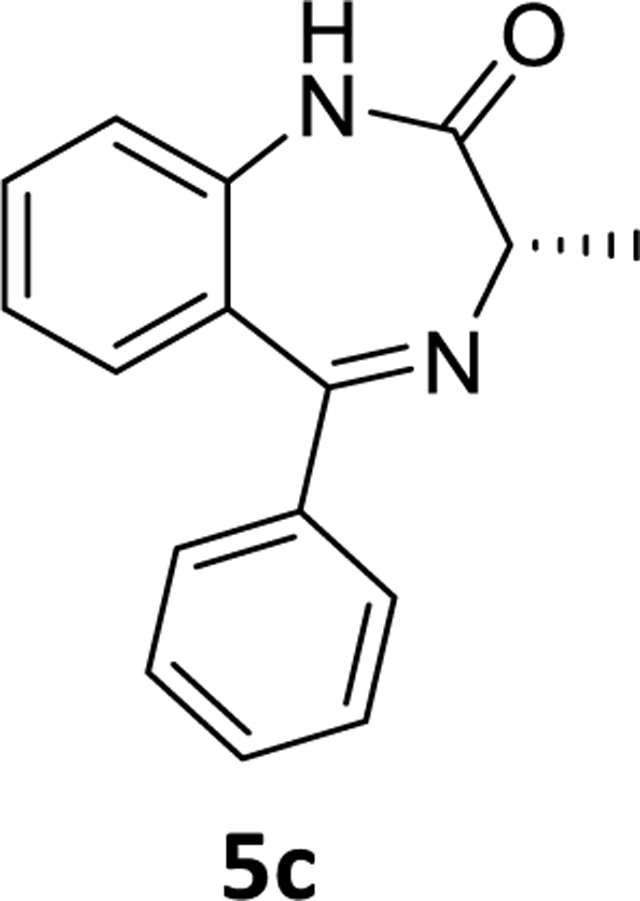


The desbromochiral compound **5c** (0.58g, 78%) was synthesized according to the procedure employed for **5b** maintaining the same scale. The yield was 78%.^1^H NMR (500 MHz, CDCl_3_) δ 9.74 (s, 1H), 7.53 (ddd, 2H), 7.50 (d, J 7.4 Hz, 1H), 7.42 (t, J 6.9 Hz, 1H), 7.38 – 7.31 (m, 3H), 7.24 (ddd, 1H), 7.14 (t, J 7.5 Hz, 1H), 3.79 (q, J 6.4 Hz, 1H), 1.80 (d, J 6.4 Hz, 3H). ^13^C NMR (126 MHz, CDCl_3_) δ 173.16 (s), 169.13 (s), 139.36 (s), 138.58 (s), 131.57 (s), 131.03 (s), 130.12 (s), 129.82 (s), 128.14 (s), 127.70 (s), 123.15 (s), 121.24 (s), 58.71 (s), 17.10 (s). HRMS (ESI/ITTOF) *m/z*: [M + H]^+^Calcd for C_16_H_14_N_2_O 251.1179; found 251.1155.

### (*S*)-5-(2-Fluorophenyl)-3-methyl-1,3-dihydro-2*H*-benzo[*e*][1,4]diazepin-2-one (5e)


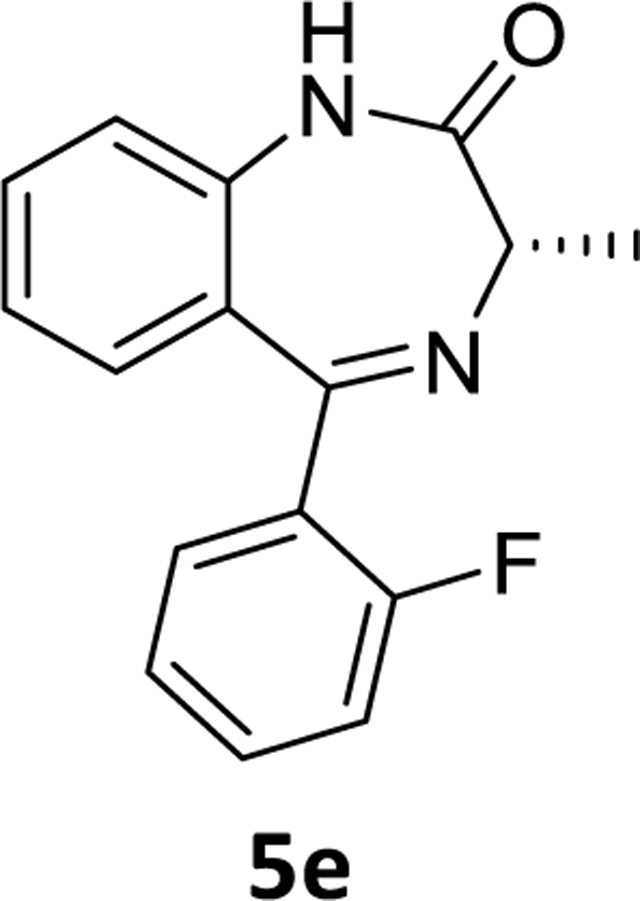


The Pd(OAc)_2_ (0.064 g, 0.144 mmol) was added to a stirred solution of (*S*)-7-bromo-5-(2-fluorophenyl)-3-methyl-1,3-dihydro-2*H*-benzo[*e*][1,4]diazepin-2-one **4e** (1 g, 2.88 mmol) in THF (10 mL) under argon. This was followed by addition of aq potassium fluoride ( 0.334 g, 5.76 mmol in 2 mL water) solution and PMHS (0.51 mL, 8.64 mmol). At the end of the reaction (TLC), the flask was opened to the air, stirred with alumina, and filtered through a pad of celite. The mixture was diluted with water and the aq layer was extracted with ethyl acetate (2×10 mL). The combined organic layer was washed with aq 10% sodium chloride solution (2×10 mL), dried (Na_2_SO_4)_ and concentrated under reduced pressure. The residue was purified with silica gel chromatography using EtOAc: hexane (35:65). The appropriate fractions were pooled, and the solvents were removed under reduced pressure. The solid, which remained, was dried under vacuum to obtain a white powder **5e** (0.61 g, 79% yield).^1^H NMR (500 MHz, CDCl3) δ 8.91 (s, 1H), 7.65 – 7.56 (m, 1H), 7.51 (t, J 7.7 Hz, 1H), 7.44 (ddd, 1H), 7.25 (dd, J 12.3, 7.7 Hz, 2H), 7.20 – 7.16 (m, 1H), 7.14 (t, J 7.6 Hz, 1H), 7.05 (dd, J 9.6, 8.9 Hz, 1H), 3.81 (q, J 6.5 Hz, 1H), 1.79 (d, J 6.5 Hz, 3H). ^13^C NMR (126 MHz, CDCl3) δ 172.26 (s), 165.77 (s), 160.49 (d, J 251.5 Hz), 137.27 (s), 131.78 (s), 131.73 (s), 131.59 (d, J 2.0 Hz), 129.77 (s), 128.59 (s), 127.85 (d, J 12.5 Hz), 124.28 (d, J 3.4 Hz), 123.73 (s), 121.03 (d, J 1.4 Hz), 116.08 (d, J 21.7 Hz), 58.72 (s), 17.01 (s). HRMS (ESI/ITTOF) *m/z*: [M + H]^+^Calcd for C_16_H_13_N_2_OF 269.1085; found 269.1062.

### (*R*)-5-(2-Fluorophenyl)-3-methyl-1,3-dihydro-2*H*-benzo[*e*][1,4]diazepin-2-one(5d)


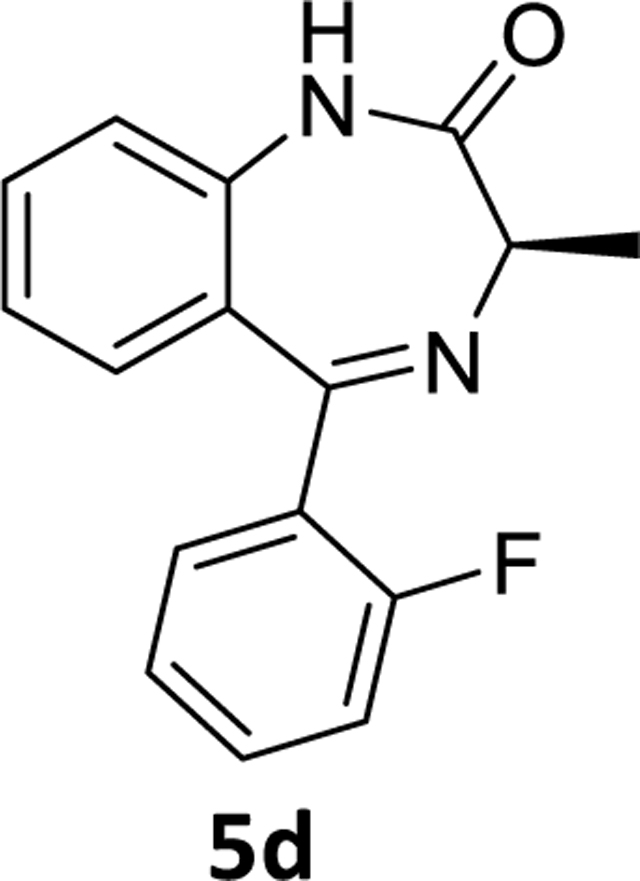


The fluoro analog **5d** (0.6 g, 78%) was synthesized, according to the procedure employed for the synthesis of **5e** maintaining the identical scale. This process took a longer reaction time since less PMHS was employed. ^1^H NMR (500 MHz, CDCl3) δ 9.15 (s, 1H), 7.60 (td, J 7.5, 1.3 Hz, 1H), 7.50 (ddd, 1H), 7.43 (ddt, 1H), 7.25 (t, J 7.3 Hz, 2H), 7.22 – 7.17 (m, 1H), 7.13 (t, J 7.6 Hz, 1H), 7.08 – 7.01 (m, 1H), 3.82 (q, J 6.5 Hz, 1H), 1.80 (d, J 6.5 Hz, 3H). ^13^C NMR (126 MHz, CDCl3) δ 172.34 (s), 165.77 (s), 160.48 (d, J 251.6 Hz), 137.32 (s), 131.72 (s), 131.68 (s), 131.60 (d, J 2.4 Hz), 129.78 (s), 128.55 (s), 127.86 (d, J 12.5 Hz), 124.24 (d, J 3.6 Hz), 123.68 (s), 121.05 (s), 116.08 (d, J 21.6 Hz), 58.73 (s), 17.03 (s). HRMS (ESI/ITTOF) *m/z*: [M + H]+ Calcd for C16H13N2OF 269.1085; found 269.1062.

### (*S*)-5-(2-Chlorophenyl)-3-methyl-1,3-dihydro-2*H*-benzo[*e*][1,4]diazepin-2-one (5f)


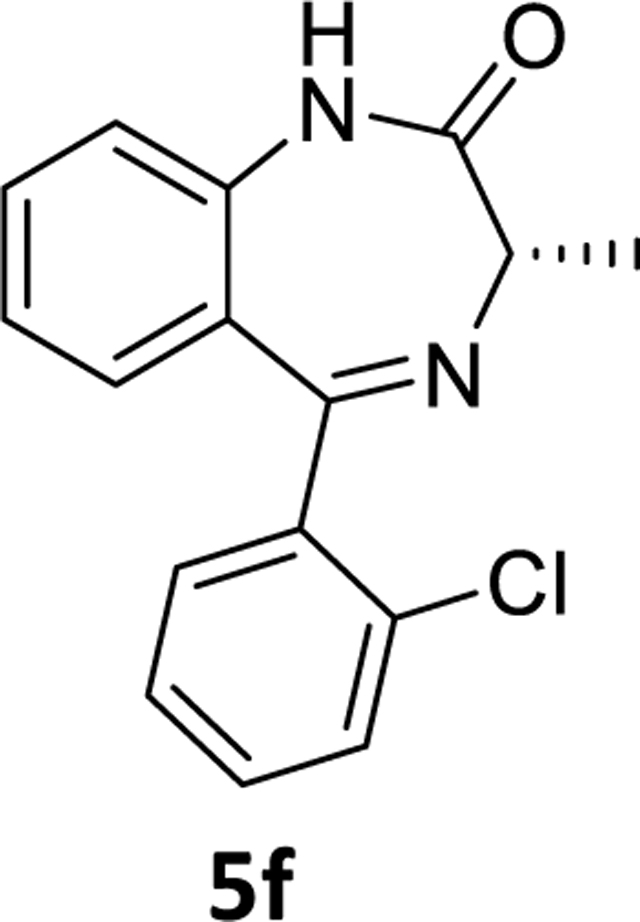


(*S)*-7-Bromo-5-(2-chlorophenyl)-3-methyl-1,3-dihydro-2*H*-benzo[*e*][1,4]diazepin-2-one **4f** (0.9g, 2.47 mmol), THF (10 mL)was charged to a round bottom flask,after which,Pd(OAc)_2_ (0.027 g, 0.123 mmol) was added to the stirred solution under a positive flow of argon. Then aq potassium fluoride (0.287g, 4.94 mmol in 2 mL water) solution and PMHS (0.16 mL, 2.65 mmol) were added, after which, the mixture was stirred for the time depicted in Table 2. The flask was then opened to the air upon the consumption of starting material. It was then stirred with alumina and filtered through a pad of celite. The organic layer was diluted with water and the aq layer was extracted with ethyl acetate (3×10 mL). The combined organic layer was washed with aq 10% sodium chloride solution (2×10 mL), dried (Na_2_SO_4_)and then concentrated under reduced pressure. The residue was purified by silica gel chromatography using EtOAc: hexane (40:60). The desired fractions were collectedand the solvent was evaporated under reduced pressure. The solid was dried under vacuum to obtain a light yellow powder **5f** (0.52g, 75%).^1^H NMR (500 MHz, CDCl3) δ 8.98 (s, 1H), 7.53 (s, 1H), 7.50 (td, 1H), 7.37 (d, J 3.0 Hz, 3H), 7.17 (t, J 7.6 Hz, 1H), 7.12 (dd, 2H), 3.86 (q, J 6.5 Hz, 1H), 1.79 (d, J 6.5 Hz, 3H). ^13^C NMR (126 MHz, CDCl3) δ 168.52 (s), 138.99 (s), 137.64 (s), 133.37 (s), 131.77 (s), 131.13 (s), 130.54 (s), 130.02 (s), 129.68 (s), 128.32 (s), 128.19 (s), 126.86 (s), 123.76 (s), 120.90 (s), 58.63 (s), 16.96 (s). HRMS (ESI/ITTOF) *m/z*: [M + H]+ Calcd for C16H13N2OCl 285.0789; found 285.0767.

### (*S*)-3-Methyl-5-(pyridin-2-yl)-1,3-dihydro-2*H*-benzo[*e*][1,4]diazepin-2-one(5g)


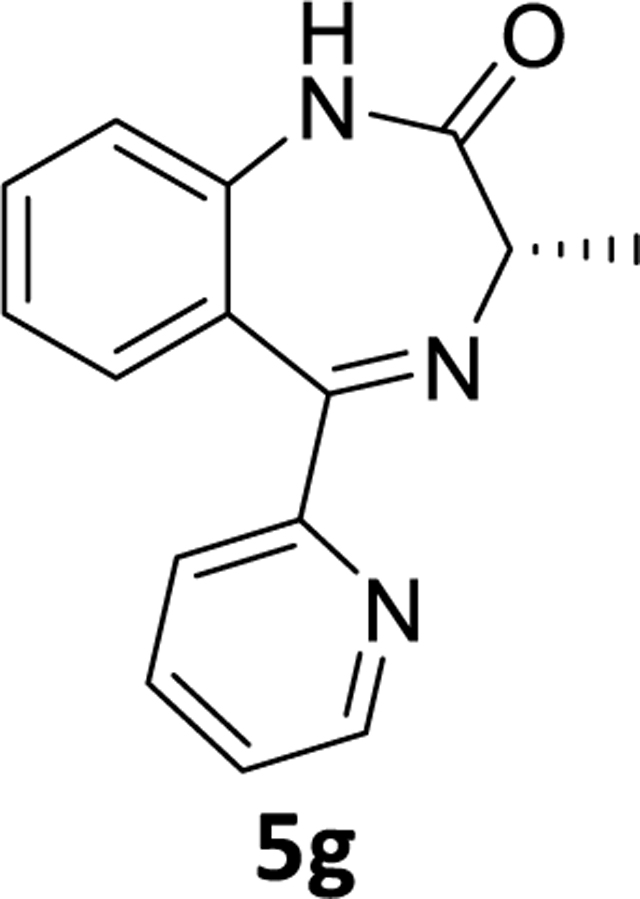


To a stirred solution of (*S*)-7-bromo-5-(2-chlorophenyl)-3-methyl-1,3-dihydro-2*H*-benzo[*e*][1,4]diazepin-2-one **4g** (1 g, 3 mmol) in THF (10 mL), Pd(OAc)_2_ (0.033 g, 0.15 mmol) was added under a positive flow of argon.This was followed by addition of aq potassium fluoride (0.348 g, 6 mmol in 3 mL water) solution and PMHS (0.19 mL, 3.15 mmol) and the reaction mixture was stirred for the appropriate time (Table 2). The flask was opened to the air and upon the consumption of starting material, it was stirred with alumina, and filtered through a pad of celite. The organic layer was diluted with water and the aq layer was extracted with ethyl acetate (3×10mL). The combined organic layer was washed with aq 10% sodium chloride solution (2×10 mL), dried (Na_2_SO_4_) and concentrated under reduced pressure. The residue was purified by silica gel chromatography using EtOAc: hexane (40:60). The expected fractions were pooled, the solvent was evaporated under reduced pressure,and dried under vacuum to obtain a white powder **5g** (0.62g, 82%). ^1^H NMR (500 MHz, CDCl_3_) δ 8.64 (dt, 1H), 8.24 (s, 1H), 8.03 (d, J 7.9 Hz, 1H), 7.82 (td, J 7.7, 1.8 Hz, 1H), 7.51 (t, J 7.8 Hz, 1H), 7.41 – 7.36 (m, 2H), 7.20 (t, J 7.6 Hz, 1H), 7.10 (dd, J 8.2, 0.8 Hz, 1H), 3.86 (q, J 6.5 Hz, 1H), 1.79 (d, J 6.5 Hz, 3H). ^13^C NMR (126 MHz, CDCl_3_) δ 172.09 (s), 167.82 (s), 156.78 (s), 148.88 (s), 138.18 (s), 136.72 (s), 131.72 (s), 131.43 (s), 126.88 (s), 124.40 (s), 124.22 (s), 123.29 (s), 121.00 (s), 58.93 (s), 17.00 (s). HRMS (ESI/ITTOF) *m/z*: [M + H]+ Calcd for C_16_H_13_N_3_O 252.1131; found 252.1113.

### 5-(2-Fluorophenyl)-2-oxo-2,3-dihydro-1*H*-benzo[*e*][1,4]diazepin-3-yl acetate(5h)


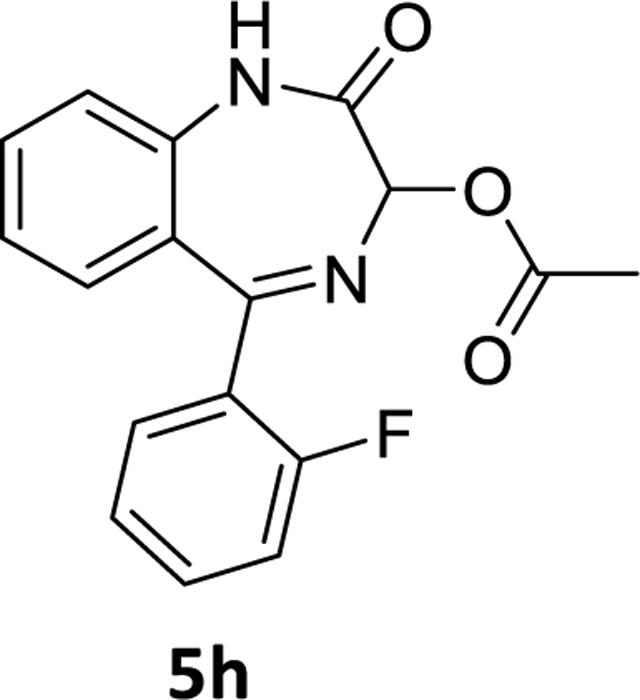


A round bottom flask was charged with 7-bromo-5-(2-fluorophenyl)-2-oxo-2,3-dihydro-1*H*-benzo[*e*][1,4]diazepin-3-yl acetate **4h** (1g, 2.5 mmol), THF (10 mL), and the Pd(OAc)_2_ (0.028 g, 0.125 mmol) was added to the stirred solution under a positive flow of argon. Then aq potassium fluoride (0.291g, 5 mmol in 2mL water) solution and PMHS (0.16 mL, 2.65 mmol) were added.The mixture, which resulted, was stirred for the time mentioned in Table 2. The flask was opened to the air upon the consumption of starting material, and stirred with alumina. It was then filtered through a pad of celite. The organic layer was diluted with water and the aq layer was extracted with ethyl acetate (3×10 mL). The combined organic layers were washed with an aq 10% sodium chloride solution (2×10 mL), dried (Na_2_SO_4_),and concentrated under reduced pressure. The residue was purified by silica gel chromatography using EtOAc: hexane (40:60). The expected fractions were collected, evaporated, and dried under vacuum to obtain a light yellow powder **5h** (0.58g, 73%). ^1^H NMR (500 MHz, CDCl3) δ 8.92 (s, 1H), 7.68 (td, J 7.5, 1.4 Hz, 1H), 7.56 (t, J 7.7 Hz, 1H), 7.51 – 7.46 (m, 1H), 7.29 (t, J 5.2 Hz, 2H), 7.25 – 7.17 (m, 2H), 7.08 (t, 1H), 6.04 (s, 1H), 2.36 (s, 3H). ^13^C NMR (126 MHz, CDCl3) δ 170.40 (s), 164.11 (s), 160.56 (d, J 252.6 Hz), 136.35 (s), 132.60 (s), 132.51 (s), 132.44 (s), 131.84 (d, J 1.6 Hz), 129.82 (s), 128.29 (s), 126.74 (d, J 12.0 Hz), 124.47 (s), 124.37 (d, J 3.6 Hz), 121.72 (s), 116.17 (d, J 21.5 Hz), 84.54 (s), 21.20 (s). ). HRMS (ESI/ITTOF) *m/z*: [M + H]+ Calcd for C_17_H_13_N_2_O_3_F 313.0983; found 313.0964.

### (2-Aminophenyl)(phenyl)methanone (7a)


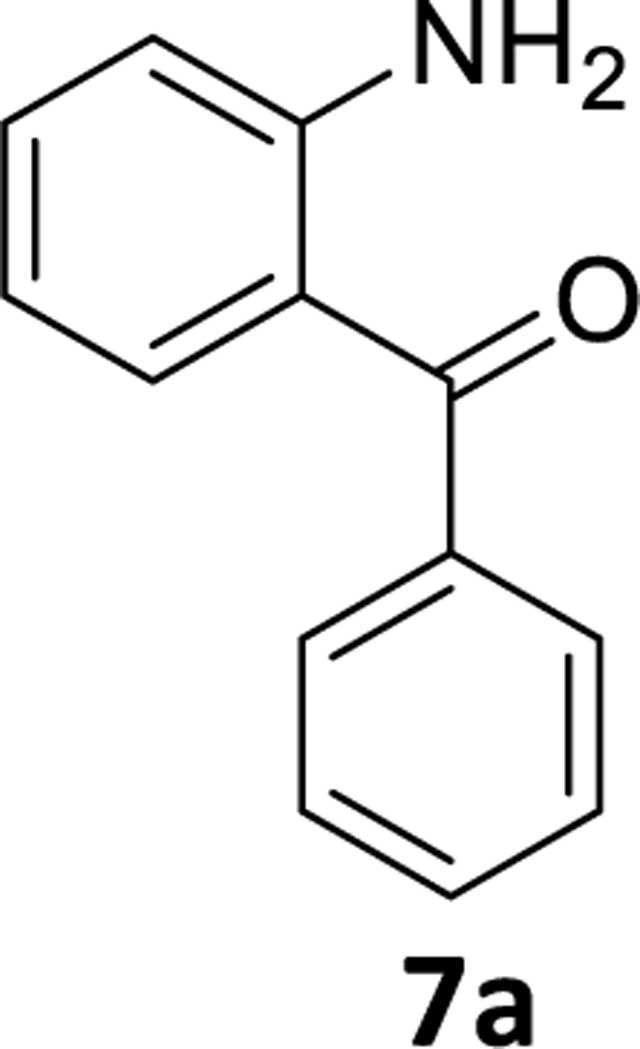


To a solution of (2-amino-5-bromophenyl)(phenyl)methanone **6a**, ( 1g, 3.62 mmol) in THF (10 mL), Pd(OAc)_2_ (0.04 g, 0.18 mmol) and aq potassium fluoride( 0.42g, 7.25 mmol in 3mL water) solution were added under a positive flow of argon. Then PMHS (0.8 mL, 14.4 mmol) was added dropwise and the mixture was then stirred for the required time (see Table 3). At the end of the reaction the flask was open to the air; then the reaction mixture was stirred with alumina and filtered through celite. The aq layer was extracted with ethyl acetate (2× 10 mL); and the combined organic layer was washed with an aq 10 % NaCl solution (3×10 mL). The organic layer was dried (Na_2_SO_4_) and concentrated under reduced pressure. The gummy greenish mass, which resulted, was then purified on silica gel (25g) chromatography using EtOAc: hexane (10:90). The appropriate fractions were collected, and the solvents were removed under reduced pressure.Then the residue was dried under vacuum at 35–40°C for 2–3 hours to provide a white powder **7a** (0.63g, 88% yield). ^1^H NMR (500 MHz, CDCl3) 1H NMR (500 MHz, CDCl3) δ 7.67 (d, J 7.3 Hz, 2H), 7.55 (t, J 7.3 Hz, 1H), 7.48 (t, J 6.7 Hz, 3H), 7.32 (t, J 7.5 Hz, 1H), 6.77 (d, J 8.2 Hz, 1H), 6.63 (t, J 7.5 Hz, 1H), 6.10 (s, 2H). ^13^C NMR (126 MHz, CDCl3) δ 199.09 (s), 150.86 (s), 140.11 (s), 134.59 (s), 134.24 (s), 131.06 (s), 129.13 (s), 128.09 (s), 118.22 (s), 117.05 (s), 115.57 (s). HRMS (ESI/ITTOF) *m/z*: [M + H]^+^Calcd for C_13_H_11_NO 198.0913; found 198.0903.

### (2-Aminophenyl)(2-fluorophenyl)methanone (7b)


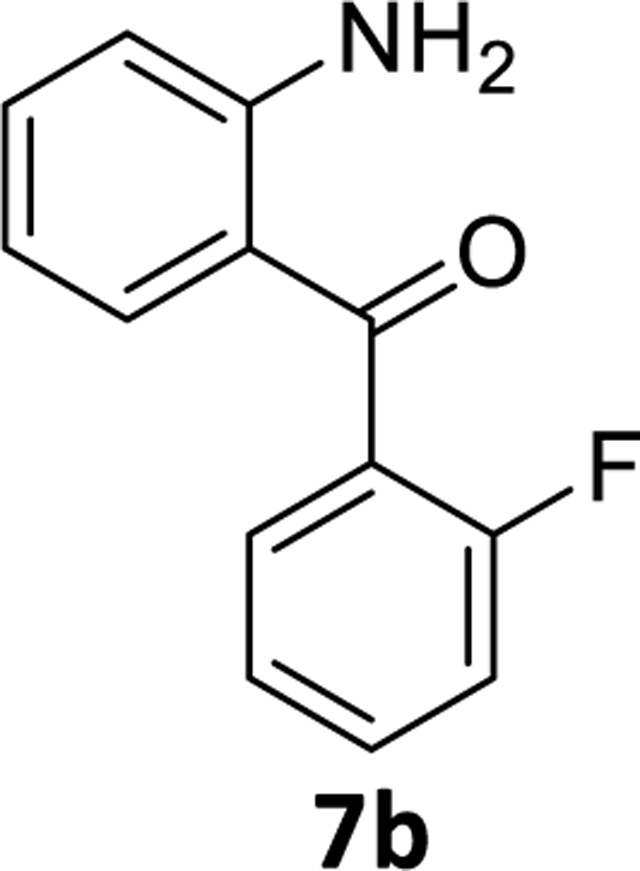


The (2-amino-5-bromophenyl)(2-fluorophenyl) methanone **6b** (1g, 3.34 mmol), and tetrahydrofuran (8 mL) were charged to a round bottom flask. To this solution Pd(OAc)_2_ (0.04 g, 0.18 mmol) was added under a positive pressure of argon. Then addition of aq. potassium fluoride (0.357g, 6.68mmol in 2mL water) solution was added to the mixture and this was followed by dropwise addition of PMHS (0.21 mL, 3.5mmol), which resulted in a greenish color. The reaction mixture was opened to the air after consumption of starting material. The reaction mixture was then stirred with alumina and filtered through celite. The aq layer was extracted with ethyl acetate (3×10 mL) and the combined organic layer was washed with aq10% NaCl (2×20 mL) solution. It was then dried (Na_2_SO_4_) and purified on a silica gel column using EtOAc: hexane (10:90). The solvents were removed and the solid residue was dried under high vacuum to afford a yellow powder **7b**(0.54g, 73% yield). ^1^H NMR (500 MHz, CDCl3) δ 7.51 – 7.46 (m, 1H), 7.44 (ddd, 1H), 7.37 – 7.30 (m, 2H), 7.26 (t, J 7.5 Hz, 1H), 7.17 (t, J 9.0 Hz, 1H), 6.74 (d, J 8.3 Hz, 1H), 6.60 (t, J 7.6 Hz, 1H), 6.41 (s, 2H). ^13^C NMR (126 MHz, CDCl3) δ 195.34 (s), 159.04 (d, ^1^J_C-F_ = 249.6 Hz), 151.15 (s), 135.07 (s), 134.55 (d, ^5^J_C-F_ = 1.6 Hz), 131.71 (d, ^3^J_C-F_ = 8.1 Hz), 129.71 (d, ^3^J _C-F_= 3.5 Hz), 128.71 (d, ^2^J_C-F_ = 16.6 Hz), 124.13 (d, ^4^J_C-F_= 3.4 Hz), 118.07 (s), 116.95 (s), 116.04 (d, ^2^J_C-F_ = 21.6 Hz), 115.78 (s). ). HRMS (ESI/ITTOF) *m/z*: [M + H]^+^Calcd for C_13_H_10_NOF 216.0819; found 216.0798.

### (2-Aminophenyl)(2-chlorophenyl)methanone (7c)


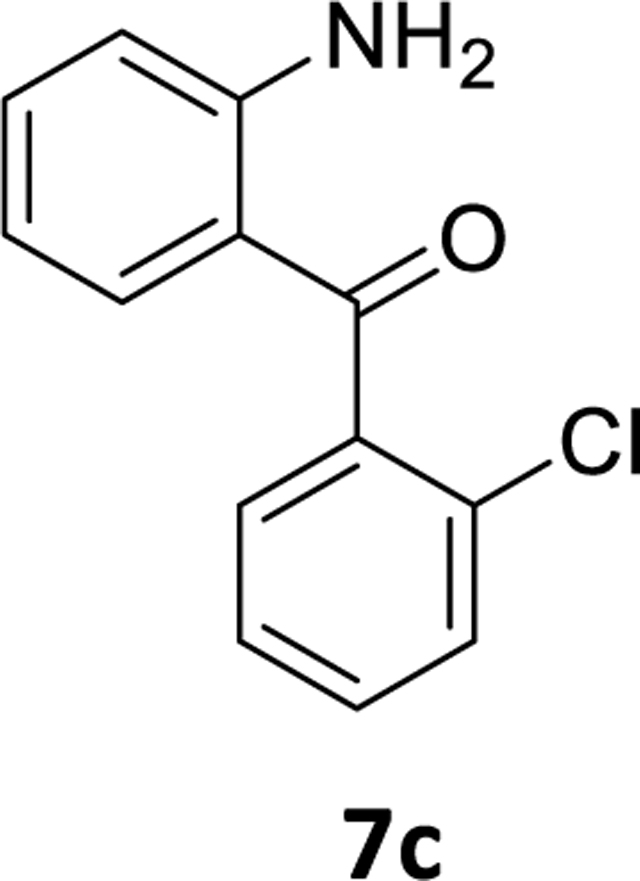


The (2-amino-5-bromophenyl)(2-chlorophenyl)methanone **6c** (1g, 3.21 mmol) was dissolved in THF (10mL) and Pd(OAc)_2_ (0.036 g, 0.161 mmol) was added under a positive pressure of argon to the solution with vigorous stirring. Then an aq potassium fluoride (0.372g, 6.42mmol in 3mL water) solution was added and this was followed by addition of PMHS (0.2mL, 3.37mmol). This process resulted in a bluish colored solution. At the end of the reaction (TLC) the flask was opened to the air, and stirred with alumina and filtered through celite. The aq layer was extracted with ethyl acetate(3×10 mL) and the combined organic layer was washed with a 10% aq NaCl solution (2×20mL). It was then dried (Na_2_SO_4_) and purified by silica gel chromatography using EtOAc: hexane (10:90). The solvents were removedunder reduced pressure and the residue was dried under high vacuum to afford a yellow powder **7c**(0.53g, 71% yield).^1^H NMR (500 MHz, CDCl_3_) δ 7.47 (dd, J 7.9, 1.0 Hz, 1H), 7.41 (td, J 7.6, 2.0 Hz, 1H), 7.37 (td, J 7.3, 1.3 Hz, 1H), 7.34 (dd, J 7.5, 1.8 Hz, 1H), 7.30 (dt, J 12.9, 3.6 Hz, 1H), 7.19 (dd, J 8.1, 1.4 Hz, 1H), 6.74 (dd, J 8.3, 0.5 Hz, 1H), 6.56 (td, 1H), 6.48 (s, 2H). ^13^C NMR (126 MHz, CDCl_3_) δ 197.28 (s), 151.42 (s), 139.85 (s), 135.23 (s), 134.68 (s), 130.71 (s), 130.33 (s), 129.85 (s), 128.42 (s), 126.63 (s), 117.43 (s), 117.00 (s), 115.78 (s). HRMS (ESI/ITTOF) *m/z*: [M + H]^+^Calcd for C_13_H_10_NOF 232.0524; found 232.0506.

### (2-Aminophenyl)(pyridin-2-yl)methanone (7d)


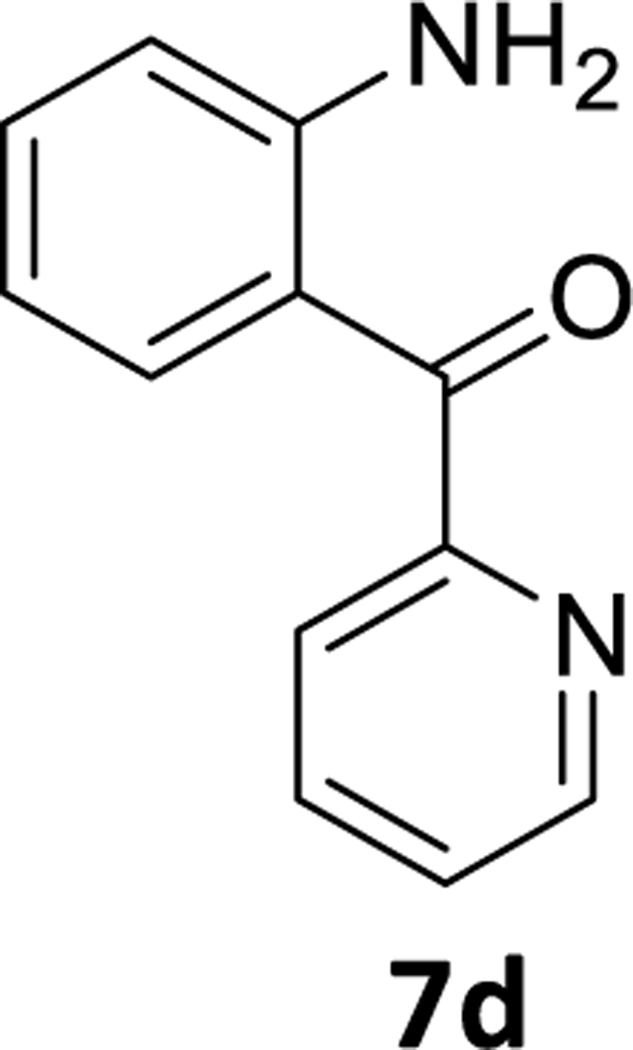


A round bottom flask was charged with (2-amino-5-bromophenyl) (pyridin-2-yl) methanone **6d**(1g, 3.6mmol), as well as THF (10mL), and the flask was purged with argon. Palladium acetate (0.04 g, 0.18 mmol) was then added under an argon atmosphere and this was followed by the addition of aq potassium fluoride (0.419g, 7.2mmol in 4mL of water) solution. Then PMHS (0.23mL, 3.78mmol) was added dropwise to the reaction mixture, which resulted in a deep greenish colored solution. The reaction mixture was then stirred for the required time(see Table 3), and the round bottom flask was opened to the air at the end of the reaction. Then 2 g of alumina was added to the reaction flask and it was stirred for 5 min. The reaction mixture was then filtered through a pad of celite.Water and ethyl acetate was added to the filtrate. The layers were separated, and the aq layer was extracted with ethyl acetate (3×10mL) and the combined organic layer was washed with an aq 10% NaCl solution (2×10mL). It was then dried (Na_2_SO_4_) and purified by silica gel chromatography using EtOAc: hexane (10:90). The solvents were removed under reduced pressure and the solid residue was dried under high vacuum to afford a yellow powder**7d** (0.50g, 69% yield).^1^H NMR (500 MHz, CDCl_3_) δ 8.70 (ddd, J 4.8, 1.7, 0.9 Hz, 1H), 7.86 (td, J 7.7, 1.7 Hz, 1H), 7.76 (dt, J 7.8, 1.1 Hz, 1H), 7.65 (dd, J 8.2, 1.5 Hz, 1H), 7.43 (ddd, J 7.6, 4.8, 1.2 Hz, 1H), 7.30 (ddd, J 8.5, 7.0, 1.6 Hz, 1H), 6.73 (dd, J 8.4, 0.8 Hz, 1H), 6.61 (ddd, J 8.1, 7.1, 1.1 Hz, 1H), 6.32 (s, 1H).^13^C NMR (126 MHz, CDCl_3_) δ 196.04 (s), 157.52 (s), 151.83 (s), 148.46 (s), 136.89 (s), 135.03 (s), 134.81 (s), 124.95 (s), 123.88 (s), 117.03 (s), 116.78 (s), 115.57 (s). ). HRMS (ESI/ITTOF) *m/z*: [M + H]^+^Calcd for C_12_H_10_N_2_O 199.0866; found 199.0844.

### Ethyl (*R*)-6-(2-fluorophenyl)-4-methyl-4*H*-benzo[*f*]imidazo[1,5-*a*][1,4]diazepine-3-carboxylate(9a)


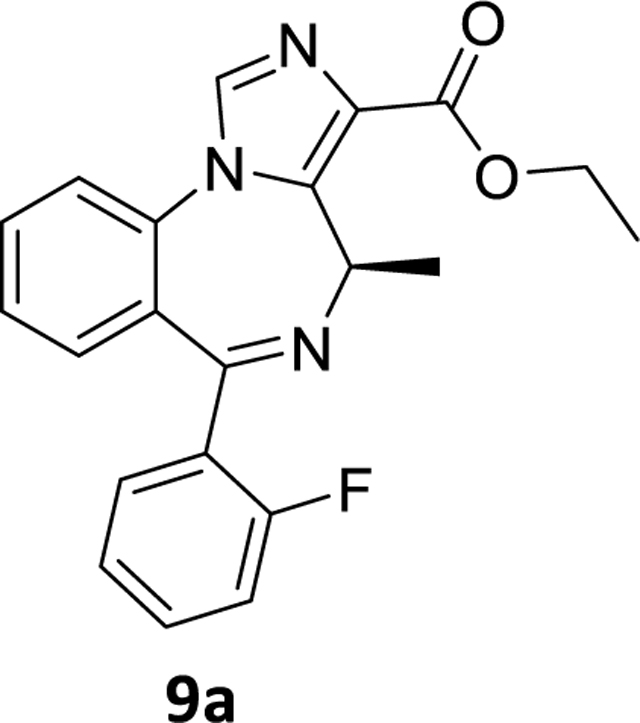


The Pd(OAc)_2_ (0.029 g, 0.13 mmol) was added under a positive flow of argon to a stirred solution of (ethyl(R)-8-bromo-6-(2-fluorophenyl)-4-methyl-4*H*-benzo[*f*]imidazo[1,5-*a*][1,4]diazepine-3 carboxylate **8a** (1g, 2.26 mmol) in THF (10 mL). This addition was followed by addition of aqpotassium fluoride (0.262g, 4.52 mmol in 3mL water) solution and PMHS (0.14 mL, 2.37 mmol). This reaction mixture was stirred for 2hr at rt, and then the flask was opened to the air upon the consumption of starting material (TLC). The mixture was stirred with alumina, and filtered through a pad of celite. The organic layer, which resulted, was diluted with water and the aq layer was extracted with ethyl acetate (3×10 mL). The combined organic layer was washed with an aq 10% sodium chloride solution (2×10 mL), dried (Na_2_SO_4_),and concentrated under reduced pressure. The residue was purified on a silica gel column using EtOAc: hexane (50:50).^1^H NMR (500 MHz, CDCl3) δ 7.93 (d, J 14.8 Hz, 1H), 7.72 (d, J 7.9 Hz, 1H), 7.60 (s, 2H), 7.47 (dd, J 11.8, 8.1 Hz, 1H), 7.44 – 7.30 (m, 2H), 7.25 (dt, J 11.6, 7.6 Hz, 1H), 7.04 (dt, J 14.7, 9.5 Hz, 1H), 6.71 (q, J 13.8, 6.8 Hz, 1H), 4.41 (q, J 15.9, 7.4 Hz, 2H), 1.42 (t, J 7.1, 1.4 Hz, 3H), 1.29 (d, J 7.5 Hz,3H). HRMS (ESI/ITTOF) *m/z*: [M + H]^+^Calcd for C_12_H_10_N_2_O 364.1456; found 364.1437.

### Adult schistosome mobility assays

*S. mansoni* cercariae (NMRI strain) infected Swiss Webster mice (female) were sacrificed after 49 days of infection by CO_2_ euthanasia. The mesenteric vasculature of mice was dissected to recover adult schistosomes. The schistosomes were harvested and washed in DMEM (ThermoFisher cat. # 11995123) supplemented with HEPES (25mM), 5% v/v heat inactivated FCS (Sigma Aldrich cat. # 12133C) and Penicillin-Streptomycin (100 units/mL). 6 well dishes (4–5 male worms in 3mL media per well) were used to culture worms in the presence of various test compounds or DMSO (vehicle control) overnight (37 °C / 5% CO_2_). Worms were imaged the following day to record movement phenotypes using a Zeiss Stemi 504 stereomicroscope and a CCD camera. 1 minute recordings were acquired at 4 frames per second and saved as a .TIFF stack, which was imported into ImageJ for analysis. All animal work was approved by IACUC committees at University of Wisconsin – Oshkosh and Madison (protocol numbers 000324–07-14–20 and V006353, respectively).

## Supplementary Material

supplementary material

## Figures and Tables

**Figure 2.**
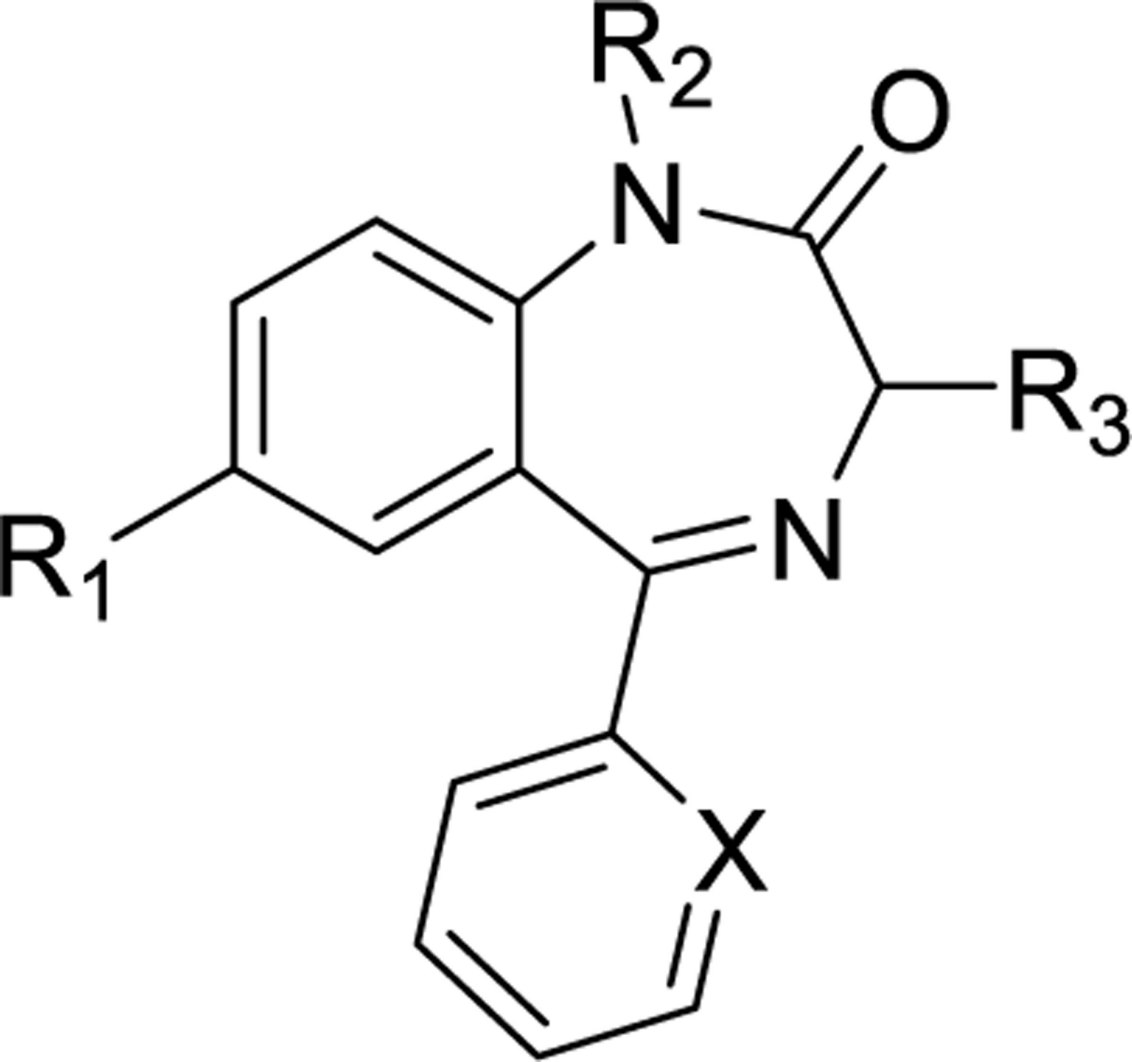
General structure of meclonazepam derivatives.

**Table 1.** Effect of different equivalents of PMHS on selective debromination

Entry	Substrate	Pd(OAc)_2_	PMHS(eq)	KF (eq)	Time	Temperature	*Ratio of Product (2:3)	^a^Yield
1	**1**	5 mol%	4	2	1 hr	r.t.	90:10	75%
2	**1**	1 mol%	4	2	2 hr	r.t.	83:17	79%
3	**1**	2 mol%	4	2	2 hr	r.t.	80:20	69%
4	**1**	3 mol%	4	2	1.5 hr	r.t.	71:29	73%
5	**1**	4 mol%	4	2	1.3 hr	r.t.	65:35	75%
6	**1**	5 mol%	3	2	2.2 hr	r.t	60:40	71%
7	**1**	5 mol%	2.5	2	2.5 hr	r.t	60:40	71%
8	**1**	5 mol%	1.05	2	3 hr	r.t	51:49^b^	65%^C^
9	**1**	5 mol%	1.05	2	4.0 hr	r.t	22:78 ^b^	84%^C^
10	**1**	5 mol%	1.05	2	6.0 hr	r.t.	5:95	79%

* From HPLC (Pinnacle -C18 (2.1 mm × 50 mm, 1.8 μm particle size) column, flow rate of 0.5 mL/min, mobile phase-acetonitrile and water (containing 0.1% formic acid));

a Isolated combined yield of (**2** and **3**);

b Ratio of **1** and **3**;

C Combined yield of (**1** and **3**)

**Table 2.** Debromination strategy on different benzodiazepines

Entry	Substrate	X	R’	PMHS (eq)	Time	Product	^a^Yield (%)
1	**4a**	C-H	H	1.05	3.5 hr	**5a**	71%
2	**4b**	C-H	CH_3_(R)	1.05	4 hr	**5b**	73%
3	**4c**	C-H	CH_3_(S)	1.05	3.5 hr	**5c**	79%
4	**4d**	C-F	CH_3_(R)	1.05	4 hr	**5d**	78%
5	**4e**	C-F	CH_3_(S)	3.0	2.0 hr	**5e**	79%
6	**4f**	C-Cl	CH_3_(S)	1.05	4.5 hr	**5f**	75%
7	**4g**	N	CH_3_(S)	1.05	2 hr	**5g**	81%
8	**4h**	C-F	OCOCH_3_	4	45 min	**5h**	73%

a Isolated yield

**Table 3.** Selective debromination of benzophenone substrate

Entry	Substrate	X	Pd(OAc)_2_	PMHS(eq)	KF (eq)	Time	Product	^a^Yield
1	**6a**	C-H	5 mol%	1.05	2	3.5 hr	**7a**	78%
2	**6b**	C-F	5 mol%	1.05	2	3.0 hr	**7b**	73%
3	**6c**	C-Cl	5 mol%	1.05	2	3.0 hr	**7c**	71%
4	**6d**	N	5 mol%	1.05	2	2.5 hr	**7d**	69%
5	**6a**	C-H	5 mol%	2	2	2.0 hr	**7a**	69%
6	**6a**	C-H	5 mol%	3	2	1.0 hr	**7a**	75%
7	**6a**	C-H	5 mol%	4	2	30 min	**7a**	70%
8	**6b**	C-F	5 mol%	4	2	45 min	**7b**	73%

a Isolated yield

**Table 4.** Anti-schistosomal effect of C-7 H benzodiazepines

SL	R_1_	R_2_	R_3_	X	Code		Activity

1	NO_2_	H	S-CH_3_	C-Cl	MCLZ	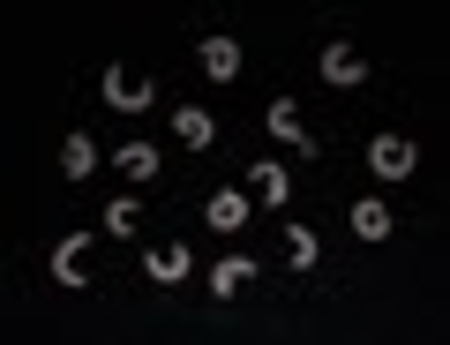	Coiled,dead IC_50_ 160nm
2	H	H	S-CH_3_	C-Cl	MYM-V-03	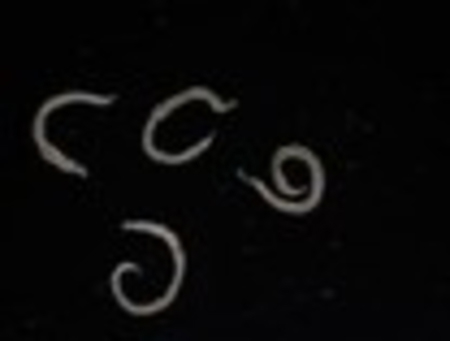	alive, 30μM
3	H	H	S-CH_3_	C-F	MYM-I-84	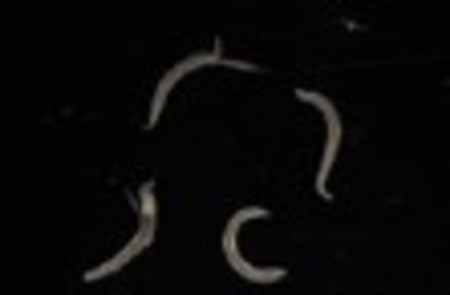	alive, 30μM
4	H	H	R-CH_3_	C-F	MYM-I-94	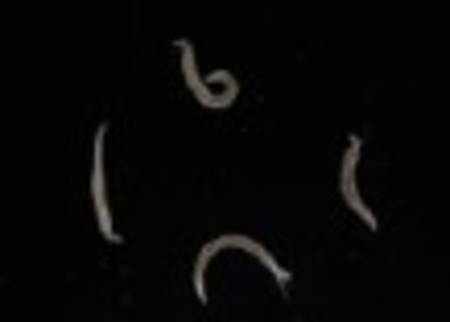	alive, 30μM
5	H	H	S-CH_3_	C-H	MYM-I-85	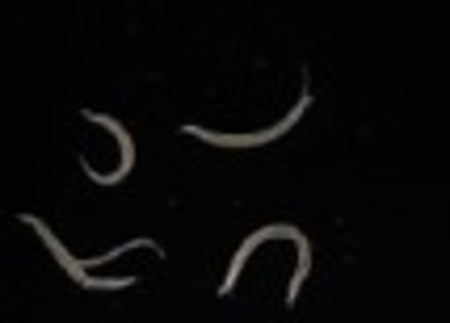	alive, 30μM
6	H	H	R-CH_3_	C-H	MYM-I-86	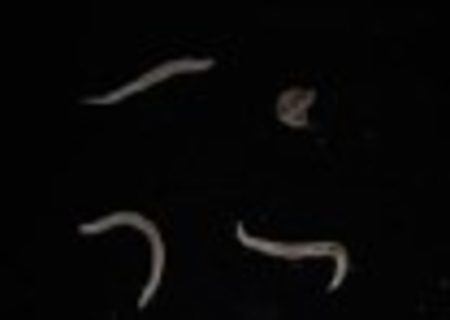	alive, 30μM
7	H	H	S-CH_2_CH_3_	C-Cl	MYM-V-18	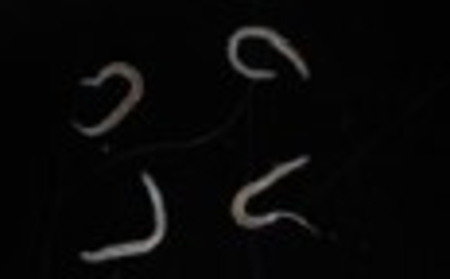	alive, 30μM
8	CN	H	S-CH_3_	C-Cl	MYM-IV-12	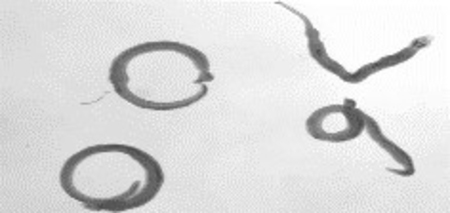	alive, 30μM
