# Supplementary material for: An efficient debromination technique using PMHS with a number of ligands containing different functional groups
Source: ARKIVOC. Author manuscript; Available in PMC 2024 May 21. (PMC11107800)
Supplement: supplementary material [file NIHMS1810590-supplement-supplementary_material.pdf]

Supplementary Material

An efficient debromination technique using PMHS with a number of ligands containing different functional groups

Md Yeunus Mian,[a] Prithu Mondal,[a] Dishary Sharmin,[a] Kamal P. Pandey,[a] Farjana Rashid,[a] Sepideh Rezvanian,[a] Lalit K. Golani,[a] V.V.N. Phani Babu Tiruveedhula,[a] Vilashini Rajaratnam[1], Shama P. Mirza[1], John D. Chan[2], Jeffrey M. Witkin[1], and James M. Cook*[1]

[1]*Department of Chemistry and Biochemistry, Milwaukee Institute of Drug Discovery, University of Wisconsin-Milwaukee, Milwaukee, WI-53211*
[2]*Department of Chemistry, University of Wisconsin-Oshkosh, Oshkosh, WI-54901*
E-mail: capncook@uwm.edu

Table of Contents

1. ^{1}HNMR spectra of compound **2** S3
2. ^{13}CNMR spectra of compound **2** S3
3. High resolution mass spectra of compound **2** S4
4. ^{1}HNMR spectra of compound **3** S5
5. ^{13}CNMR spectra of compound **3** S5
6. High resolution mass spectra of compound **3** S6
7. ^{1}HNMR spectra of compound **5b** S7
8. ^{13}CNMR spectra of compound **5b** S7
9. High resolution mass spectra of compound **5b** S8
10. ^{1}HNMR spectra of compound **5c** S9
11. ^{13}CNMR spectra of compound **5c** S9
12. High resolution mass spectra of compound **5c** S10
13. ^{1}HNMR spectra of compound **5d** S11
14. ^{13}CNMR spectra of compound **5d** S11
15. High resolution mass spectra of compound **5d** S12
16. ^{1}HNMR spectra of compound **5e** S13
17. ^{13}CNMR spectra of compound **5e** S13
18. High resolution mass spectra of compound **5e** S14
19. ^{1}HNMR spectra of compound **5f** S15
20. ^{13}CNMR spectra of compound **5f** S15
21. High resolution mass spectra of compound **5f** S16
22. ^{1}HNMR spectra of compound **5g** S17

 ©AUTHOR(S)

23. ^{13}CNMR spectra of compound **5g** S17
24. High resolution mass spectra of compound **5g** S18
25. ^{1}HNMR spectra of compound **5h** S19
26. ^{13}CNMR spectra of compound **5h** S19
27. High resolution mass spectra of compound **5h** S20
28. ^{1}HNMR spectra of compound **7a** S21
29. ^{13}CNMR spectra of compound **7a** S21
30. High resolution mass spectra of compound **7a** S22
31. ^{1}HNMR spectra of compound **7b** S23
32. ^{13}CNMR spectra of compound **7b** S23
33. High resolution mass spectra of compound **7b** S24
34. ^{1}HNMR spectra of compound **7b** S25
35. ^{13}CNMR spectra of compound **7b** S25
36. High resolution mass spectra of compound **7b** S26
37. ^{1}HNMR spectra of compound **7d** S27
38. ^{13}CNMR spectra of compound **7d** S27
39. High resolution mass spectra of compound **7d** S28
40. ^{1}HNMR spectra of compound **9a** S29
41. High resolution mass spectra of compound **9a** S30
42. Debromination on a double bond containing substrate S31

 ©AUTHOR(S)

5-phenyl-1,3-dihydro-2H-benzo[e][1,4]diazepin-2-one(2)

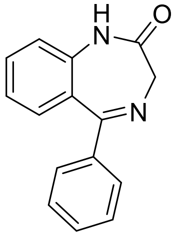

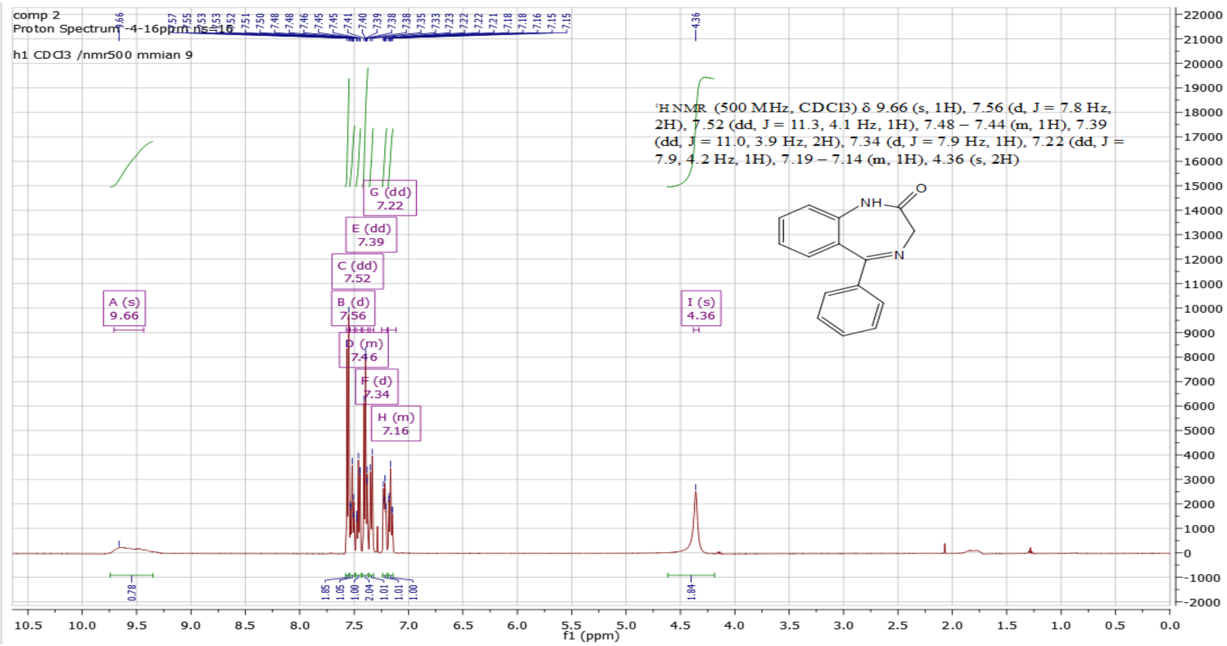

 ©AUTHOR(S)

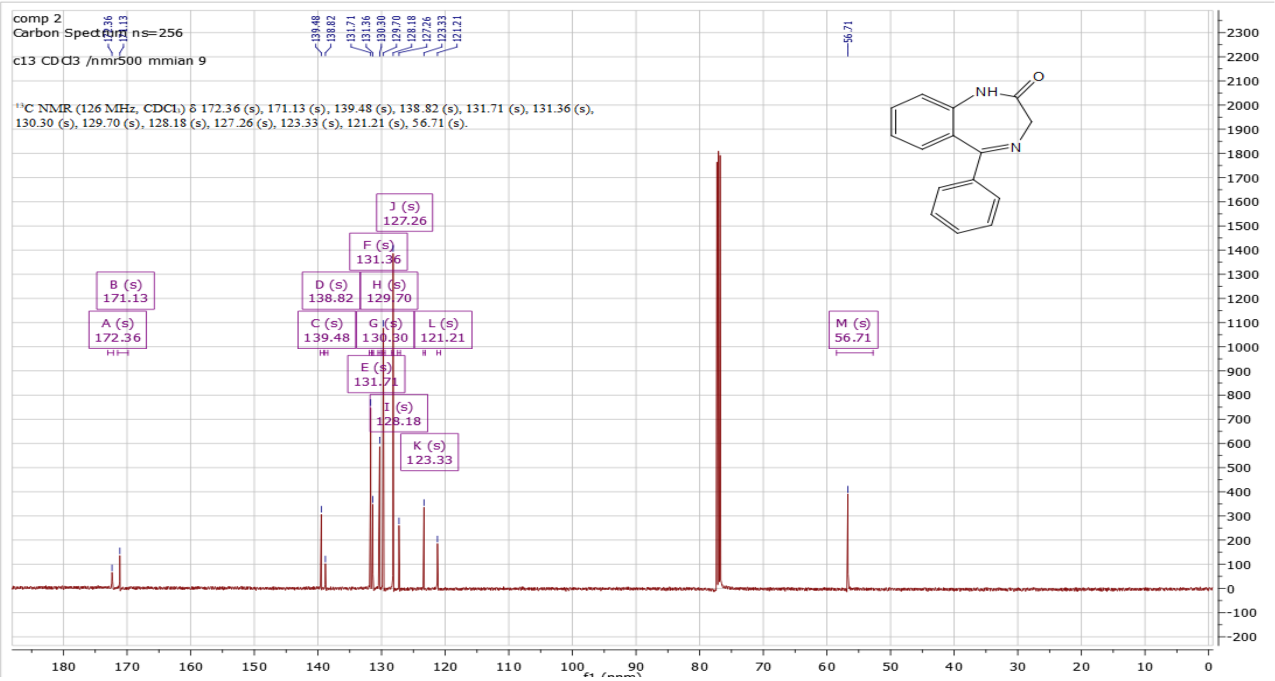
comp 2
Carbon Spectrum ns=256
c13 CDCl3 /nmr500 mmian 9
^{13}C NMR (126 MHz, CDCl$_3$) δ 172.36 (s), 171.13 (s), 139.48 (s), 138.82 (s), 131.71 (s), 131.36 (s), 130.30 (s), 129.70 (s), 128.18 (s), 127.26 (s), 123.33 (s), 121.21 (s), 56.71 (s).
NH
O
N
f1 (ppm)

Formula Predictor Report - COMP 2_12082020 Analysis_49.lcd Page 1 of 1

Data File: C:\LabSolutions\Data\Vilashini Rajaratnam\12082020 Analysis\COMP 2_12082020 Analysis_49.lcd

Elmt	Val.	Min	Max	Elmt	Val.	Min	Max	Elmt	Val.	Min	Max	Elmt	Val.	Min	Max	Use Adduct
H	1	10	15	N	3	1	3	Si	4	0	0	Br	1	0	0	H
2H	1	0	0	O	2	1	3	S	2	0	0	I	3	0	0	K
C	4	10	25	F	1	0	1	Cl	1	0	0					

Error Margin (ppm):	300	DBE Range:	-100.0 - 2000.0	Electron Ions:	both
HC Ratio:	unlimited	Apply N Rule:	no	Use MSn Info:	yes
Max Isotopes:	all	Isotope RI (%):	1.00	Isotope Res:	10000
MSn Iso RI (%):	75.00	MSn Logic Mode:	AND	Max Results:	10

Event#: 1 MS(E+) Ret. Time : 0.893 -> 0.907 - 1.147 -> 1.154 Scan# : 135 -> 137 - 173 -> 175

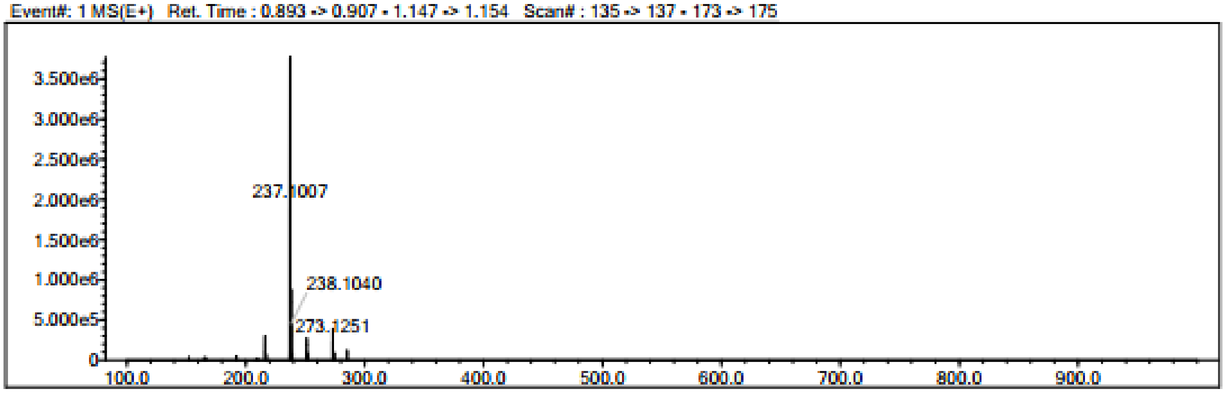

Measured region for 237.1007 m/z

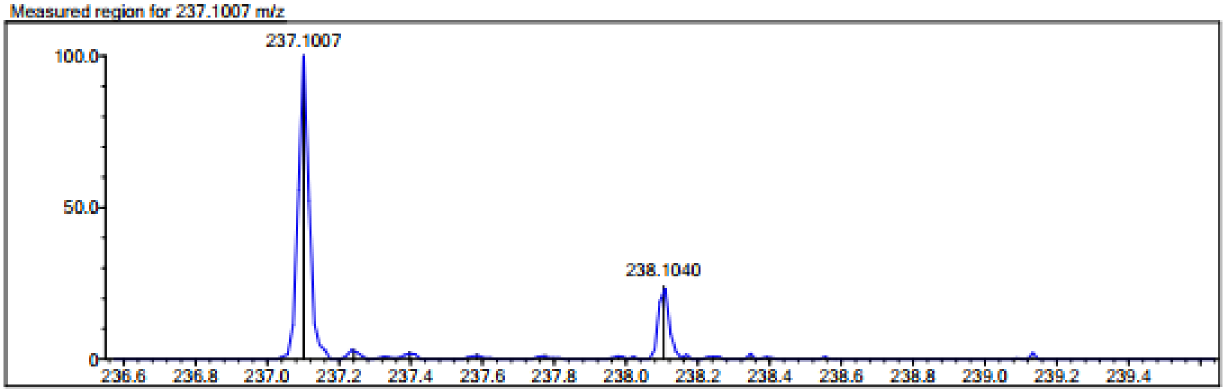

C15 H12 N2 O [M+H]+ : Predicted region for 237.1022 m/z

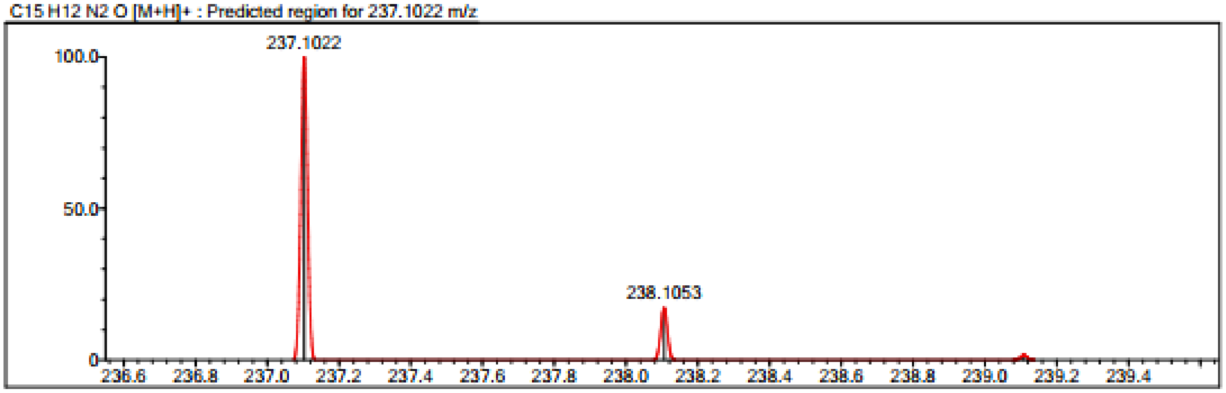

Rank	Score	Formula (M)	Ion	Meas. m/z	Pred. m/z	Df. (mDa)	Df. (ppm)	Iso	DBE
1	27.96	C15 H12 N2 O	[M+H]+	237.1007	237.1022	-1.5	-6.33	36.45	11.0

©AUTHOR(S)

5-(2-chlorophenyl)-1,3-dihydro-2H-benzo[e][1,4]diazepin-2-one(3)

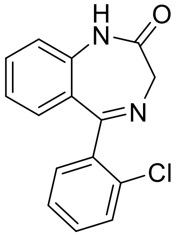

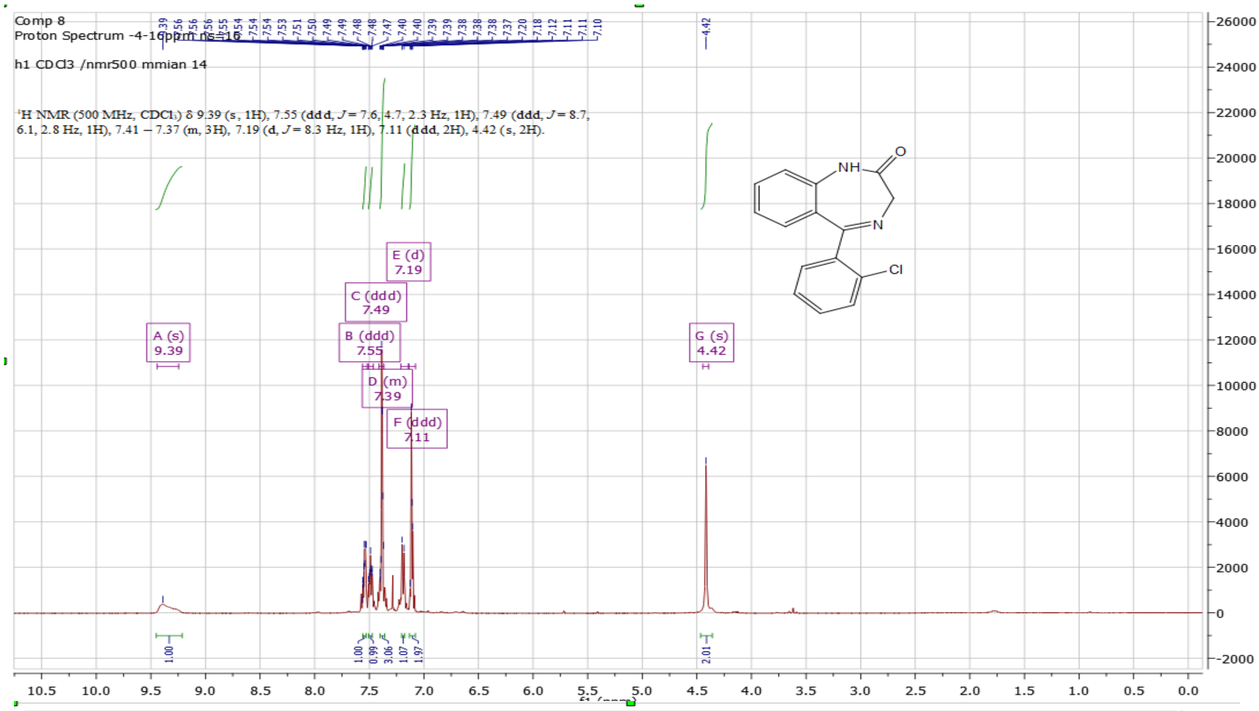

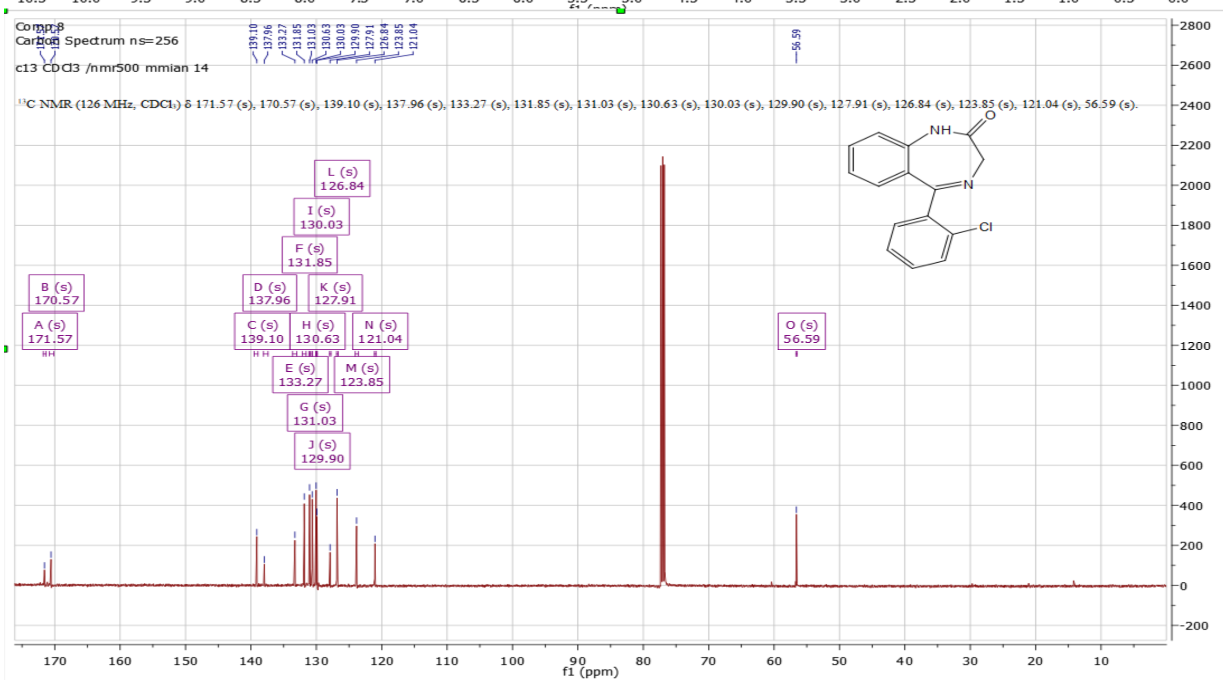

 ©AUTHOR(S)

Formula Predictor Report - COMP 8_12082020 Analysis_37.lcd Page 1 of 1

Data File: C:\LabSolutions\Data\Vilashini Rajaratnam\12082020 Analysis\COMP 8_12082020 Analysis_37.lcd

Elmt	Val.	Min	Max	Elmt	Val.	Min	Max	Elmt	Val.	Min	Max	Elmt	Val.	Min	Max	Use Adduct
H	1	10	30	N	3	1	3	Si	4	0	0	Br	1	0	0	H
2H	1	0	0	O	2	1	3	S	2	0	0	I	3	0	0	K
C	4	10	25	F	1	0	1	Cl	1	0	1					

Error Margin (ppm):	300	DBE Range:	-100.0 - 2000.0	Electron Ions:	both
HC Ratio:	unlimited	Apply N Rule:	no	Use MSn Info:	yes
Max Isotopes:	all	Isotope RI (%):	1.00	Isotope Res:	10000
MSn Iso RI (%):	75.00	MSn Logic Mode:	AND	Max Results:	10

Event#: 1 MS(E+) Ret. Time : 0.920 -> 1.053 - 0.467 -> 0.598 Scan# : 139 -> 159 - 71 -> 91

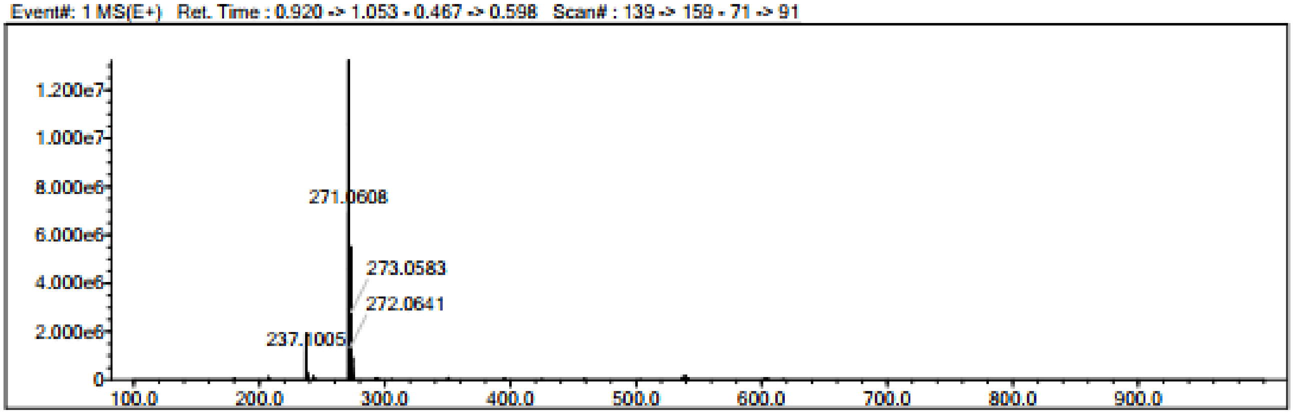

Measured region for 271.0608 m/z

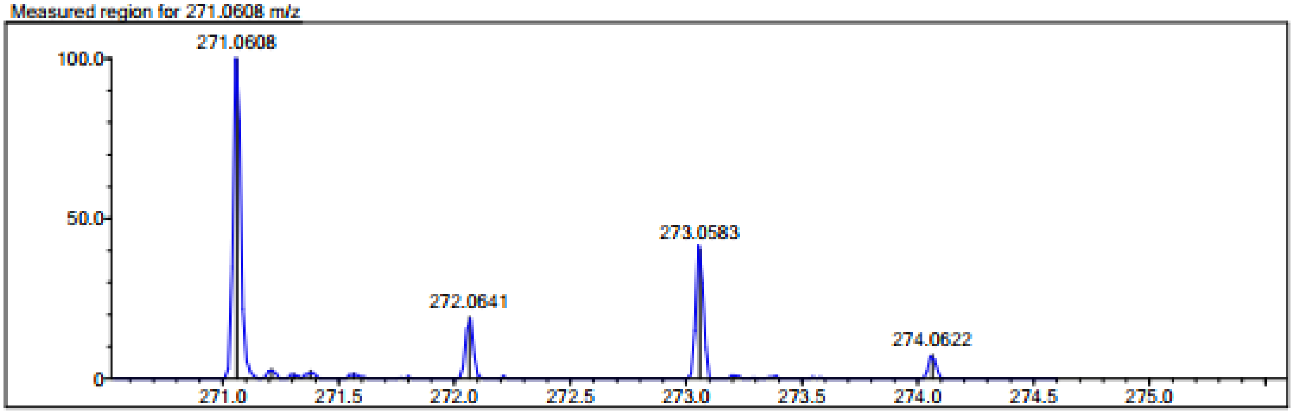

C15 H11 N2 O Cl [M+H]+ : Predicted region for 271.0633 m/z

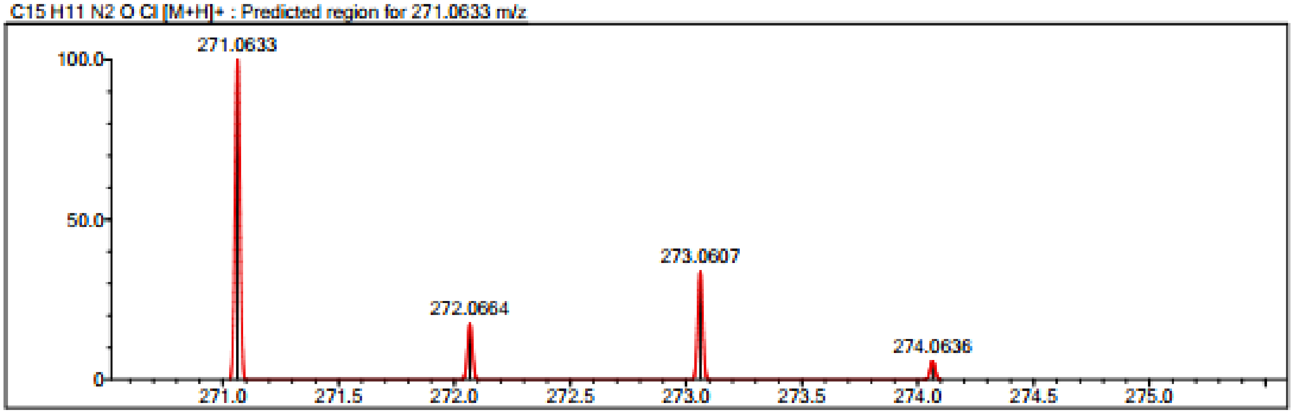

Rank	Score	Formula (M)	Ion	Meas. m/z	Pred. m/z	Df. (mDa)	Df. (ppm)	Iso	DBE
3	31.61	C15 H11 N2 O Cl	[M+H]+	271.0608	271.0633	-2.5	-9.22	66.12	11.0

 ©AUTHOR(S)

(R)-7-bromo-3-methyl-5-phenyl-1,3-dihydro-2H-benzo[e][1,4]diazepin-2-one(5b)

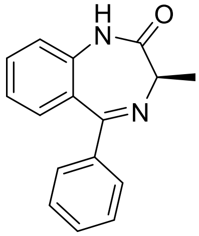

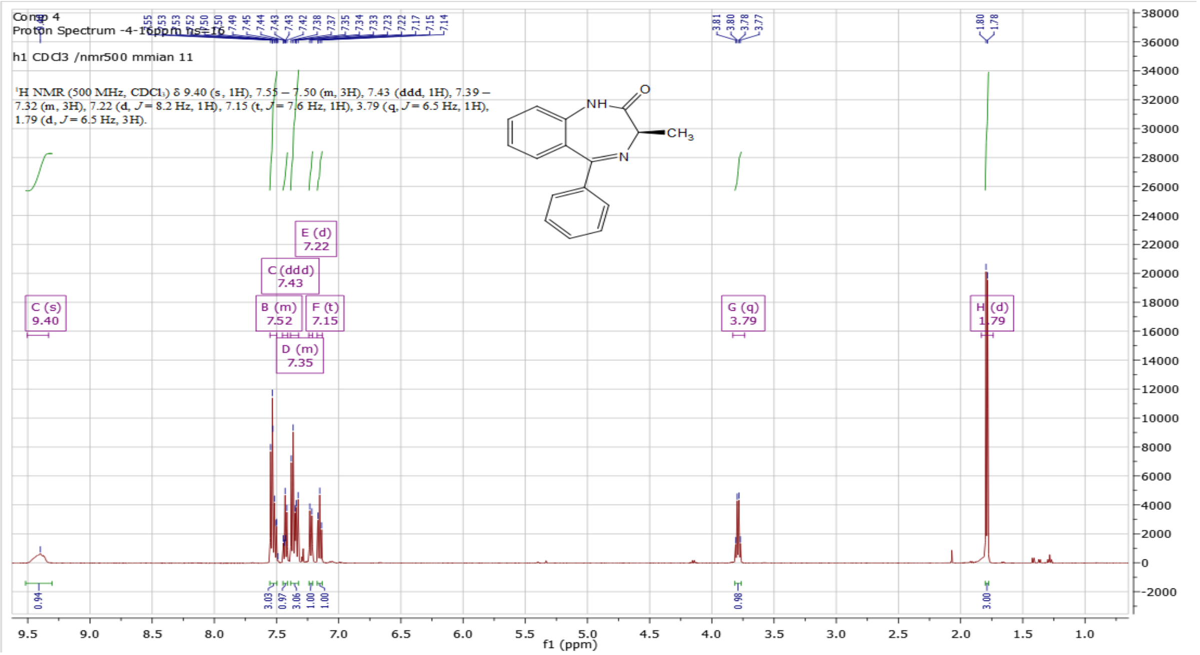

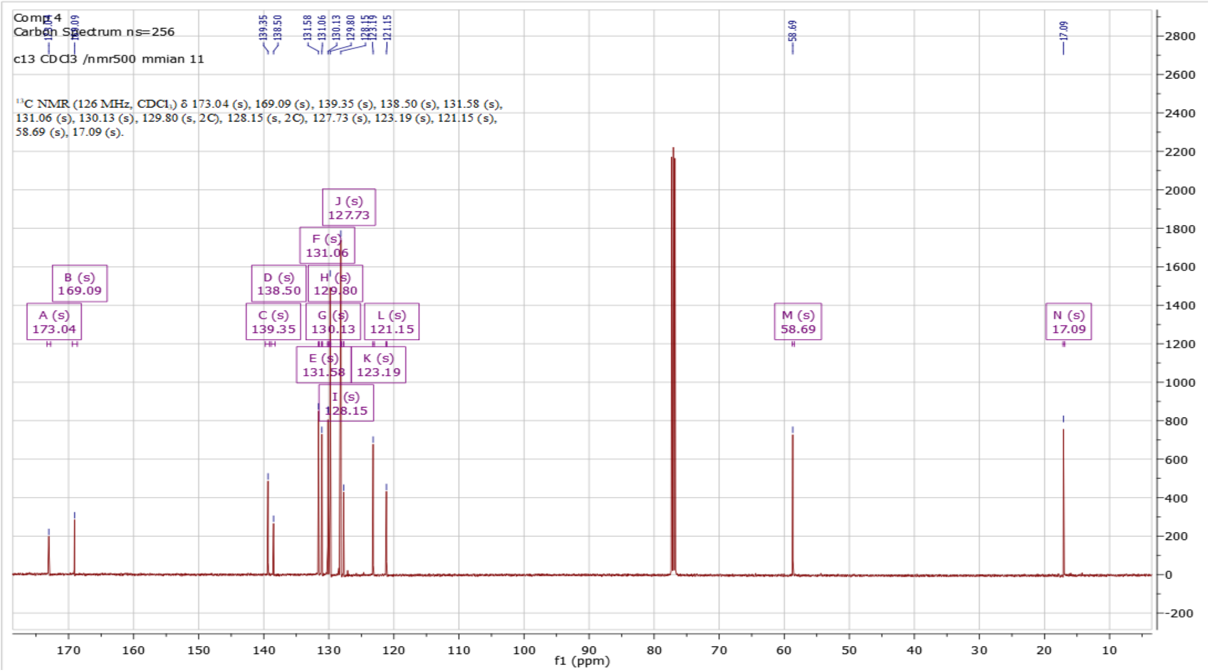

 ©AUTHOR(S)

Formula Predictor Report - COMP 4_12082020 Analysis_45.lcd

Page 1 of 1

Data File: C:\LabSolutions\Data\Vilashini Rajaratnam\12082020 Analysis\COMP 4_12082020 Analysis_45.lcd

Elmt	Val.	Min	Max	Elmt	Val.	Min	Max	Elmt	Val.	Min	Max	Elmt	Val.	Min	Max	Use Adduct
H	1	10	30	N	3	1	3	Si	4	0	0	Br	1	0	0	H
2H	1	0	0	O	2	1	3	S	2	0	0	I	3	0	0	K
C	4	10	25	F	1	0	0	Cl	1	0	0					

Error Margin (ppm): 300
HC Ratio: unlimited
Max Isotopes: all
MSn Iso RI (%): 75.00

DBE Range: -100.0 - 2000.0
Apply N Rule: no
Isotope RI (%): 1.00
MSn Logic Mode: AND

Electron Ions: both
Use MSn Info: yes
Isotope Res: 10000
Max Results: 10

Event#: 1 MS(E+) Ret. Time : 1.480 -> 1.493 - 0.693 -> 0.760 Scan# : 223 -> 225 - 105 -> 115

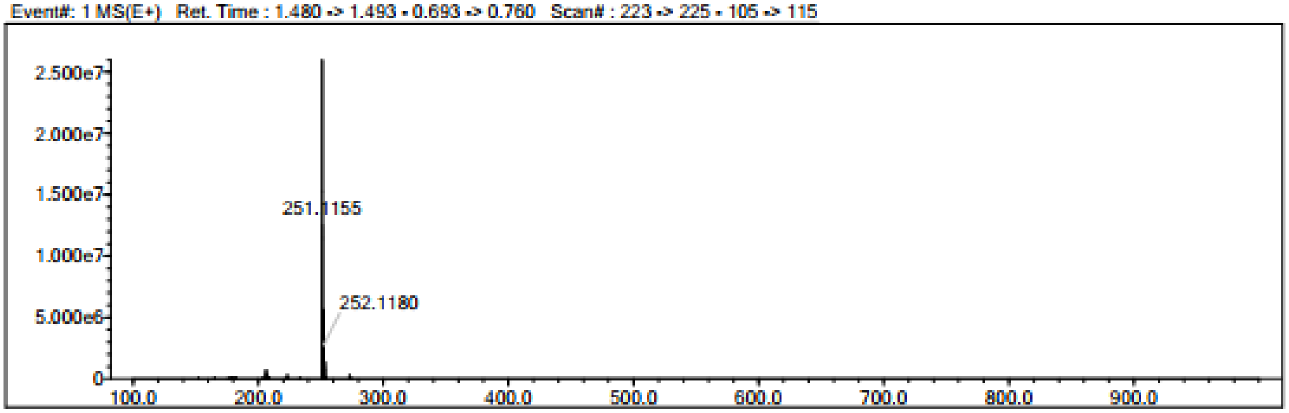

Measured region for 251.1155 m/z

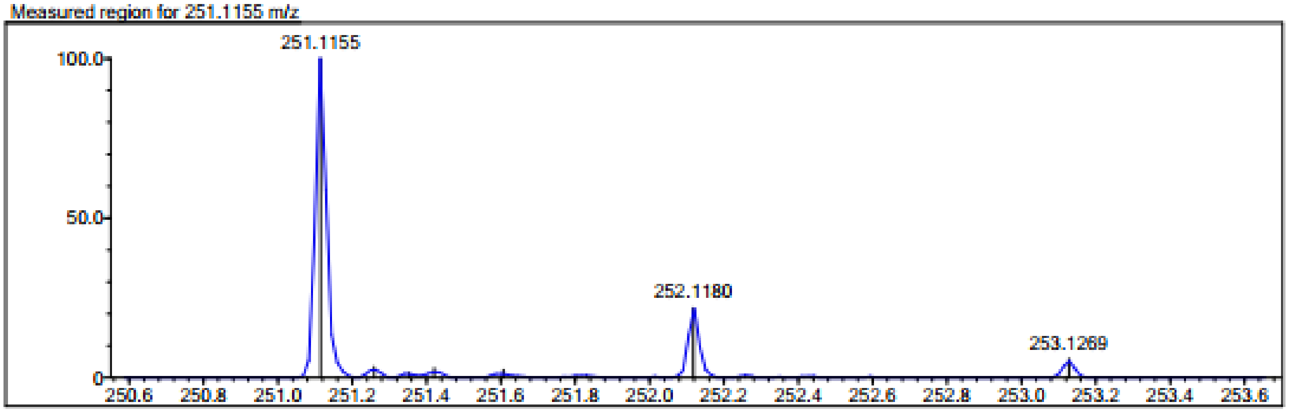

C16 H14 N2 O [M+H]+ : Predicted region for 251.1179 m/z

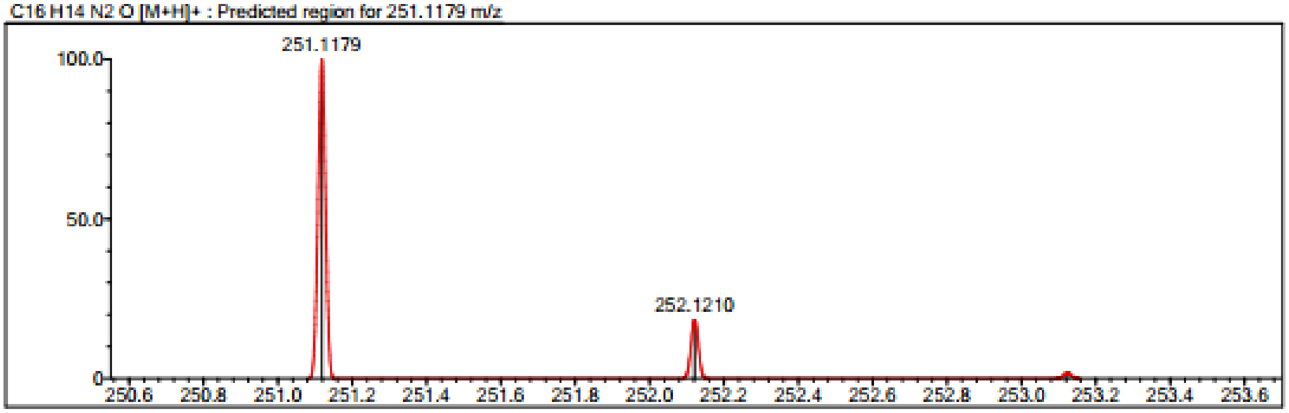

Rank	Score	Formula (M)	Ion	Meas. m/z	Pred. m/z	Df. (mDa)	Df. (ppm)	Iso	DBE
1	25.99	C16 H14 N2 O	[M+H]+	251.1155	251.1179	-2.4	-9.56	58.54	11.0

©AUTHOR(S)

(S)-7-bromo-3-methyl-5-phenyl-1,3-dihydro-2H-benzo[e][1,4]diazepin-2-one(5c)

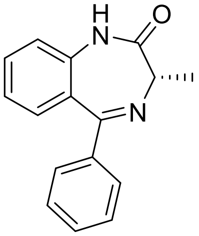

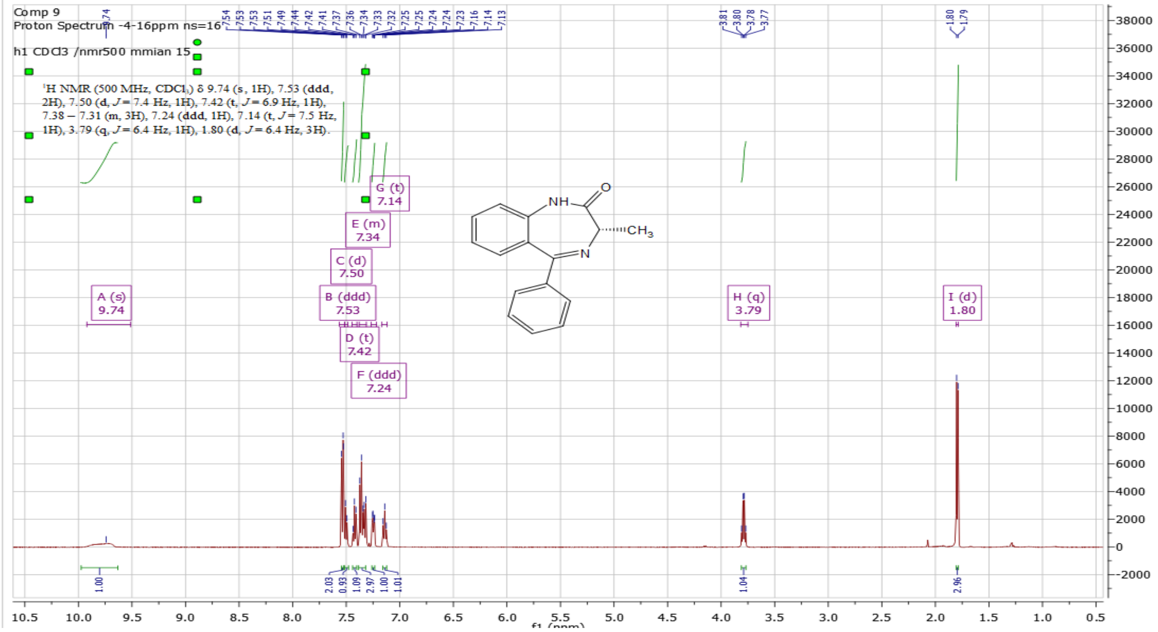

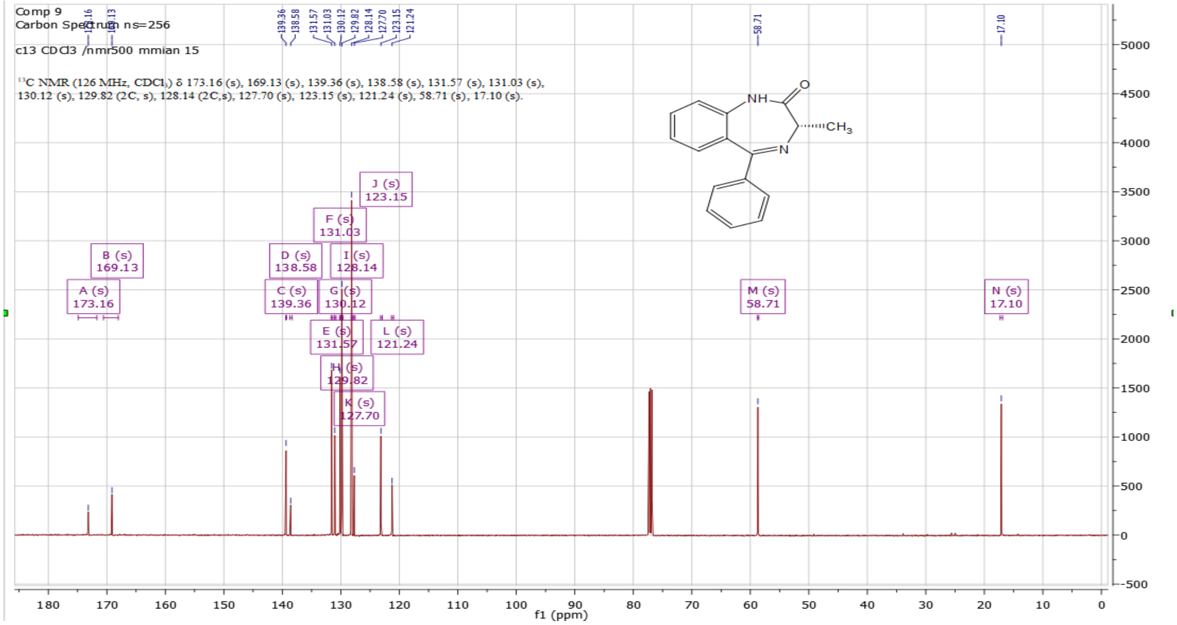

©AUTHOR(S)

Formula Predictor Report - COMP 9_12082020 Analysis_35.lcd Page 1 of 1

Data File: C:\LabSolutions\Data\Vilashini Rajaratnam\12082020 Analysis\COMP 9_12082020 Analysis_35.lcd

Elmt	Val.	Min	Max	Elmt	Val.	Min	Max	Elmt	Val.	Min	Max	Elmt	Val.	Min	Max	Use Adduct
H	1	10	30	N	3	1	3	Si	4	0	0	Br	1	0	0	H
2H	1	0	0	O	2	1	3	S	2	0	0	I	3	0	0	K
C	4	10	25	F	1	0	1	Cl	1	0	1					

Error Margin (ppm): 300
HC Ratio: unlimited
Max Isotopes: all
MSn Iso RI (%): 75.00

DBE Range: -100.0 - 2000.0
Apply N Rule: no
Isotope RI (%): 1.00
MSn Logic Mode: AND

Electron Ions: both
Use MSn Info: yes
Isotope Res: 10000
Max Results: 10

Event#: 1 MS(E+) Ret. Time : 0.920 -> 1.107 - 0.227 -> 0.464 Scan# : 139 -> 167 - 35 -> 71

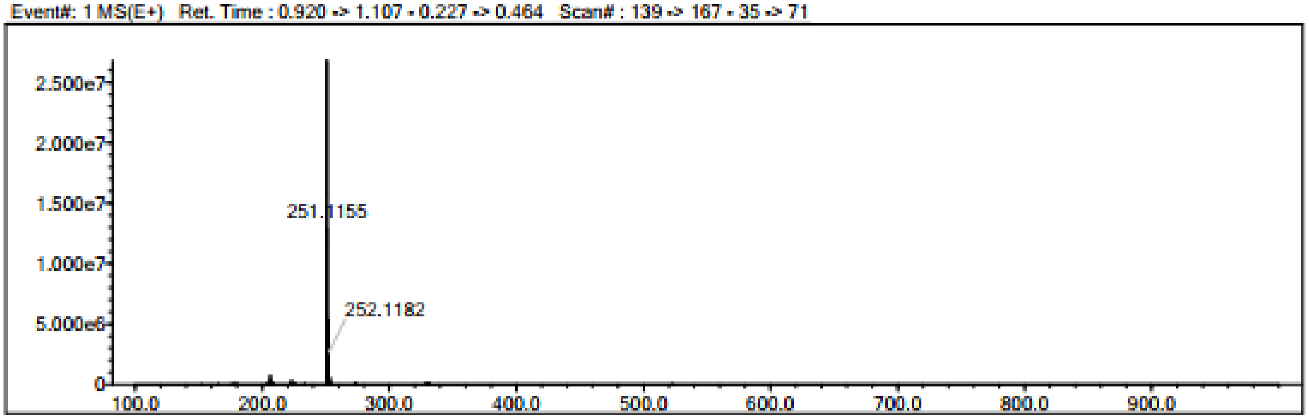

Measured region for 251.1155 m/z

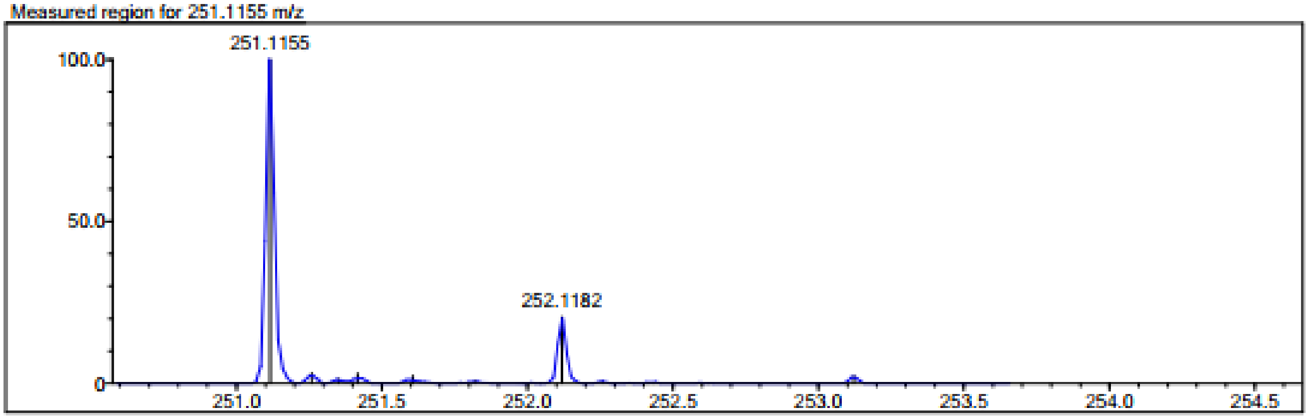

C16 H14 N2 O [M+H]+ : Predicted region for 251.1179 m/z

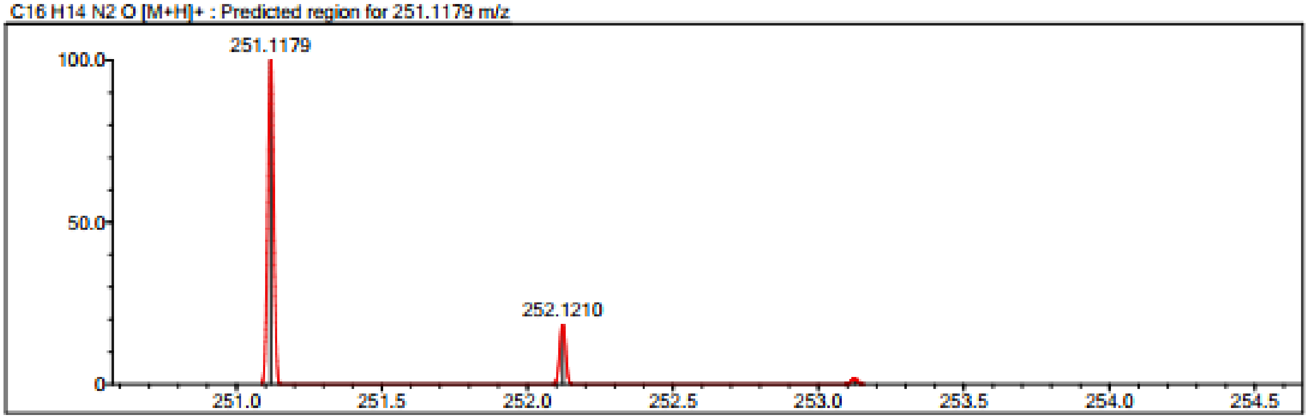

Rank	Score	Formula (M)	Ion	Meas. m/z	Pred. m/z	Df. (mDa)	Df. (ppm)	Iso	DBE
2	25.80	C16 H14 N2 O	[M+H]+	251.1155	251.1179	-2.4	-9.56	58.11	11.0

 ©AUTHOR(S)

(R)-5-(2-fluorophenyl)-3-methyl-1,3-dihydro-2H-benzo[e][1,4]diazepin-2-one(5d)

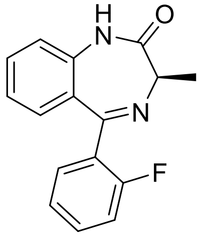

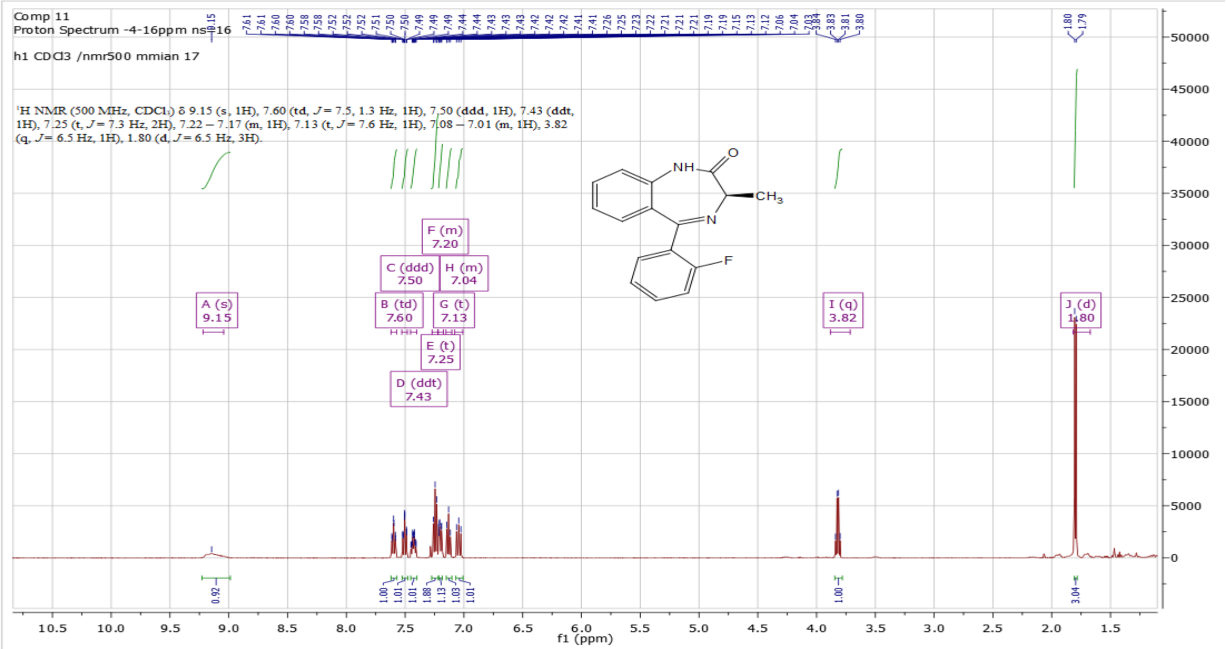

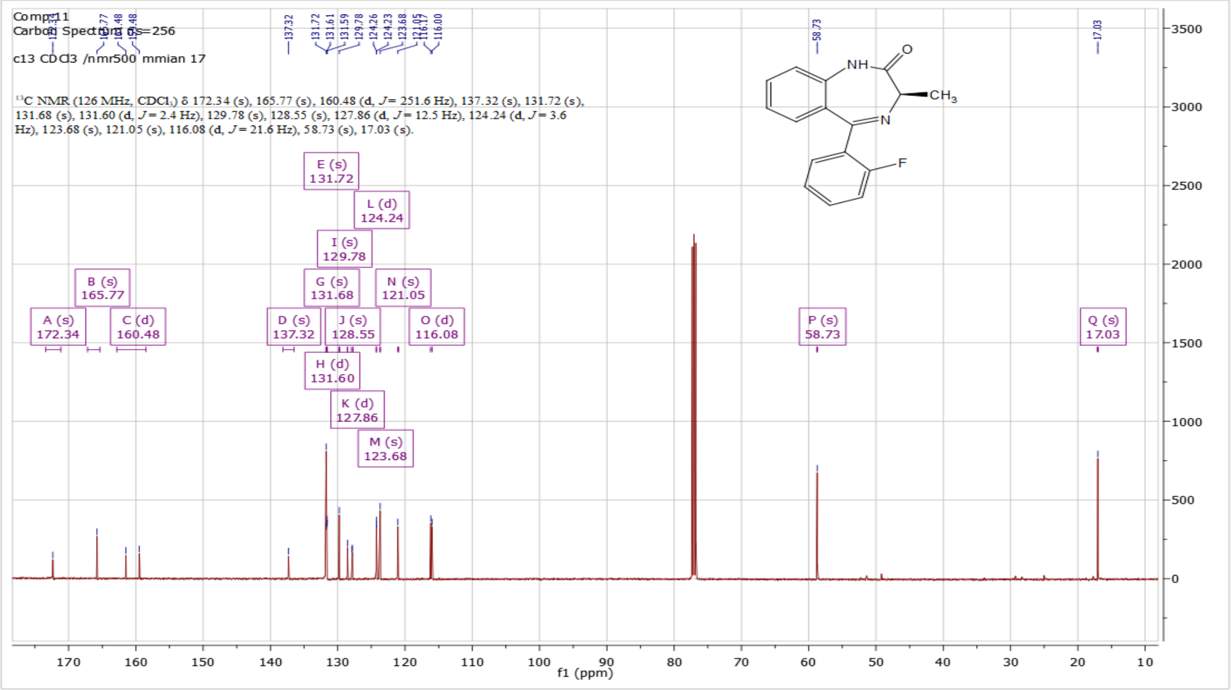

©AUTHOR(S)

Formula Predictor Report - COMP 11_12082020 Analysis_31.lcd Page 1 of 1

Data File: C:\LabSolutions\Data\Vilashini Rajaratnam\12082020 Analysis\COMP 11_12082020 Analysis_31.lcd

Elmt	Val.	Min	Max	Elmt	Val.	Min	Max	Elmt	Val.	Min	Max	Elmt	Val.	Min	Max	Use Adduct
H	1	10	30	N	3	1	3	Si	4	0	0	Br	1	0	0	H
2H	1	0	0	O	2	1	3	S	2	0	0	I	3	0	0	K
C	4	10	25	F	1	0	1	Cl	1	0	1					

Error Margin (ppm):	300	DBE Range:	-100.0 - 2000.0	Electron Ions:	both
HC Ratio:	unlimited	Apply N Rule:	no	Use MSn Info:	yes
Max Isotopes:	all	Isotope RI (%):	1.00	Isotope Res:	10000
MSn Iso RI (%):	75.00	MSn Logic Mode:	AND	Max Results:	10

Event#: 1 MS(E+) Ret. Time : 0.987 -> 1.507 - 0.240 -> 0.420 Scan# : 149 -> 227 - 37 -> 63

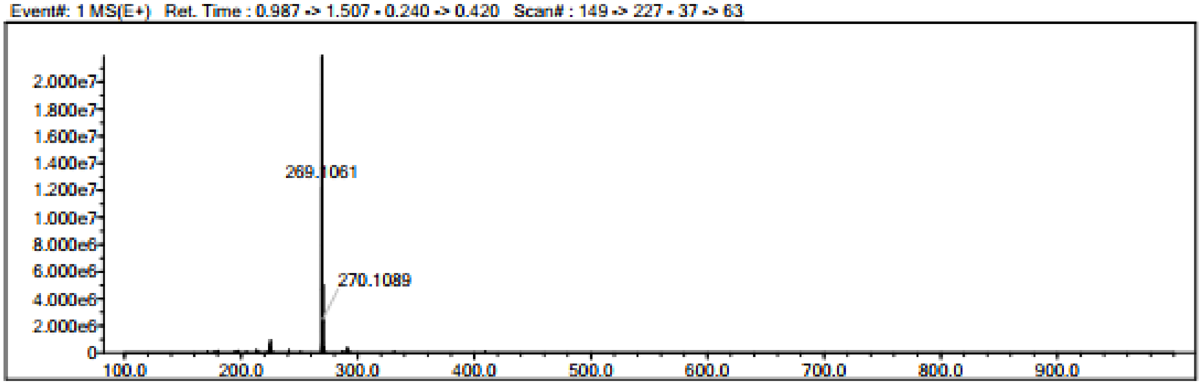

Measured region for 269.1061 m/z

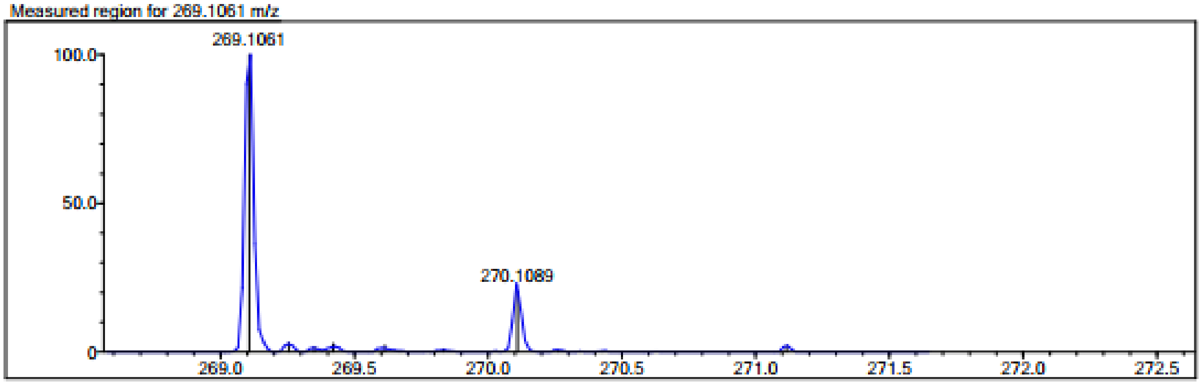

C16 H13 N2 O F [M+H]+ : Predicted region for 269.1085 m/z

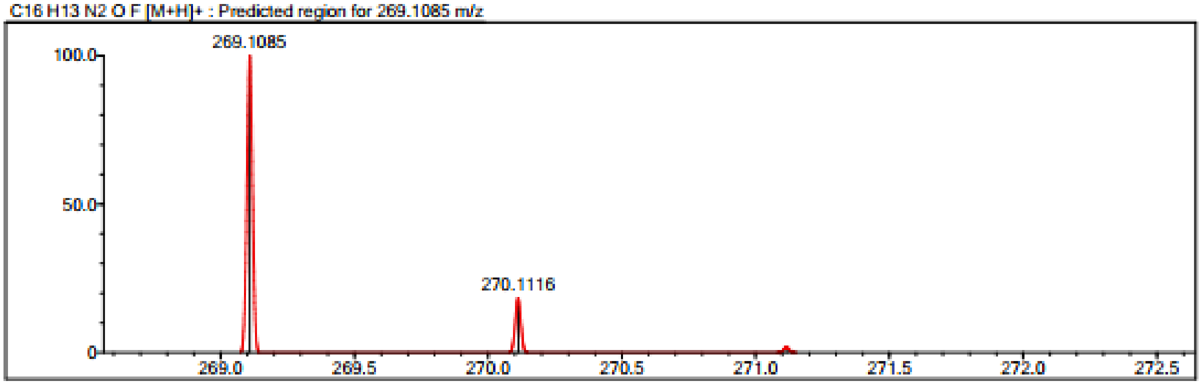

Rank	Score	Formula (M)	Ion	Meas. m/z	Pred. m/z	Df. (mDa)	Df. (ppm)	Iso	DBE
3	47.48	C16 H13 N2 O F	[M+H]+	269.1061	269.1085	-2.4	-8.92	93.47	11.0

 ©AUTHOR(S)

(S)-5-(2-fluorophenyl)-3-methyl-1,3-dihydro-2H-benzo[e][1,4]diazepin-2-one (5e)

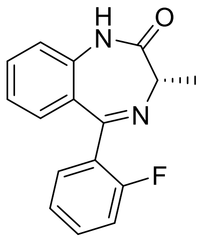

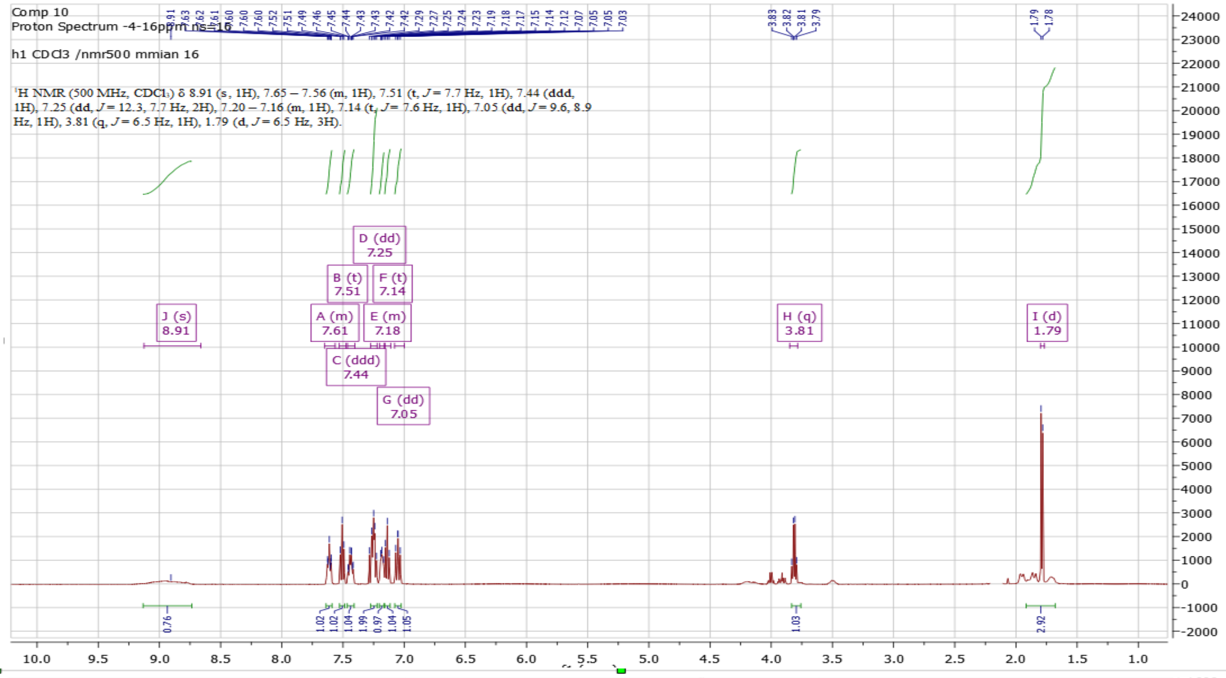

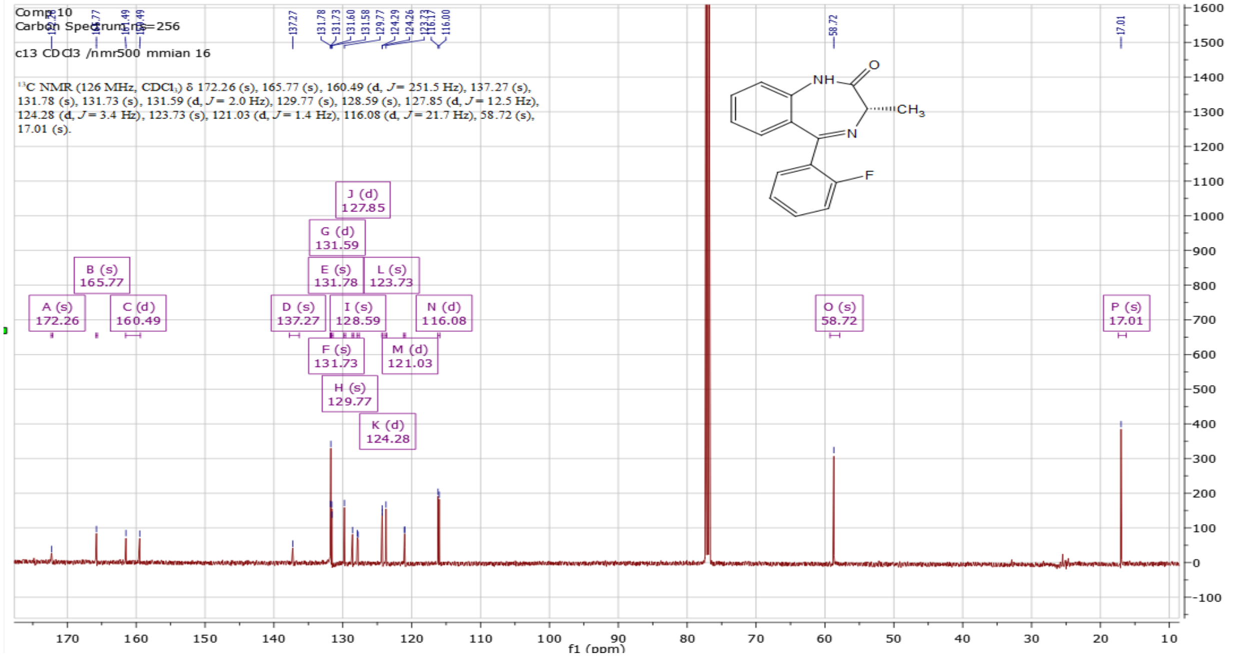

©AUTHOR(S)

Formula Predictor Report - COMP 10_12082020 Analysis_33.lcd Page 1 of 1

Data File: C:\LabSolutions\Data\Vilashini Rajaratnam\12082020 Analysis\COMP 10_12082020 Analysis_33.lcd

Elmt	Val.	Min	Max	Elmt	Val.	Min	Max	Elmt	Val.	Min	Max	Elmt	Val.	Min	Max	Use Adduct
H	1	10	30	N	3	1	3	Si	4	0	0	Br	1	0	0	H
2H	1	0	0	O	2	1	3	S	2	0	0	I	3	0	0	K
C	4	10	25	F	1	0	1	Cl	1	0	1					

Error Margin (ppm):	300	DBE Range:	-100.0 - 2000.0	Electron Ions:	both
HC Ratio:	unlimited	Apply N Rule:	no	Use MSn Info:	yes
Max Isotopes:	all	Isotope RI (%):	1.00	Isotope Res:	10000
MSn Iso RI (%):	75.00	MSn Logic Mode:	AND	Max Results:	10

Event#: 1 MS(E+) Ret. Time : 1.533 -> 1.680 - 0.067 -> 1.089 Scan# : 231 -> 253 - 11 -> 165

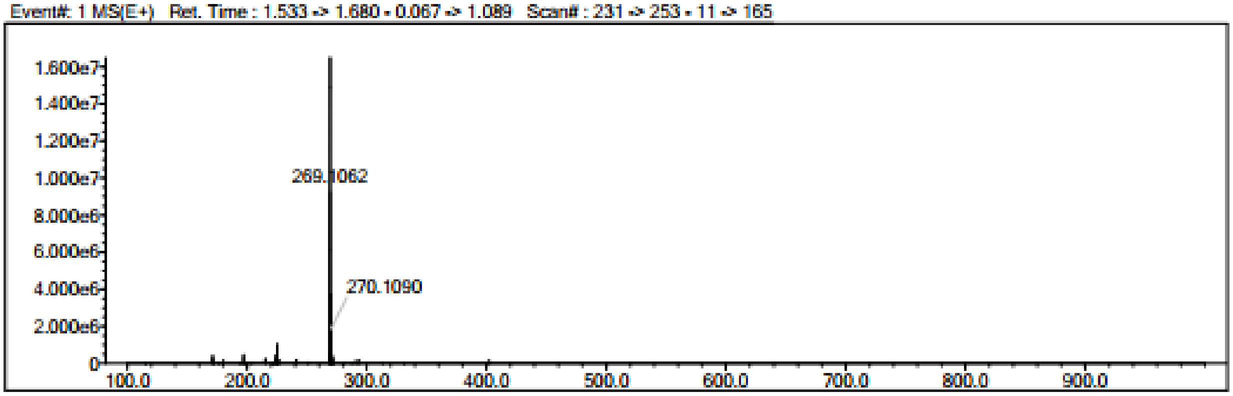

Measured region for 269.1062 m/z

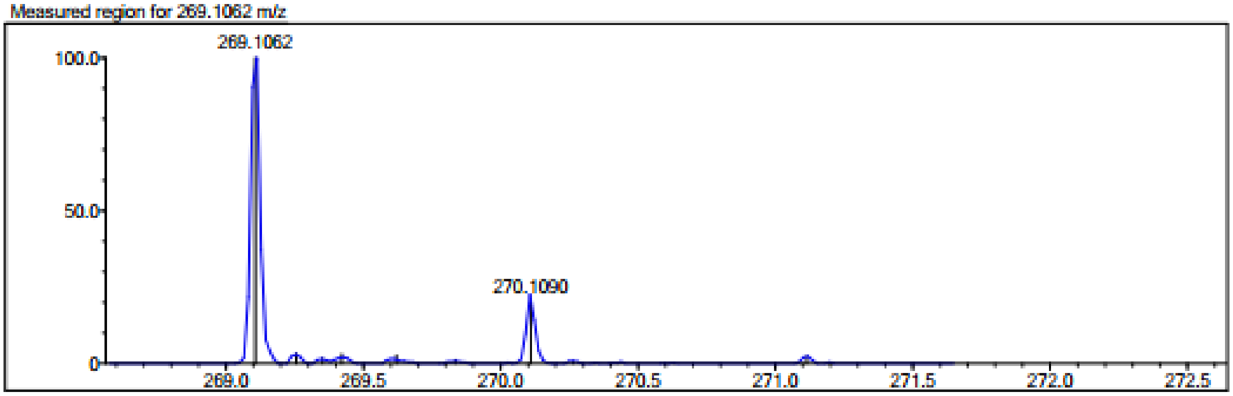

C16 H13 N2 O F [M+H]+ : Predicted region for 269.1085 m/z

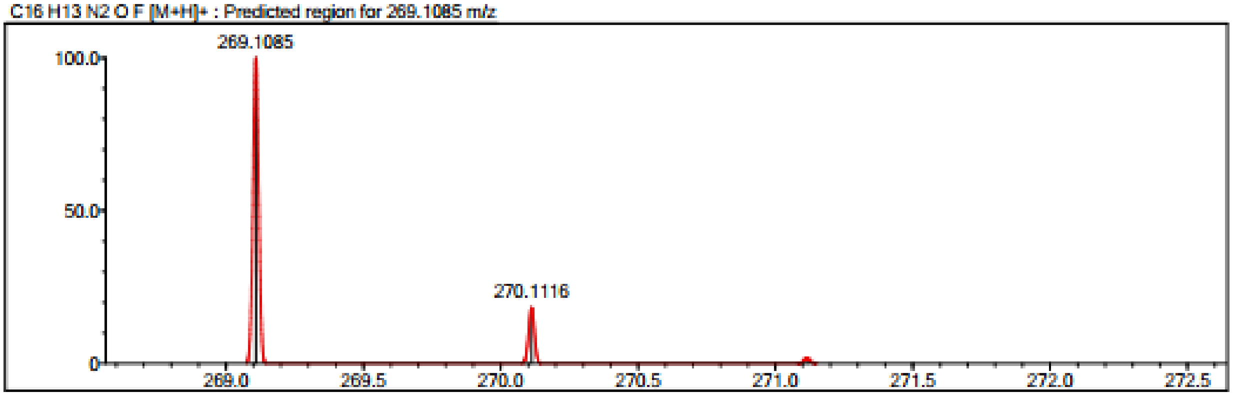

Rank	Score	Formula (M)	Ion	Meas. m/z	Pred. m/z	Df. (mDa)	Df. (ppm)	Iso	DBE
4	46.02	C16 H13 N2 O F	[M+H]+	269.1062	269.1085	-2.3	-8.55	84.45	11.0

©AUTHOR(S)

(S)-5-(2-chlorophenyl)-3-methyl-1,3-dihydro-2H-benzo[e][1,4]diazepin-2-one (5f)

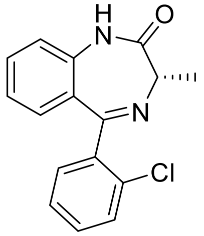

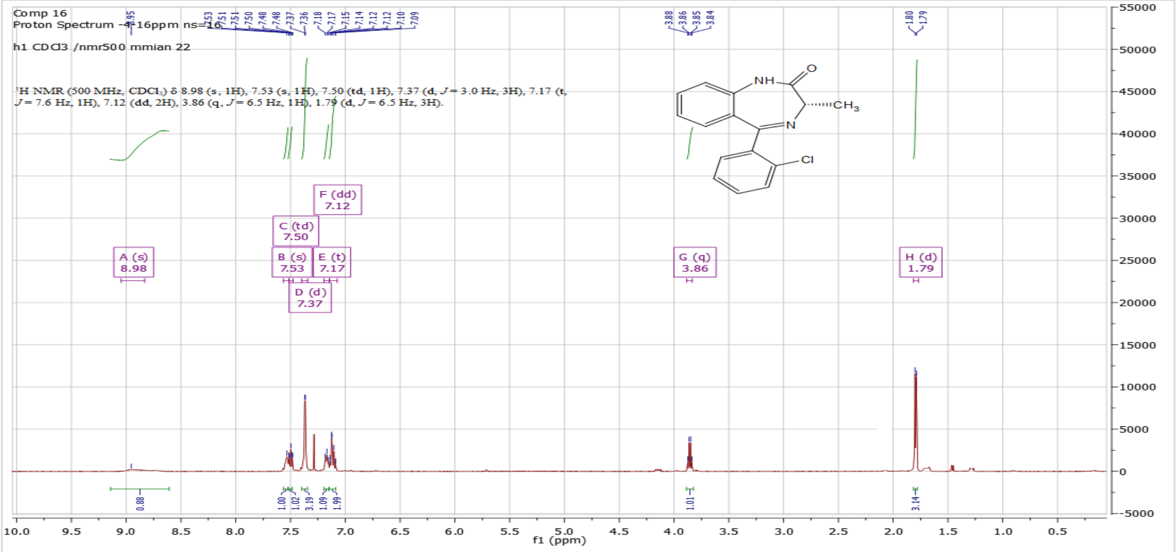

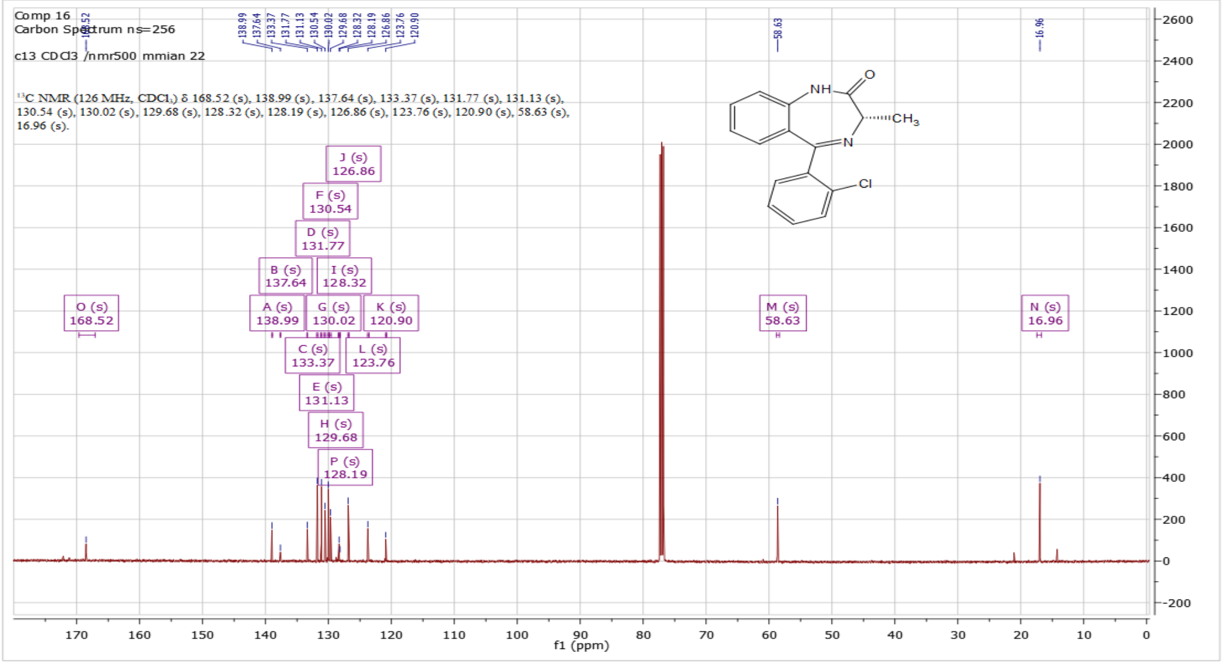

 ©AUTHOR(S)

Formula Predictor Report - MYMV6_12082020 Analysis_19.lcd Page 1 of 1

Data File: C:\LabSolutions\Data\Vilashini Rajaratnam\12082020 Analysis\MYMV6_12082020 Analysis_19.lcd

Elmt	Val.	Min	Max	Elmt	Val.	Min	Max	Elmt	Val.	Min	Max	Elmt	Val.	Min	Max	Use Adduct
H	1	10	30	N	3	1	3	Si	4	0	0	Br	1	0	0	H
2H	1	0	0	O	2	1	3	S	2	0	0	I	3	0	0	K
C	4	10	25	F	1	0	0	Cl	1	0	1					

Error Margin (ppm): 300
HC Ratio: unlimited
Max Isotopes: all
MSn Iso RI (%): 75.00
DBE Range: -100.0 - 2000.0
Apply N Rule: no
Isotope RI (%): 1.00
MSn Logic Mode: AND
Electron Ions: both
Use MSn Info: yes
Isotope Res: 10000
Max Results: 10

Event#: 1 MS(E+) Ret. Time : 1.253 -> 1.427 - 0.120 -> 0.471 Scan# : 189 -> 215 - 19 -> 71

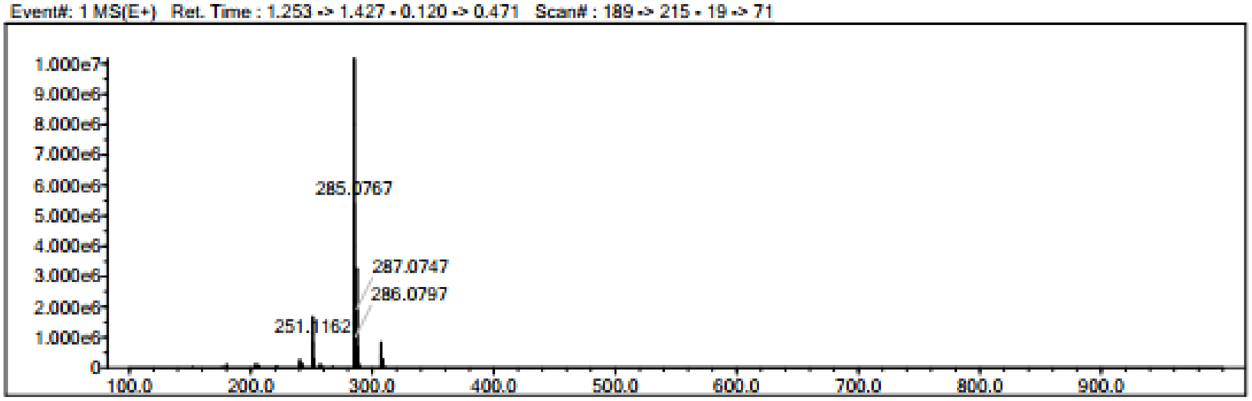

Measured region for 285.0767 m/z

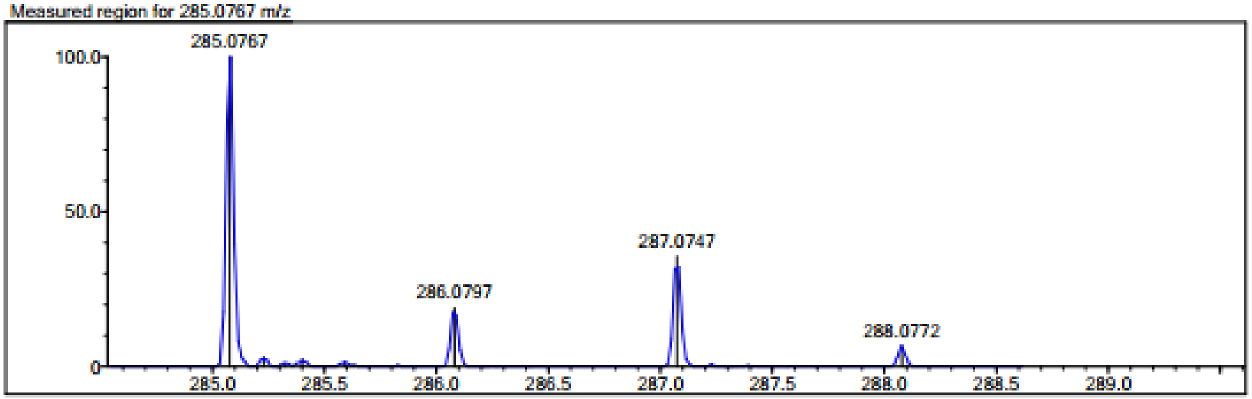

C16 H13 N2 O Cl [M+H]+ : Predicted region for 285.0789 m/z

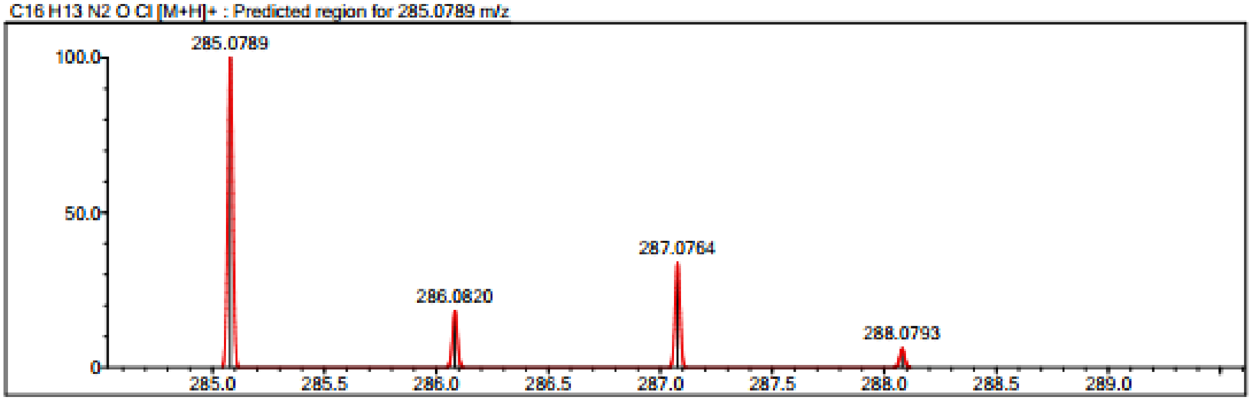

Rank	Score	Formula (M)	Ion	Meas. m/z	Pred. m/z	Df. (mDa)	Df. (ppm)	Iso	DBE
1	62.41	C16 H13 N2 O Cl	[M+H]+	285.0767	285.0789	-2.2	-7.72	99.38	11.0

 ©AUTHOR(S)

(S)-3-methyl-5-(pyridin-2-yl)-1,3-dihydro-2H-benzo[e][1,4]diazepin-2-one(5g)

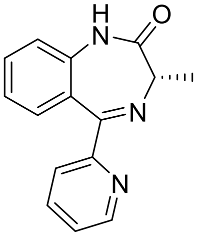

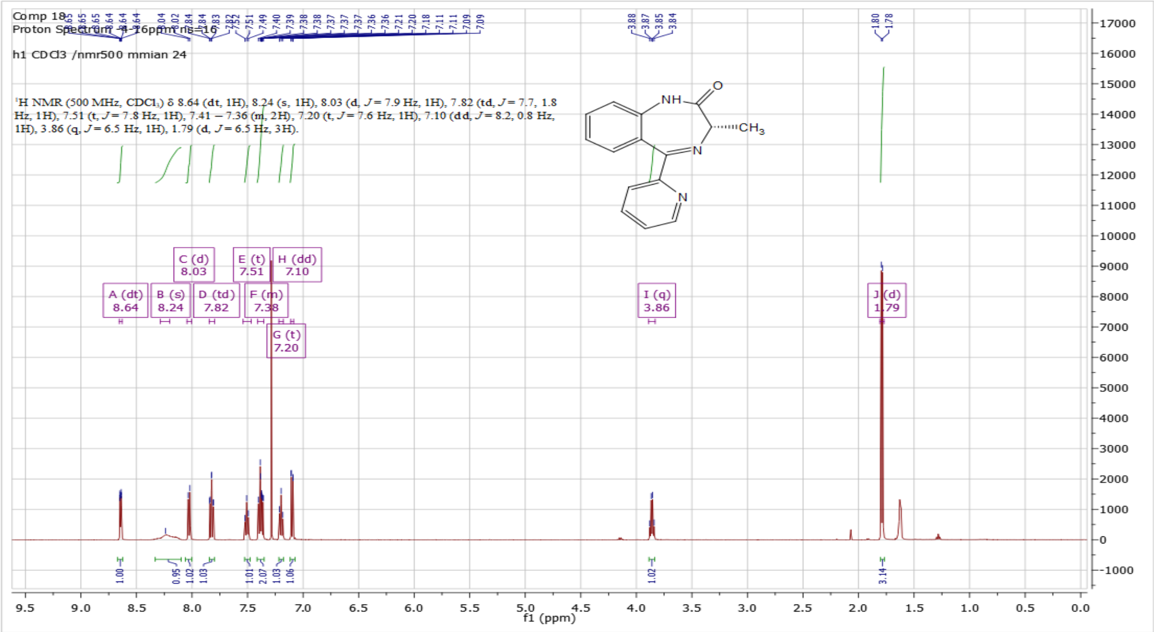

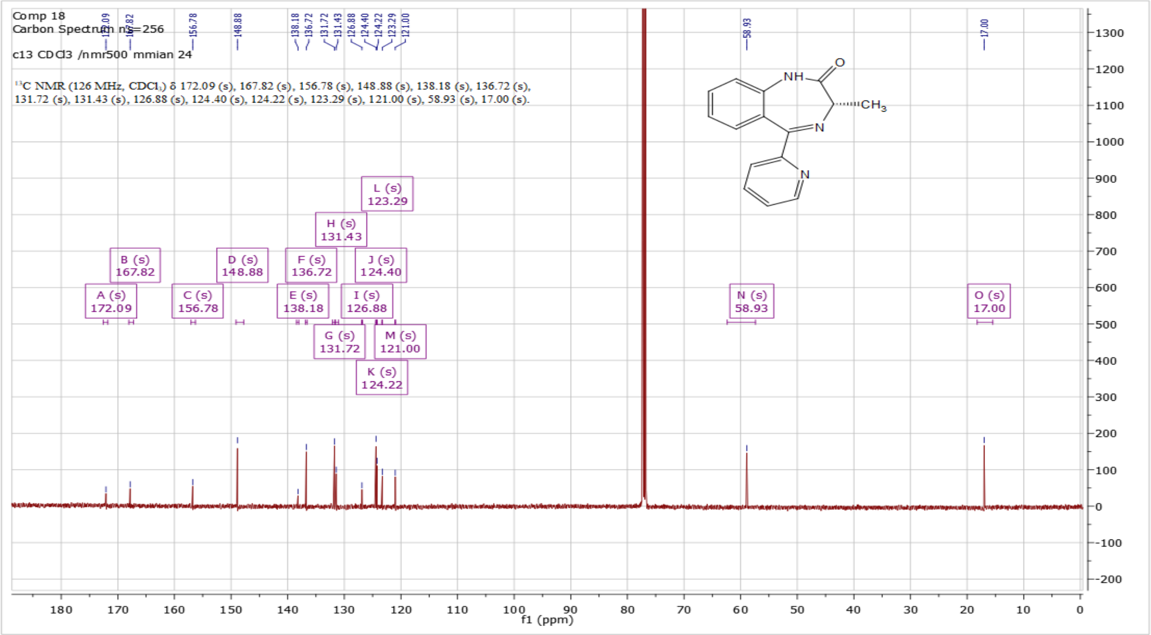

 ©AUTHOR(S)

Formula Predictor Report - COMP 18_12082020 Analysis_21.lcd

Page 1 of 1

Data File: C:\LabSolutions\Data\Vilashini Rajaratnam\12082020 Analysis\COMP 18_12082020 Analysis_21.lcd

Elmt	Val.	Min	Max	Elmt	Val.	Min	Max	Elmt	Val.	Min	Max	Elmt	Val.	Min	Max	Use Adduct
H	1	10	30	N	3	1	3	Si	4	0	0	Br	1	0	0	H
2H	1	0	0	O	2	1	3	S	2	0	0	I	3	0	0	K
C	4	10	25	F	1	0	0	Cl	1	0	1					

Error Margin (ppm):	300	DBE Range:	-100.0 - 2000.0	Electron Ions:	both
HC Ratio:	unlimited	Apply N Rule:	no	Use MSn Info:	yes
Max Isotopes:	all	Isotope RI (%):	1.00	Isotope Res:	10000
MSn Iso RI (%):	75.00	MSn Logic Mode:	AND	Max Results:	10

Event#: 1 MS(E+) Ret. Time : 1.133 -> 1.507 - 0.253 -> 0.537 Scan# : 171 -> 227 - 39 -> 81

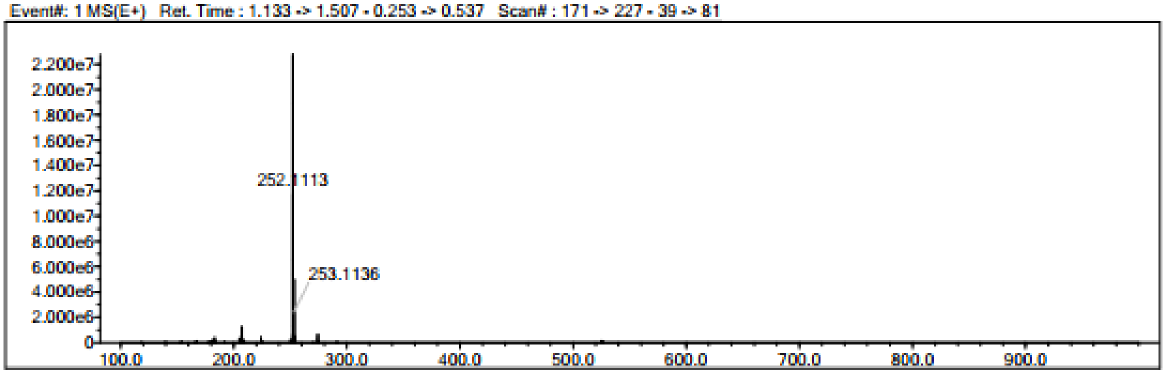

Measured region for 252.1113 m/z

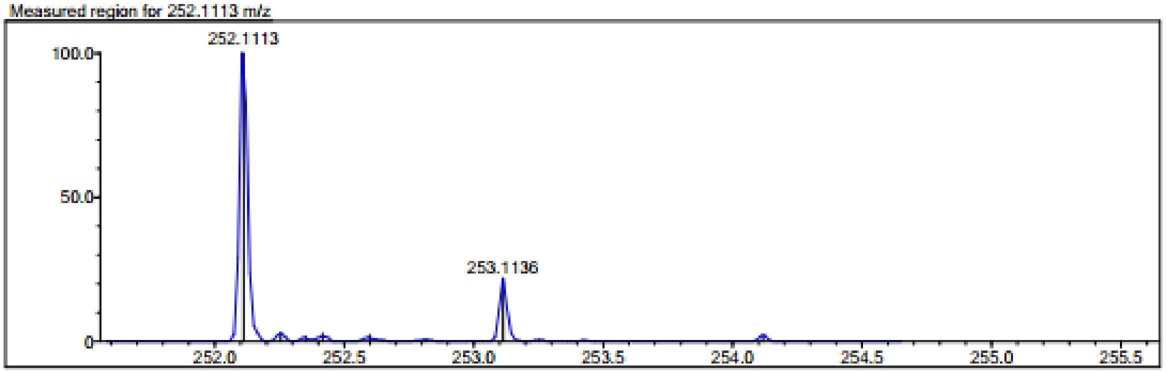

C15 H13 N3 O [M+H]+ : Predicted region for 252.1131 m/z

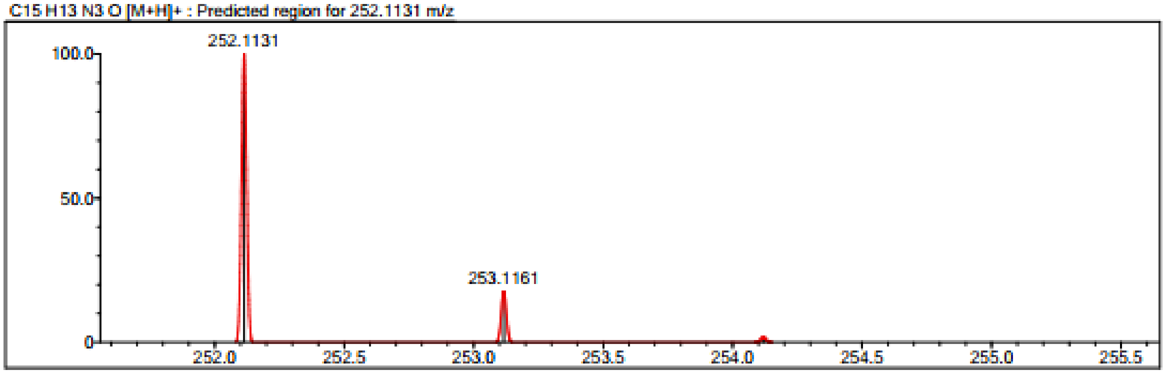

Rank	Score	Formula (M)	Ion	Meas. m/z	Pred. m/z	Df. (mDa)	Df. (ppm)	Iso	DBE
1	43.70	C15 H13 N3 O	[M+H]+	252.1113	252.1131	-1.8	-7.14	63.70	11.0

©AUTHOR(S)

5-(2-fluorophenyl)-2-oxo-2,3-dihydro-1H-benzo[e][1,4]diazepin-3-yl acetate(5h)

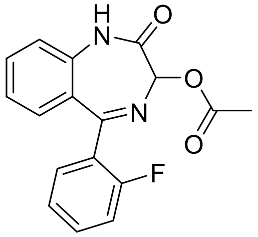

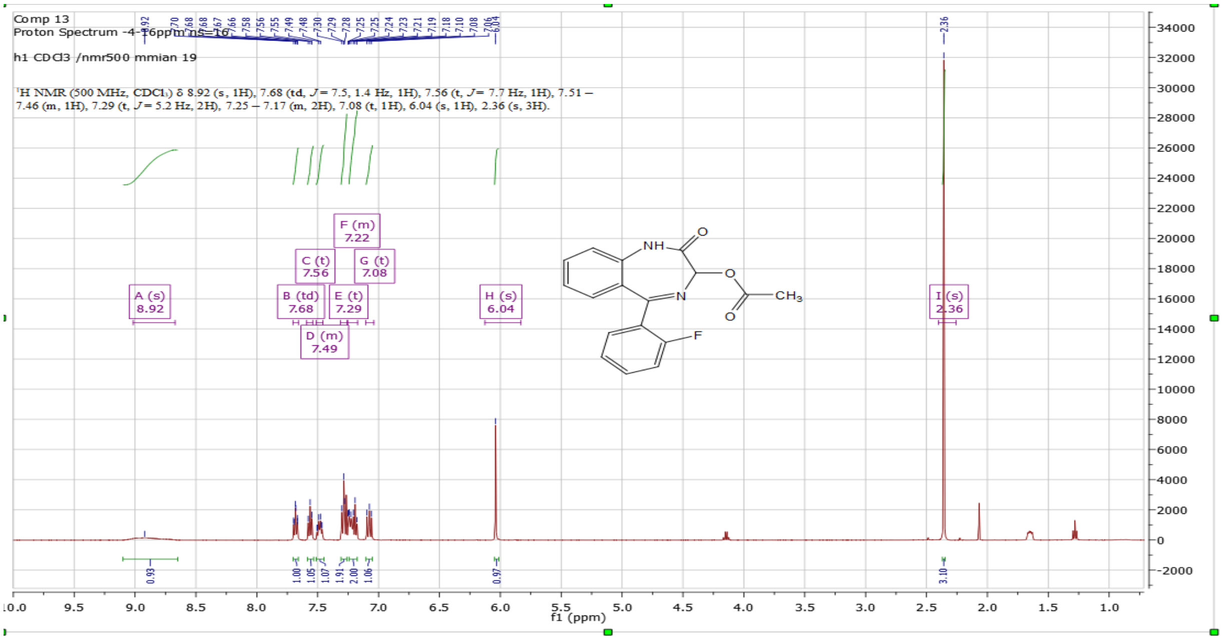

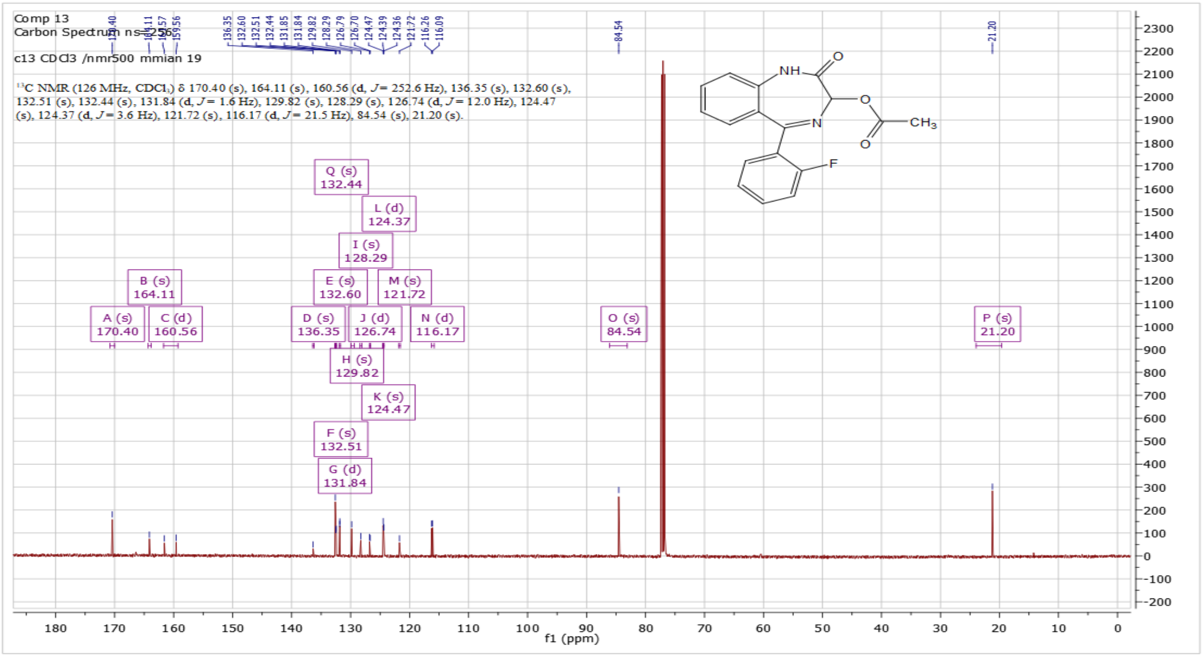

 ©AUTHOR(S)

Formula Predictor Report - COMP 13_12082020 Analysis_27.lcd Page 1 of 1

Data File: C:\LabSolutions\Data\Vilashini Rajaratnam\12082020 Analysis\COMP 13_12082020 Analysis_27.lcd

Elmt	Val.	Min	Max	Elmt	Val.	Min	Max	Elmt	Val.	Min	Max	Elmt	Val.	Min	Max	Use Adduct
H	1	10	30	N	3	1	3	Si	4	0	0	Br	1	0	0	H
2H	1	0	0	O	2	1	3	S	2	0	0	I	3	0	0	K
C	4	10	25	F	1	0	1	Cl	1	0	1					

Error Margin (ppm):	300	DBE Range:	-100.0 - 2000.0	Electron Ions:	both
HC Ratio:	unlimited	Apply N Rule:	no	Use MSn Info:	yes
Max Isotopes:	all	Isotope RI (%):	1.00	Isotope Res:	10000
MSn Iso RI (%):	75.00	MSn Logic Mode:	AND	Max Results:	10

Event#: 1 MS(E+) Ret. Time : 1.147 -> 1.480 - 0.240 -> 0.600 Scan# : 173 -> 223 - 37 -> 91

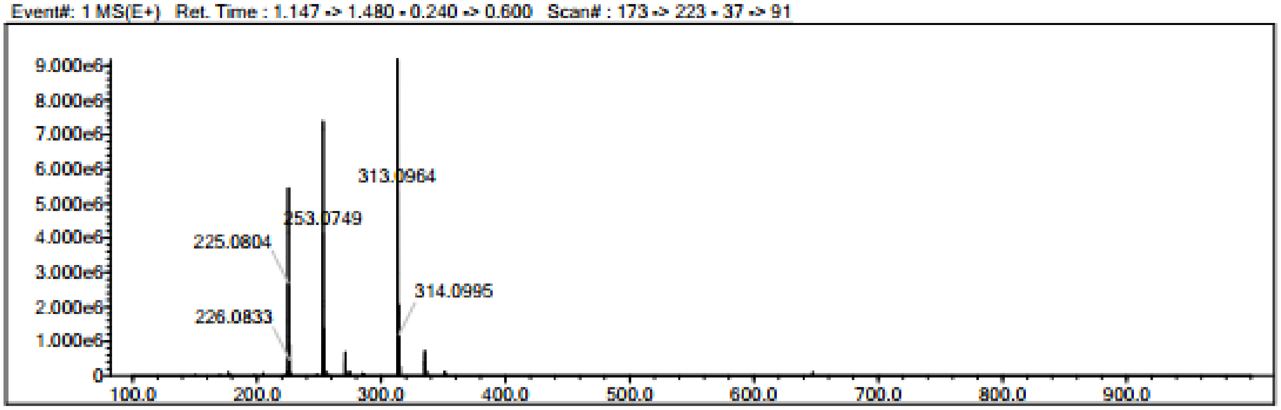

Measured region for 313.0964 m/z

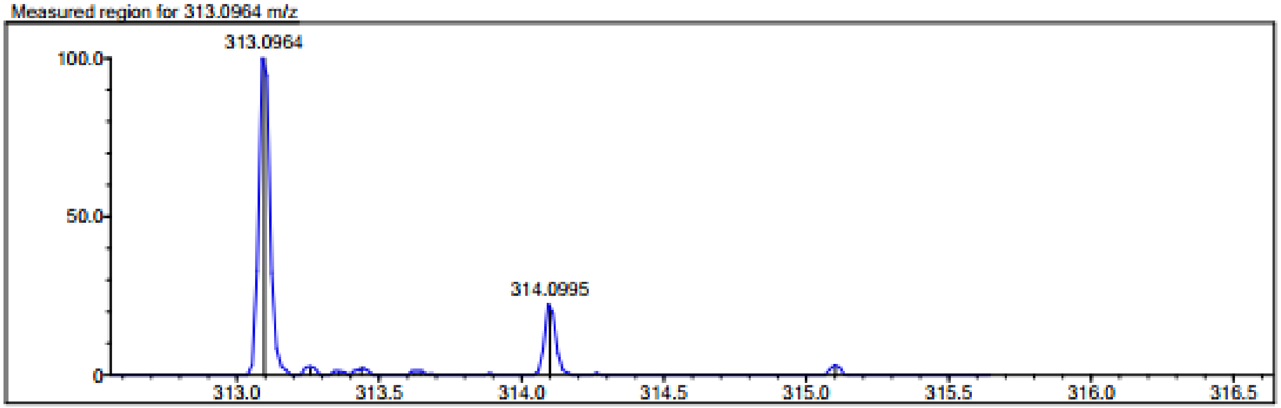

C17 H13 N2 O3 F [M+H]+ : Predicted region for 313.0983 m/z

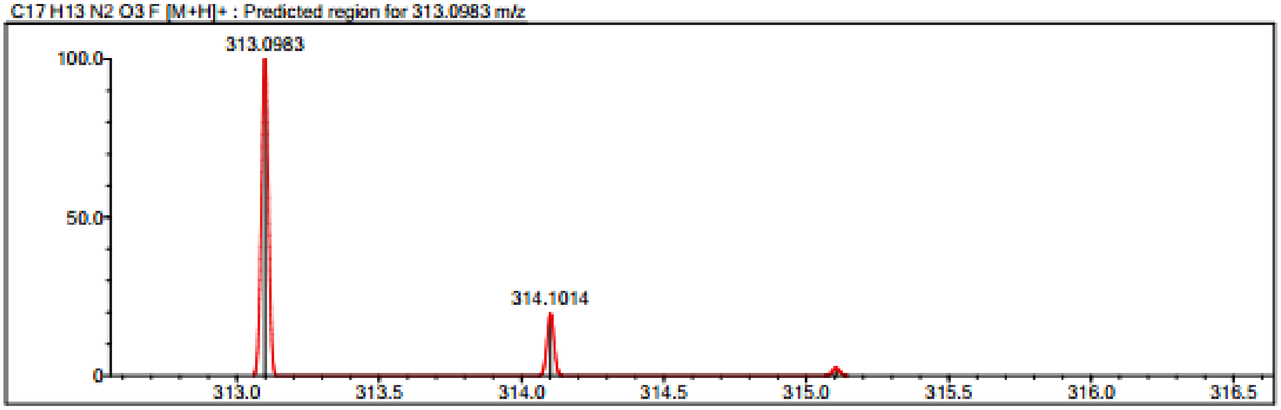

Rank	Score	Formula (M)	Ion	Meas. m/z	Pred. m/z	Df. (mDa)	Df. (ppm)	Iso	DBE
3	74.08	C17 H13 N2 O3 F	[M+H]+	313.0964	313.0983	-1.9	-6.07	93.42	12.0

 ©AUTHOR(S)

(2-aminophenyl)(phenyl)methanone (7a)

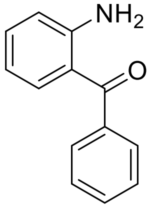

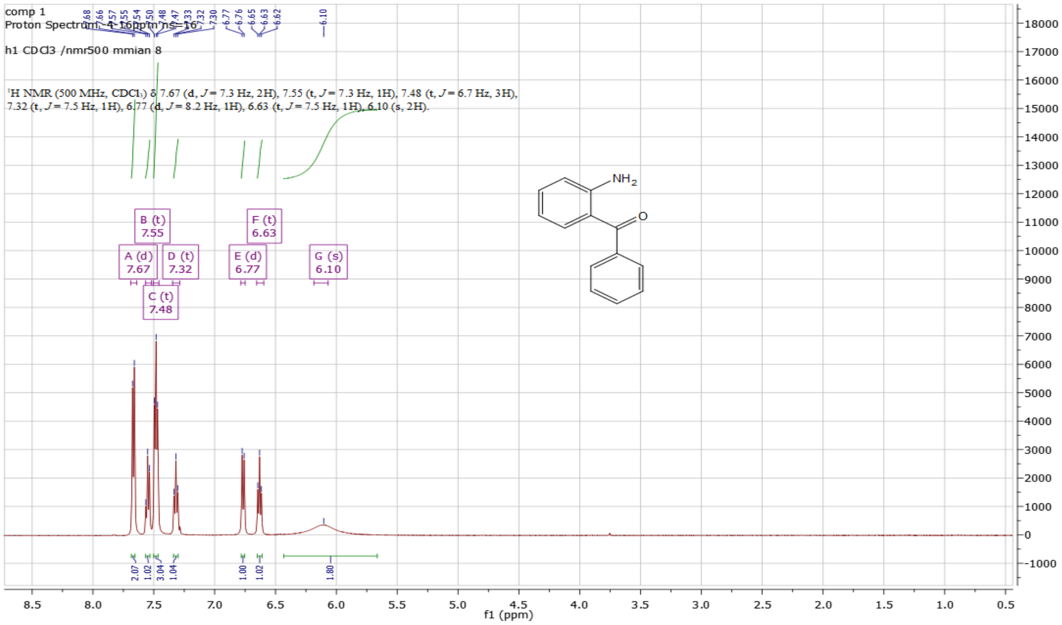

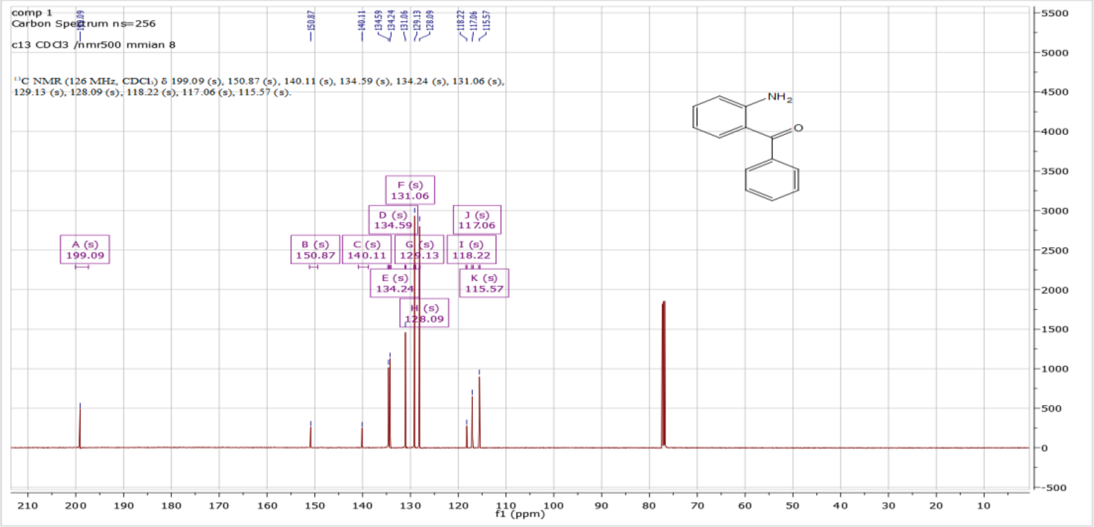

©AUTHOR(S)

Formula Predictor Report - COMP 1_12082020 Analysis_51.lcd Page 1 of 1

Data File: C:\LabSolutions\Data\Vilashini Rajaratnam\12082020 Analysis\COMP 1_12082020 Analysis_51.lcd

Elmt	Val.	Min	Max	Elmt	Val.	Min	Max	Elmt	Val.	Min	Max	Elmt	Val.	Min	Max	Use Adduct
H	1	10	15	N	3	1	3	Si	4	0	0	Br	1	0	0	H
2H	1	0	0	O	2	1	3	S	2	0	0	I	3	0	0	K
C	4	10	25	F	1	0	1	Cl	1	0	0					

Error Margin (ppm):	300	DBE Range:	-100.0 - 2000.0	Electron Ions:	both
HC Ratio:	unlimited	Apply N Rule:	no	Use MSn Info:	yes
Max Isotopes:	all	Isotope RI (%):	1.00	Isotope Res:	10000
MSn Iso RI (%):	75.00	MSn Logic Mode:	AND	Max Results:	10

Event#: 1 MS(E+) Ret. Time : 0.960 -> 1.213 - 0.160 -> 0.193 Scan# : 145 -> 183 - 25 -> 29

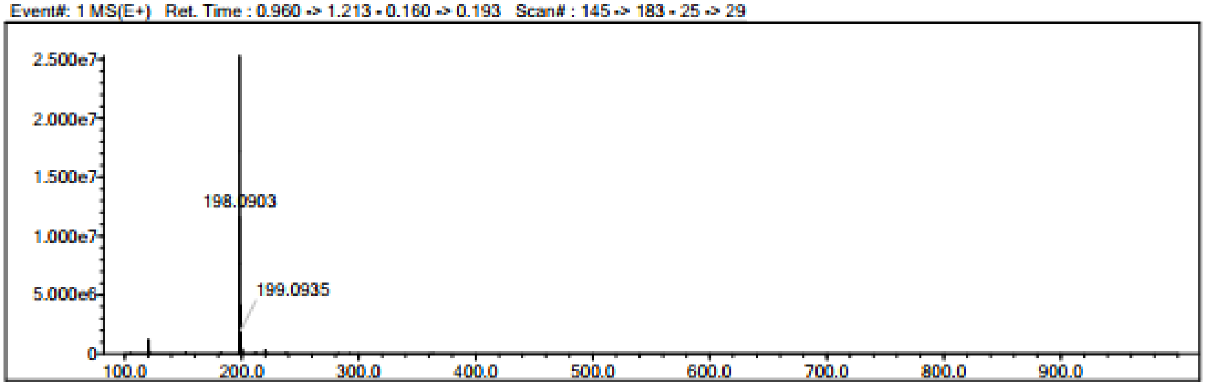

Measured region for 198.0903 m/z

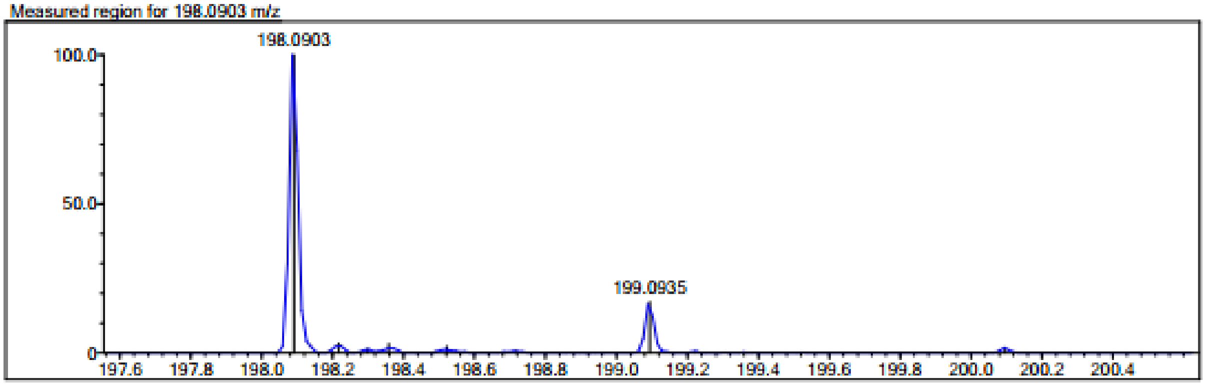

C13 H11 N O [M+H]+ : Predicted region for 198.0913 m/z

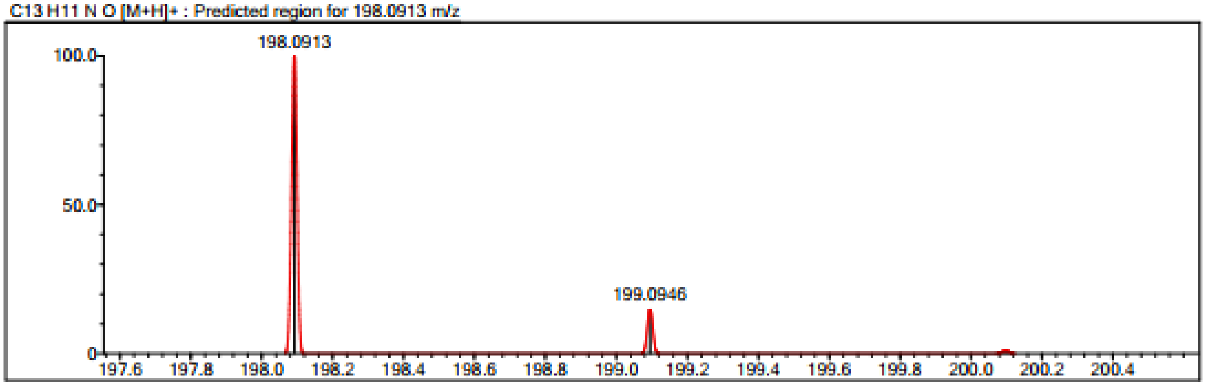

Rank	Score	Formula (M)	Ion	Meas. m/z	Pred. m/z	Df. (mDa)	Df. (ppm)	Iso	DBE
1	53.40	C13 H11 N O	[M+H]+	198.0903	198.0913	-1.0	-5.05	59.66	9.0

 ©AUTHOR(S)

(2-aminophenyl)(2-fluorophenyl)methanone(7b)

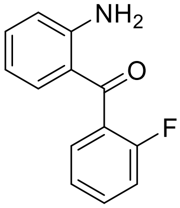

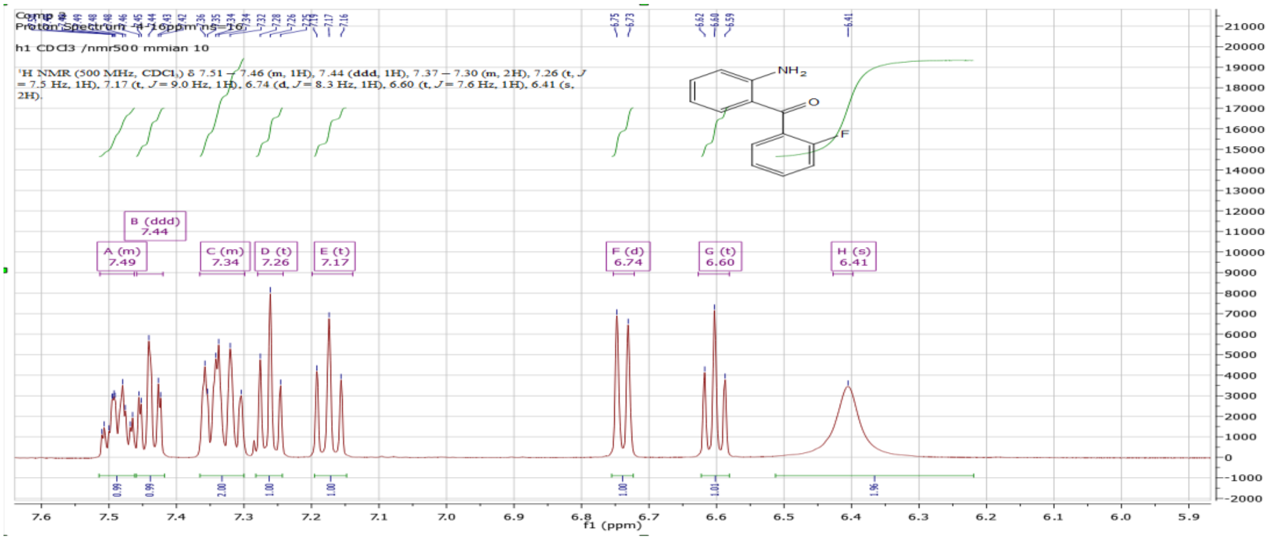

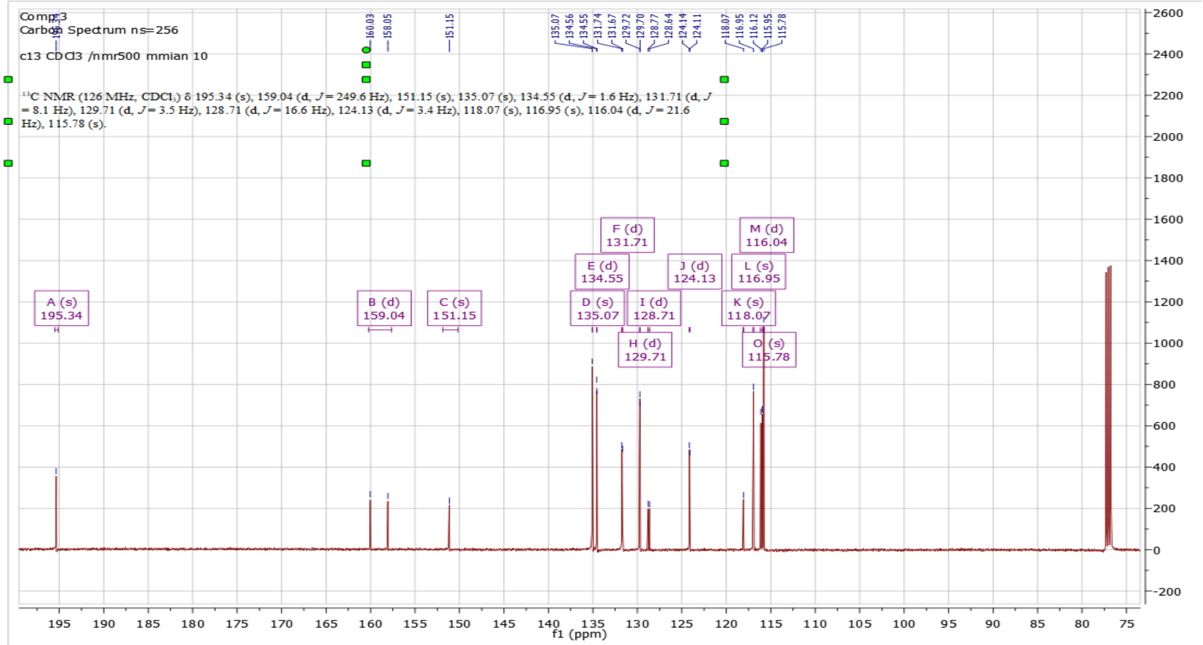

©AUTHOR(S)

Formula Predictor Report - COMP 3_12082020 Analysis_47.lcd Page 1 of 1

Data File: C:\LabSolutions\Data\Vilashini Rajaratnam\12082020 Analysis\COMP 3_12082020 Analysis_47.lcd

Elmt	Val.	Min	Max	Elmt	Val.	Min	Max	Elmt	Val.	Min	Max	Elmt	Val.	Min	Max	Use Adduct
H	1	10	30	N	3	1	3	Si	4	0	0	Br	1	0	0	H
2H	1	0	0	O	2	1	3	S	2	0	0	I	3	0	0	K
C	4	10	25	F	1	0	1	Cl	1	0	0					

Error Margin (ppm): 300
HC Ratio: unlimited
Max Isotopes: all
MSn Iso RI (%): 75.00

DBE Range: -100.0 - 2000.0
Apply N Rule: no
Isotope RI (%): 1.00
MSn Logic Mode: AND

Electron Ions: both
Use MSn Info: yes
Isotope Res: 10000
Max Results: 10

Event#: 1 MS(E+) Ret. Time : 1.480 -> 1.533 - 0.200 -> 0.725 Scan# : 223 -> 231 - 31 -> 109

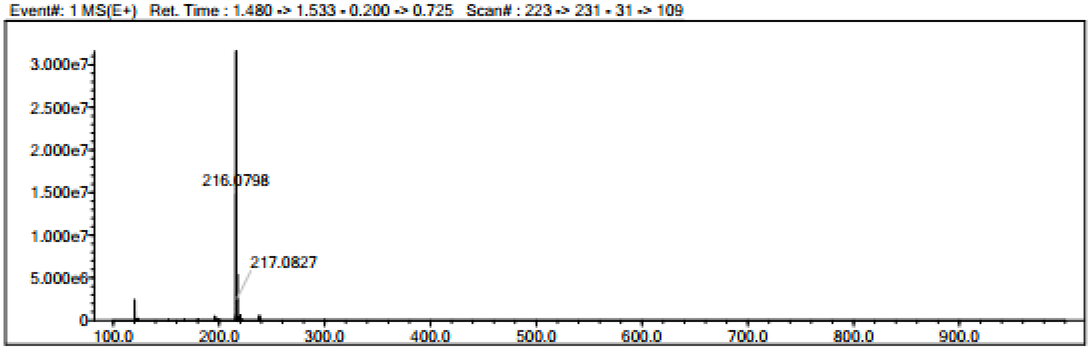

Measured region for 216.0798 m/z

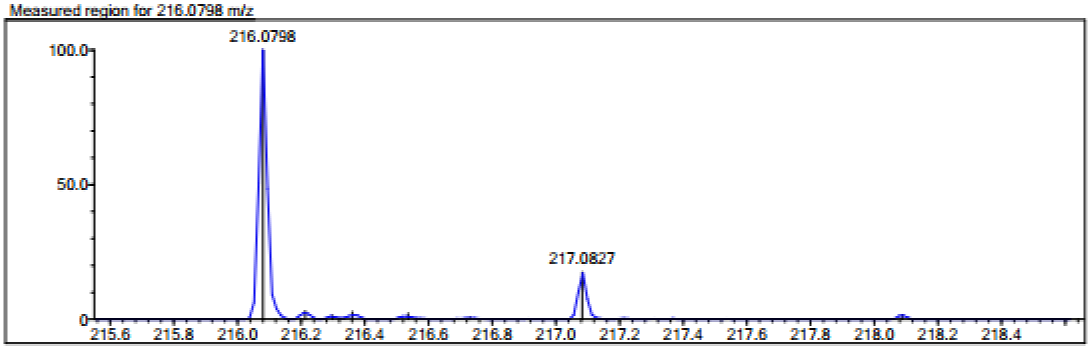

C13 H10 N O F [M+H]+ : Predicted region for 216.0819 m/z

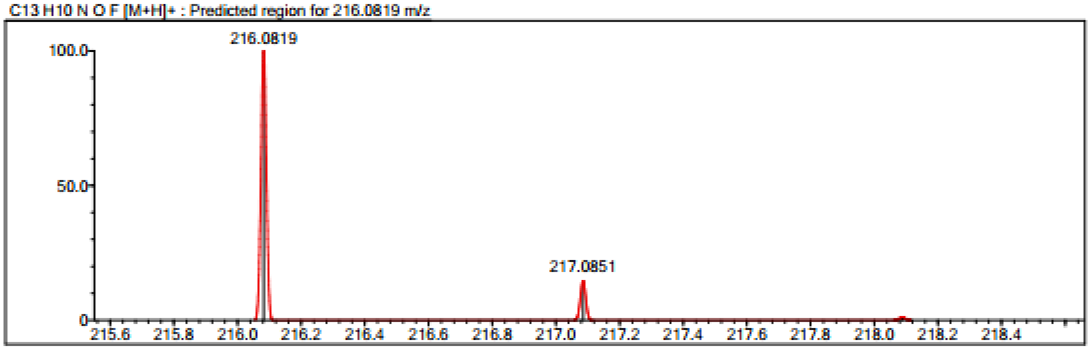

Rank	Score	Formula (M)	Ion	Meas. m/z	Pred. m/z	Df. (mDa)	Df. (ppm)	Iso	DBE
2	33.60	C13 H10 N O F	[M+H]+	216.0798	216.0819	-2.1	-9.72	78.51	9.0

 ©AUTHOR(S)

(2-aminophenyl)(2-chlorophenyl)methanone(7c)

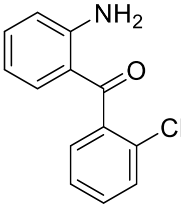

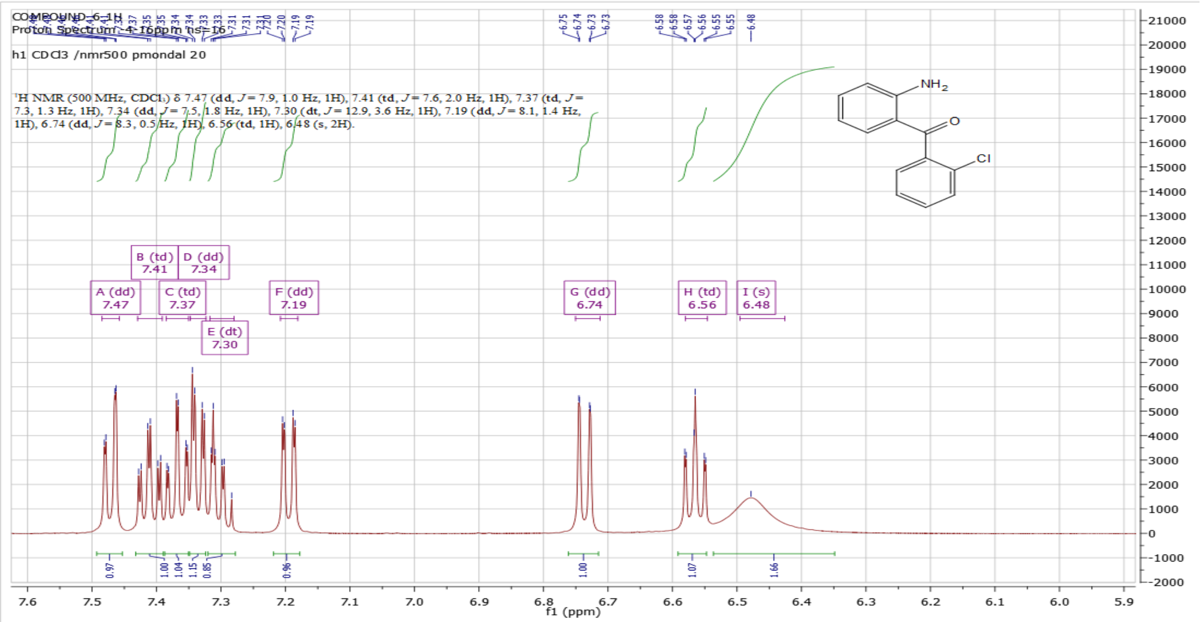

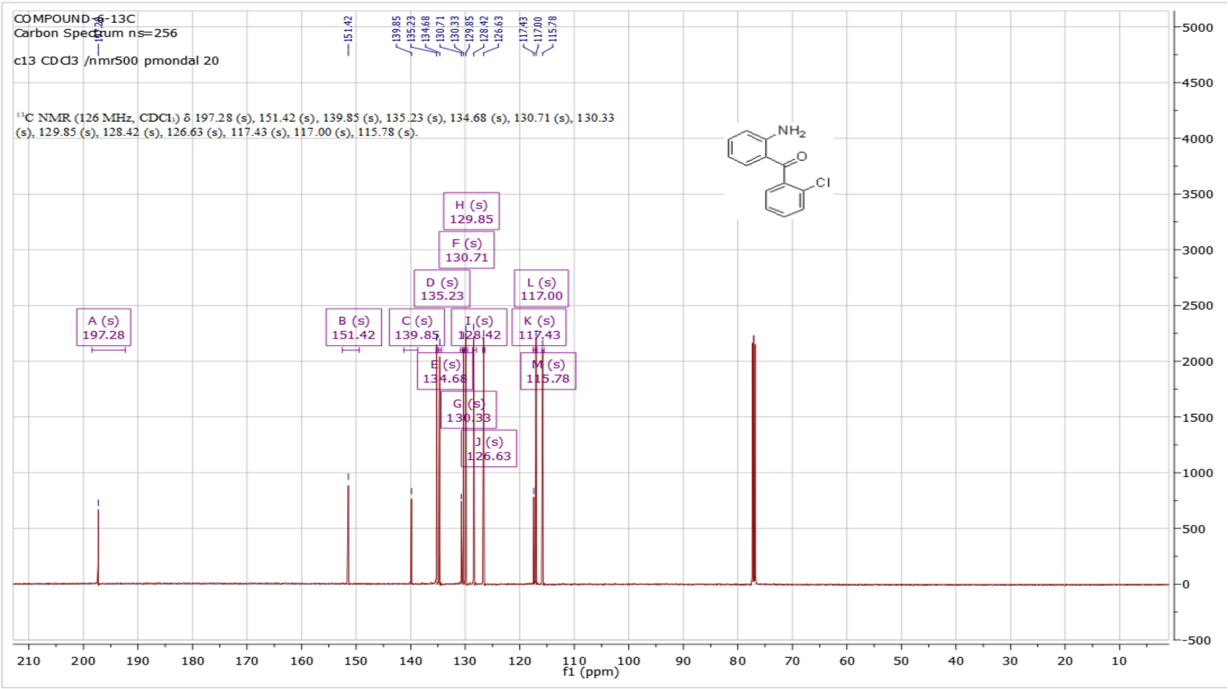

 ©AUTHOR(S)

Formula Predictor Report - COMP 6_12082020 Analysis_41.lcd Page 1 of 1

Data File: C:\LabSolutions\Data\Vilashini Rajaratnam\12082020 Analysis\COMP 6_12082020 Analysis_41.lcd

Elmt	Val.	Min	Max	Elmt	Val.	Min	Max	Elmt	Val.	Min	Max	Elmt	Val.	Min	Max	Use Adduct
H	1	10	25	N	3	1	3	Si	4	0	0	Br	1	0	0	H
2H	1	0	0	O	2	1	3	S	2	0	0	I	3	0	0	K
C	4	10	25	F	1	0	1	Cl	1	0	1					

Error Margin (ppm): 300
HC Ratio: unlimited
Max Isotopes: all
MSn Iso RI (%): 75.00

DBE Range: -100.0 - 2000.0
Apply N Rule: no
Isotope RI (%): 1.00
MSn Logic Mode: AND

Electron Ions: both
Use MSn Info: yes
Isotope Res: 10000
Max Results: 10

Event#: 1 MS(E+) Ret. Time : 0.893 -> 0.973 - 1.227 -> 1.238 Scan# : 135 -> 147 - 185 -> 187

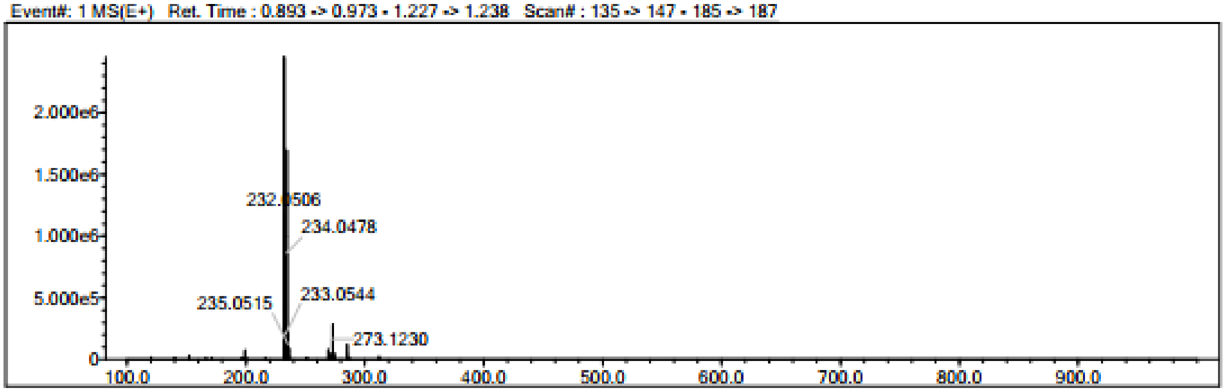

Measured region for 232.0506 m/z

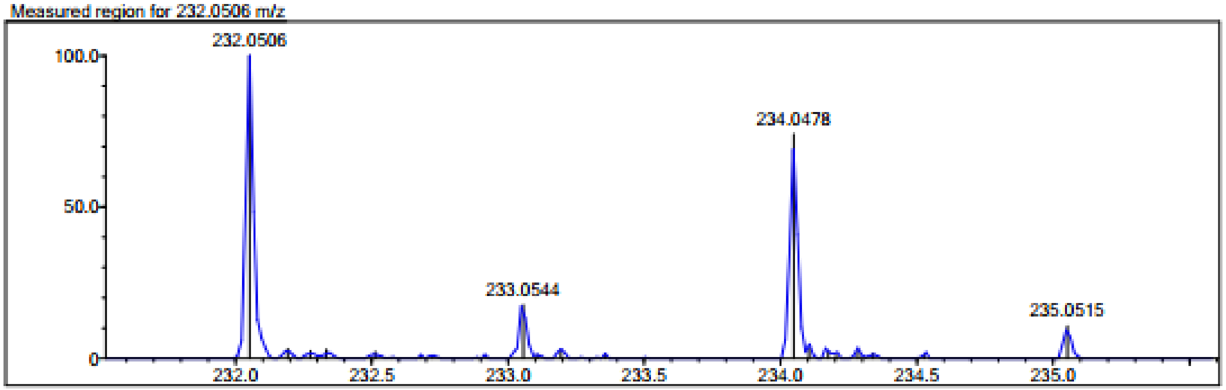

C13 H10 N O Cl [M+H]+ : Predicted region for 232.0524 m/z

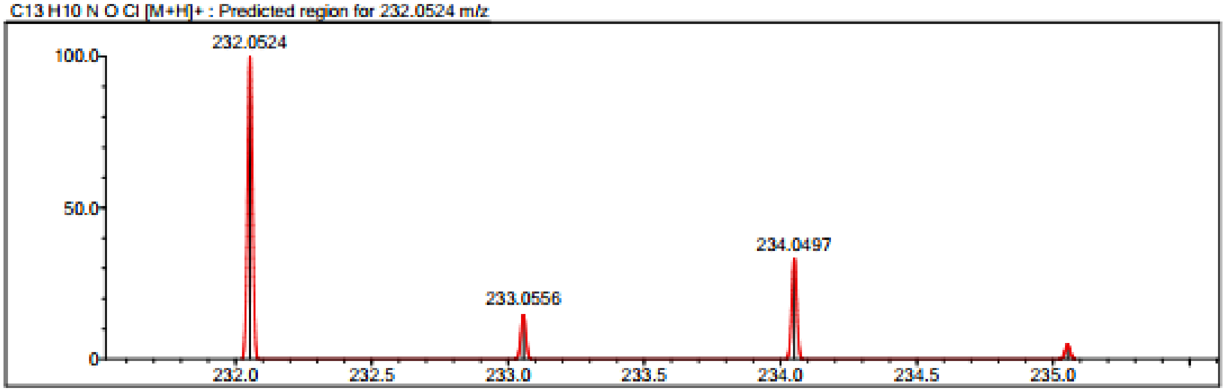

Rank	Score	Formula (M)	Ion	Meas. m/z	Pred. m/z	Df. (mDa)	Df. (ppm)	Iso	DBE
1	30.35	C13 H10 N O Cl	[M+H]+	232.0506	232.0524	-1.8	-7.76	48.64	9.0

(2-aminophenyl)(pyridin-2-yl)methanone (7d)

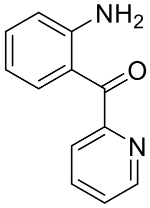

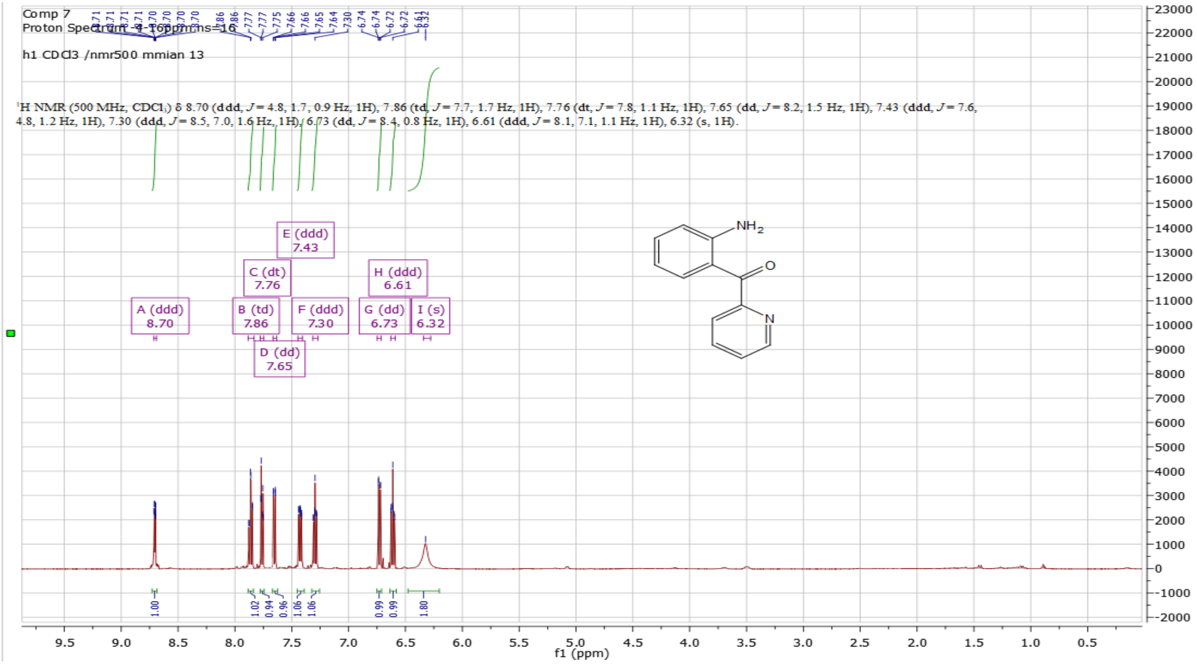

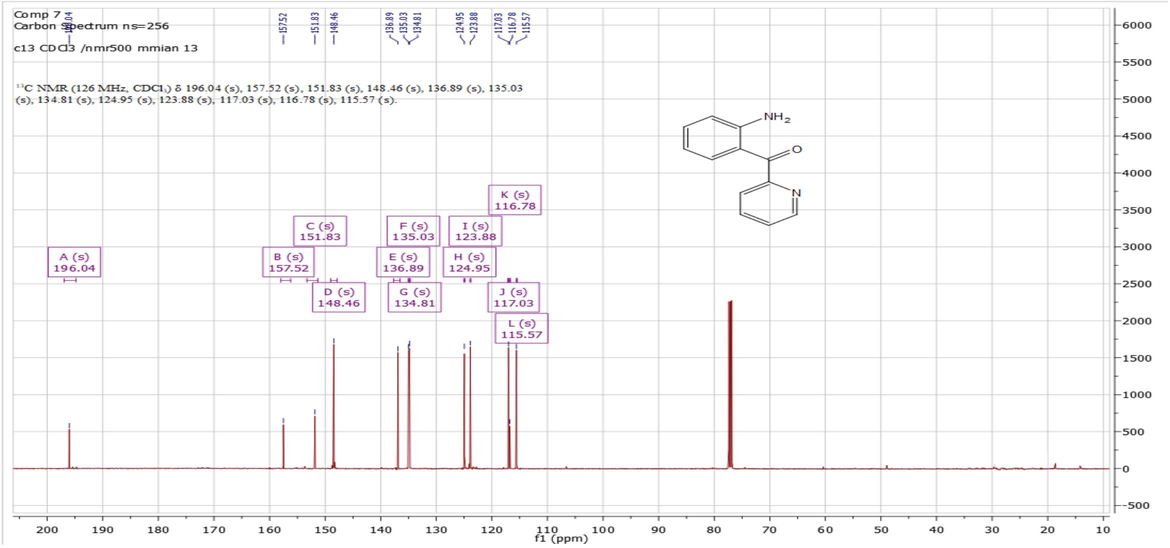

©AUTHOR(S)

Formula Predictor Report - COMP 7 _12082020 Analysis_39.lcd Page 1 of 1

Data File: C:\LabSolutions\Data\Vilashini Rajaratnam\12082020 Analysis\COMP 7 _12082020 Analysis_39.lcd

Elmt	Val.	Min	Max	Elmt	Val.	Min	Max	Elmt	Val.	Min	Max	Elmt	Val.	Min	Max	Use Adduct
H	1	10	30	N	3	1	3	Si	4	0	0	Br	1	0	0	H
2H	1	0	0	O	2	1	3	S	2	0	0	I	3	0	0	K
C	4	10	25	F	1	0	1	Cl	1	0	1					

Error Margin (ppm): 300
HC Ratio: unlimited
Max Isotopes: all
MSn Iso RI (%): 75.00
DBE Range: -100.0 - 2000.0
Apply N Rule: no
Isotope RI (%): 1.00
MSn Logic Mode: AND
Electron Ions: both
Use MSn Info: yes
Isotope Res: 10000
Max Results: 10

Event#: 1 MS(E+) Ret. Time : 0.893 -> 1.640 - 0.040 -> 0.311 Scan# : 135 -> 247 - 7 -> 47

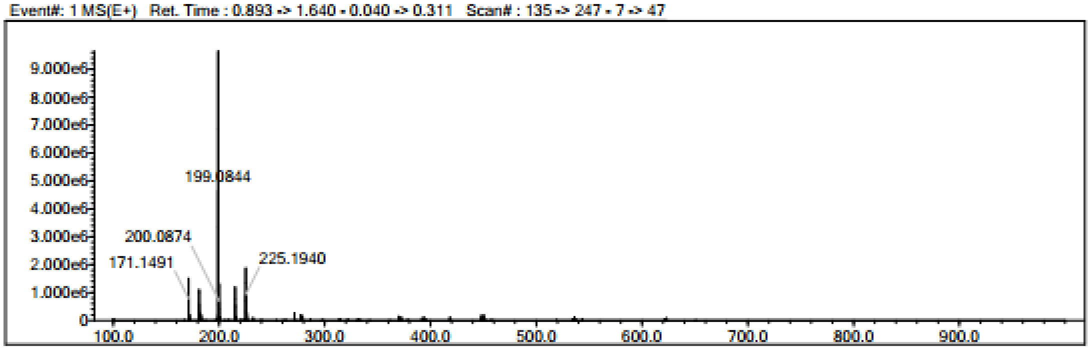

Measured region for 199.0844 m/z

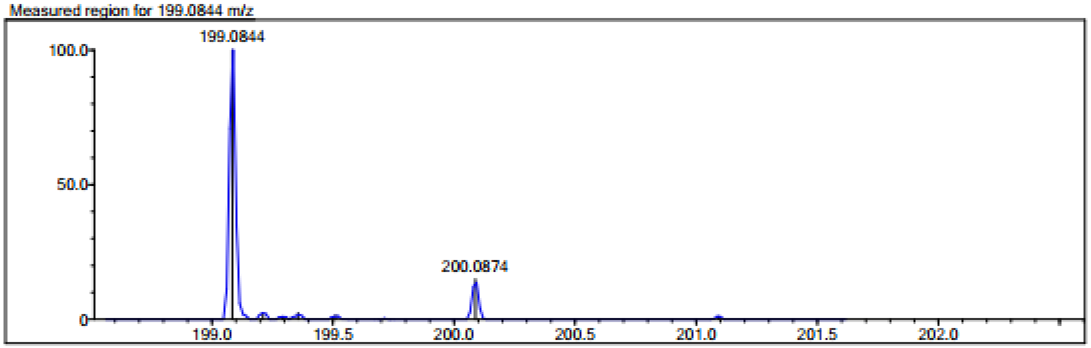

C12 H10 N2 O [M+H]+ : Predicted region for 199.0866 m/z

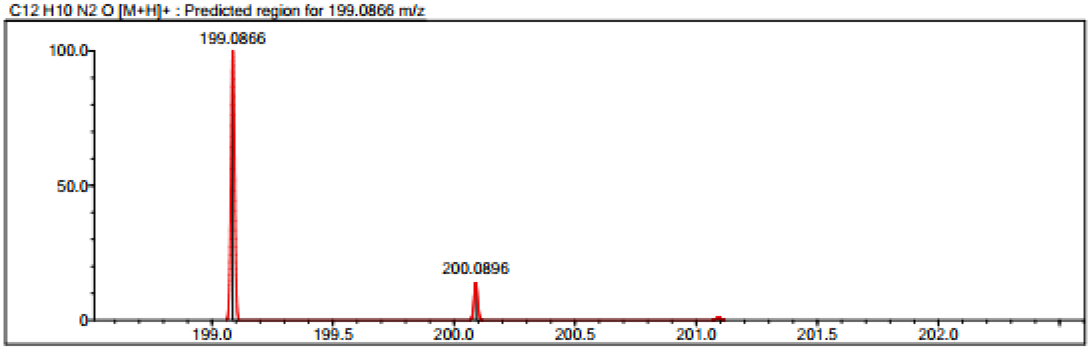

Rank	Score	Formula (M)	Ion	Meas. m/z	Pred. m/z	Df. (mDa)	Df. (ppm)	Iso	DBE
1	35.70	C12 H10 N2 O	[M+H]+	199.0844	199.0866	-2.2	-11.05	95.97	9.0

 ©AUTHOR(S)

ethyl (R)-6-(2-fluorophenyl)-4-methyl-4H-benzo[f]imidazo[1,5-a][1,4]diazepine-3-carboxylate(9a)

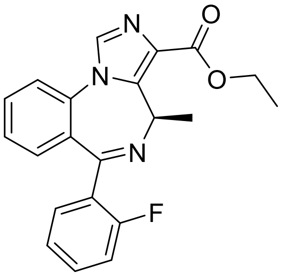

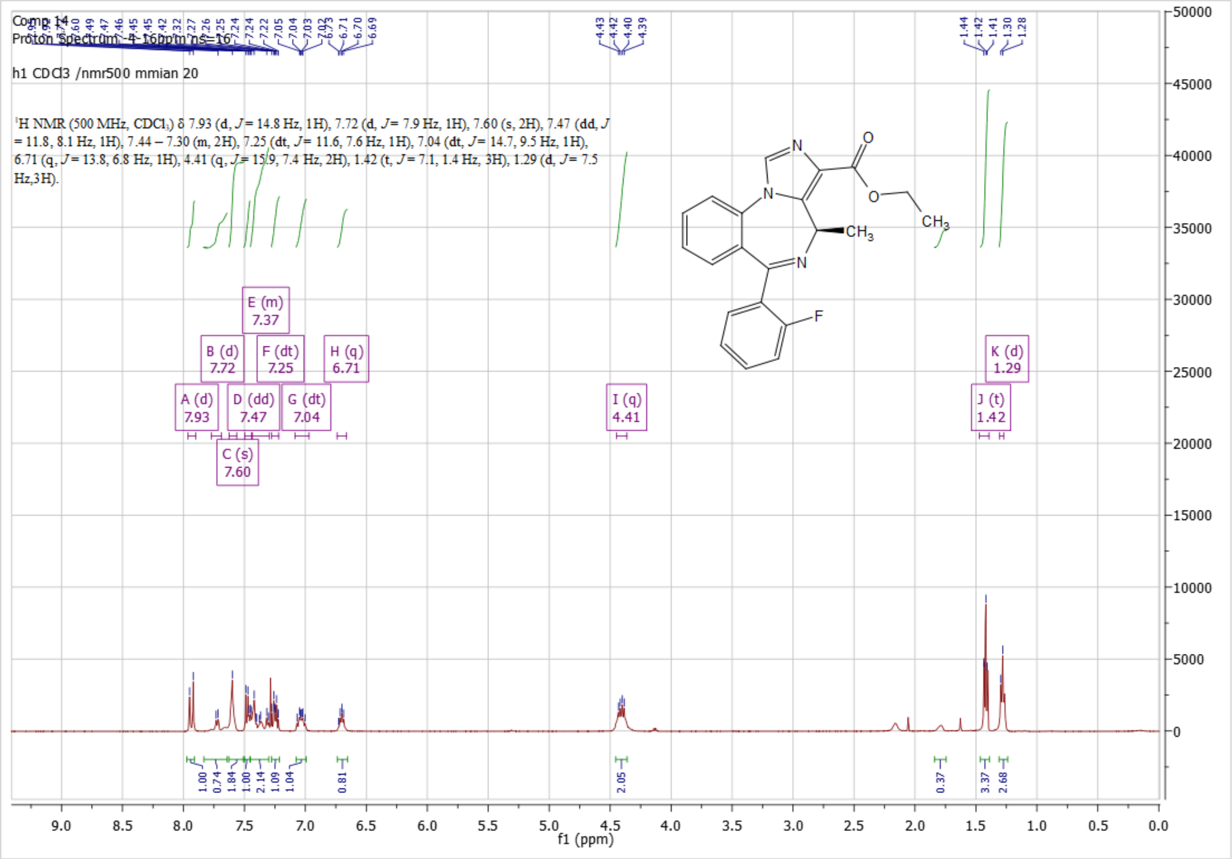

 ©AUTHOR(S)

-

Formula Predictor Report - COMP 14_12082020 Analysis_25.lcd Page 1 of 1

Data File: C:\LabSolutions\Data\Vilashini Rajaratnam\12082020 Analysis\COMP 14_12082020 Analysis_25.lcd

Elmt	Val.	Min	Max	Elmt	Val.	Min	Max	Elmt	Val.	Min	Max	Use Adduct
H	1	10	30	O	2	1	3	Cl	1	0	0	H
2H	1	0	0	F	1	1	4	Br	1	0	0	Na
C	4	10	25	Si	4	0	0	I	3	0	0	K
N	3	1	3	S	2	0	0					NH4

Error Margin (ppm):	300	DBE Range:	-100.0 - 2000.0	Electron Ions:	both
HC Ratio:	unlimited	Apply N Rule:	no	Use MSn Info:	yes
Max Isotopes:	all	Isotope RI (%):	1.00	Isotope Res:	10000
MSn Iso RI (%):	75.00	MSn Logic Mode:	AND	Max Results:	10

Event#: 1 MS(E+) Ret. Time : 0.907 -> 1.133 - 0.320 -> 0.467 Scan# : 137 -> 171 - 49 -> 71

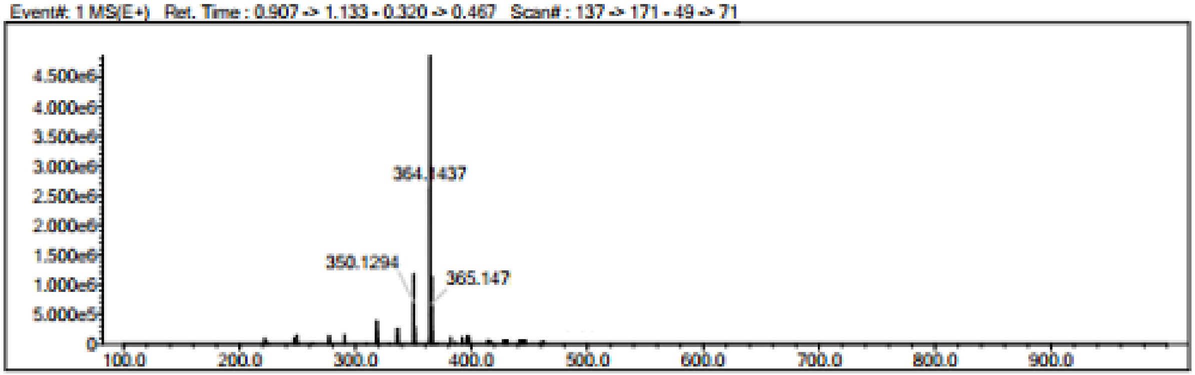

Measured region for 364.1437 m/z

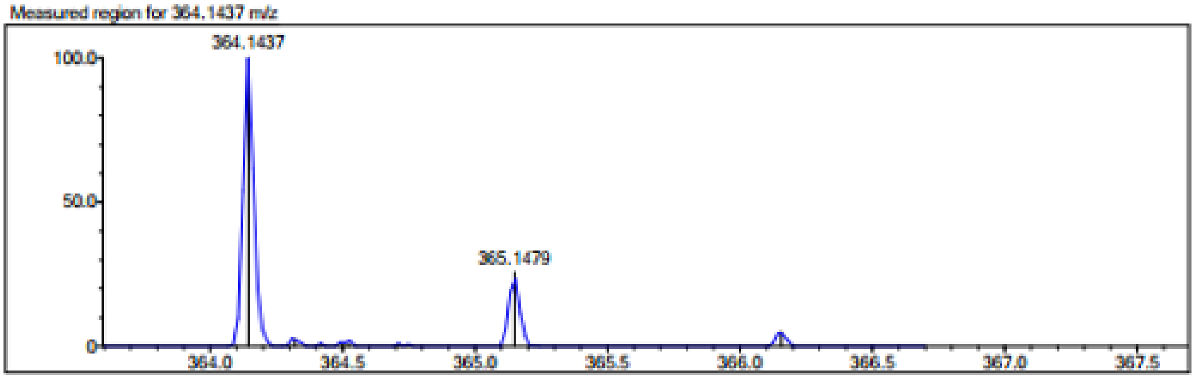

C21 H18 N3 O2 F [M+H]+ : Predicted region for 364.1456 m/z

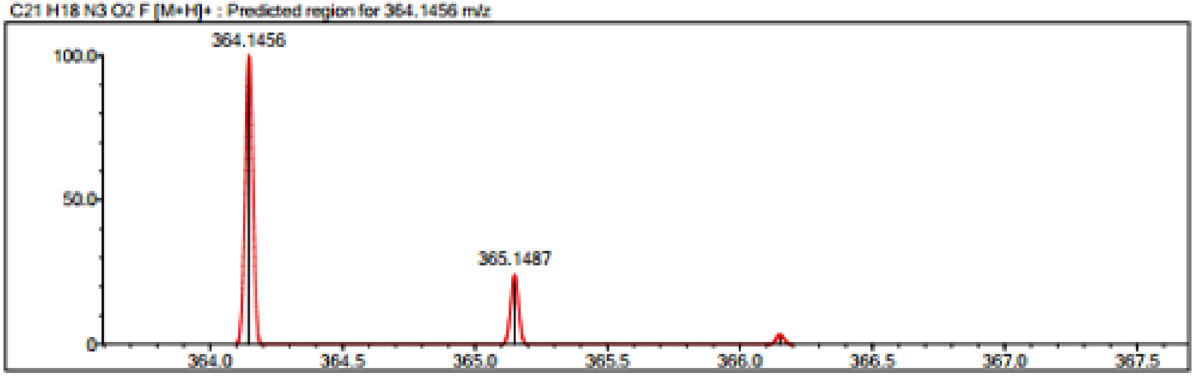

Rank	Score	Formula (M)	Ion	Meas. m/z	Pred. m/z	Df. (mDa)	Df. (ppm)	Iso	DBE
3	72.18	C21 H18 N3 O2 F	[M+H]+	364.1437	364.1456	-1.9	-5.22	82.21	14.0

 ©AUTHOR(S)

Debromination on a double bond containing substrate

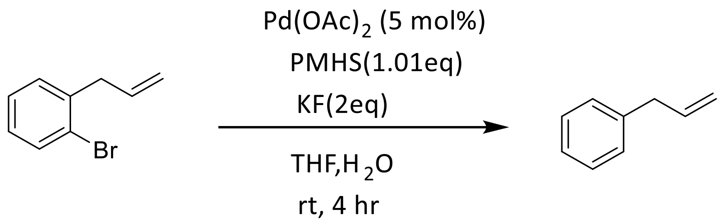

PHMS debrominated the bromine group selectively without interfering with the double bond. The debromination was confirmed by running the crude products on a single quadrupole LCMS 2020.

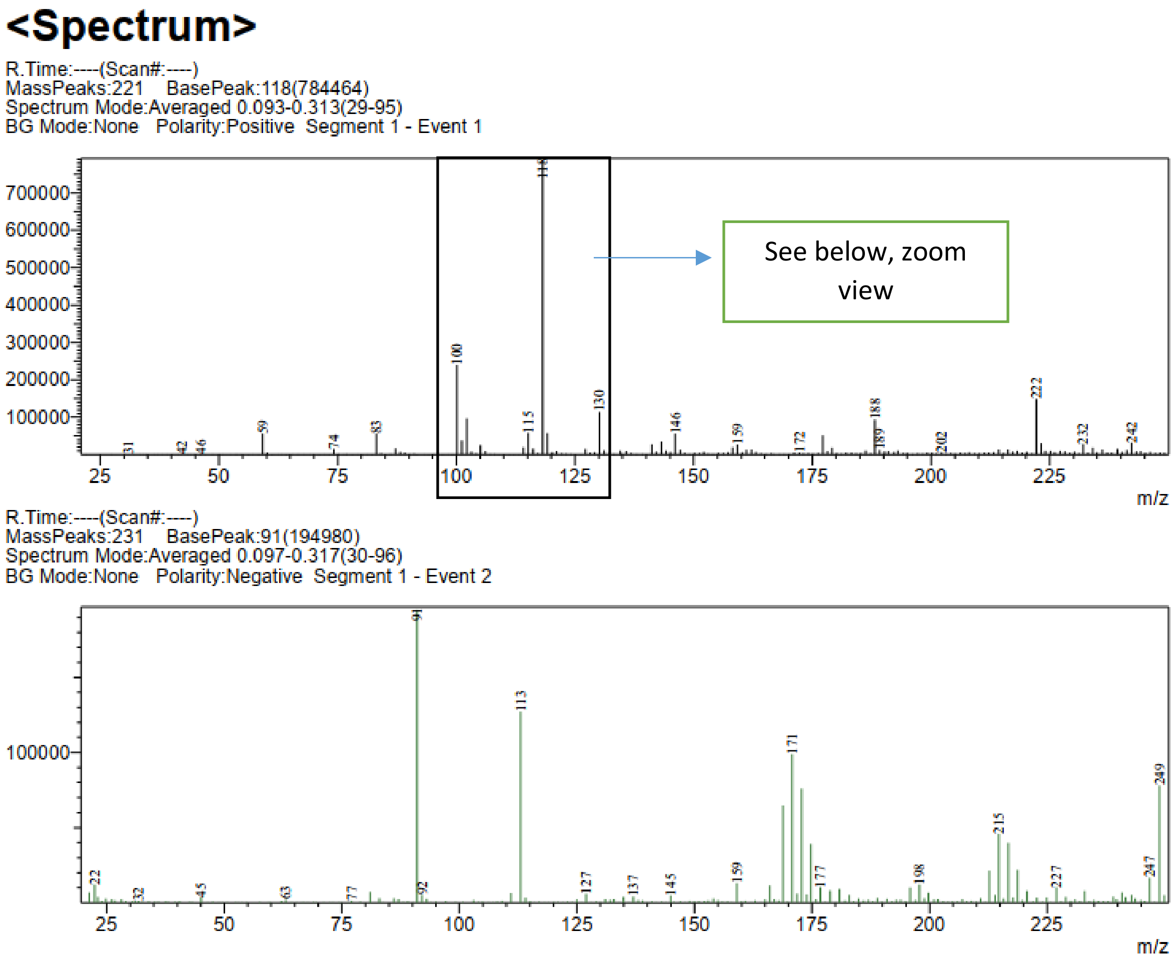

Prithu 2020 – 3-87/3-526-1 – 3.lcd

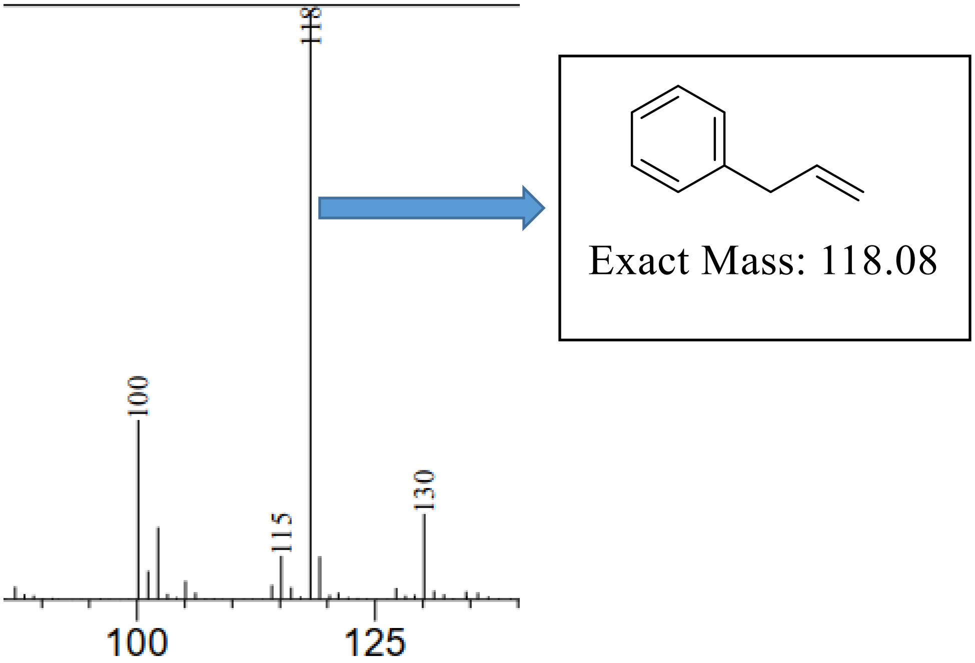

If double bond is reduced, then the mass will be 120, there is no peak here.

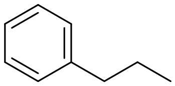

Exact Mass: 120.09

If double bond is reduced before debromination, then the mass will be 198 and a double peak will appear but there is no such peak in the whole spectra

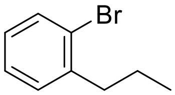

Exact Mass: 198.00

 ©AUTHOR(S)
